# *In vitro* methods to ensure absence of residual undifferentiated human induced pluripotent stem cells intermingled in induced nephron progenitor cells

**DOI:** 10.1371/journal.pone.0275600

**Published:** 2022-11-15

**Authors:** Hiraku Tsujimoto, Naoko Katagiri, Yoshihiro Ijiri, Ben Sasaki, Yoshifumi Kobayashi, Akira Mima, Makoto Ryosaka, Kenichiro Furuyama, Yoshiya Kawaguchi, Kenji Osafune

**Affiliations:** 1 Center for iPS Cell Research and Application (CiRA), Kyoto University, Kyoto, Japan; 2 Rege Nephro Co., Ltd., Med-Pharm Collaboration Building, Kyoto University, Kyoto, Japan; Affiliated Hospital of Jiangsu University, CHINA

## Abstract

Cell therapies using human induced pluripotent stem cell (hiPSC)-derived nephron progenitor cells (NPCs) are expected to ameliorate acute kidney injury (AKI). However, using hiPSC-derived NPCs clinically is a challenge because hiPSCs themselves are tumorigenic. *LIN28A*, *ESRG*, *CNMD* and *SFRP2* transcripts have been used as a marker of residual hiPSCs for a variety of cell types undergoing clinical trials. In this study, by reanalyzing public databases, we found a baseline expression of *LIN28A*, *ESRG*, *CNMD* and *SFRP2* in hiPSC-derived NPCs and several other cell types, suggesting *LIN28A*, *ESRG*, *CNMD* and *SFRP2* are not always reliable markers for iPSC detection. As an alternative, we discovered a lncRNA marker gene, *MIR302CHG*, among many known and unknown iPSC markers, as highly differentially expressed between hiPSCs and NPCs, by RNA sequencing and quantitative RT-PCR (qRT-PCR) analyses. Using *MIR302CHG* as an hiPSC marker, we constructed two assay methods, a combination of magnetic bead-based enrichment and qRT-PCR and digital droplet PCR alone, to detect a small number of residual hiPSCs in NPC populations. The use of these *in vitro* assays could contribute to patient safety in treatments using hiPSC-derived cells.

## Introduction

Currently, kidney transplantation is the only curative treatment for patients with end-stage renal failure by restoring kidney function. Due to the shortage of donor kidneys, however, regenerative medicine is eagerly awaited. A series of recent discoveries reported that human fetal nephron progenitor cells (NPCs) are effective for the treatment of chronic kidney disease (CKD) [1] and that human induced pluripotent stem cell (hiPSC)-derived NPCs can ameliorate acute kidney injury (AKI) in mice [2–4]. These findings have stimulated interest in cell therapies for CKD, in which AKI plays an important role [5]. NPCs are the source of nephron-constituent epithelia and form glomeruli and renal tubules by reciprocal interactions with the ureteric bud, a collecting duct progenitor tissue [6, 7]. These NPCs are considered to deliver a variety of nutritional factors that energize failing kidneys and produce direct and local paracrine effects [2].

However, using hiPSC-derived cell products clinically is a challenge because hiPSCs themselves are tumorigenic. Indeed, tumor formation has been associated with many stem cell therapies [8, 9]. Avoiding hiPSC contamination or residual hiPSCs requires a thoroughly controlled cell manufacturing process and assays that ensure the absence of hiPSCs. Several *in vitro* tests for this purpose exist [10–12], including flow cytometry [13], quantitative RT-PCR (qRT-PCR) [13], droplet digital PCR (ddPCR) [14, 15], culture methods for efficient hiPSC growth [15, 16], the detection of marker molecules released into the culture medium [17, 18], and enrichment using magnetic beads [15, 19].

The clinical application of NPCs has another challenge in terms of the absolute number of cells used. If the findings in mice are translated to human dosages, cells on the order of 10^9^ are needed [2]. In this case, even if a small proportion of residual hiPSCs are present, the absolute number would still risk tumor formation. Therefore, highly sensitive tests are required.

Accordingly, in this study, we discovered a suitable marker, *MIR302CHG*, among many known and unknown iPSC markers, for the detection of residual hiPSCs and developed assays to confirm the absence of residual undifferentiated hiPSCs intermingled in hiPSC-derived NPC populations.

## Results

### Expression of *LIN28A*, *ESRG*, *CNMD* and *SFRP2* in various cell types

Since highly sensitive hiPSC detection methods using *LIN28A*, *ESRG*, *CNMD* and *SFRP2* as hiPSC markers have been reported for retinal pigment epithelial cells [13], dopaminergic progenitor cells [11], cardiomyocytes [14], T cells [15] and liver bud [20], we analyzed the expression of these four genes in public RNA-seq datasets of various human fetal tissues [21] using GREIN [22]. We found similarly low expressions of *LIN28A* in the fetal kidney, liver, and pancreas as in the heart and similarly low expressions of *CNMD* and *SFRP2* in the kidney and pancreas as in the liver (S1A Fig). Next, we obtained publicly available RNA-seq datasets of these cell types but derived from hiPSCs. We found the expressions of *LIN28A*, *ESRG*, *CNMD* and *SFRP2* varied at different differentiation stages, with some stages expressing relatively high *LIN28A* in hiPSC-derived liver cells [23], pancreatic progenitor cells [24], liver organoids [25], and NPCs [26] (S1B–S1E Fig) and others expressing high *ESRG*, *CNMD* and *SFRP2* in hiPSC-derived kidney organoids [26] (S1E Fig). These findings suggested that *LIN28A*, *ESRG*, *CNMD* and *SFRP2* are not suitable markers for detecting residual hiPSCs among several cell types. However, since those datasets are based on tissues produced from hiPSCs in laboratories and the manufacturing process is not as strictly controlled as in a cell conditioning facility, the possibility of hiPSC contamination during the sample preparation cannot be completely ruled out.

### Expression of *LIN28A*, *CNMD* and *SFRP2* in purified NPCs

**Fig 1:**
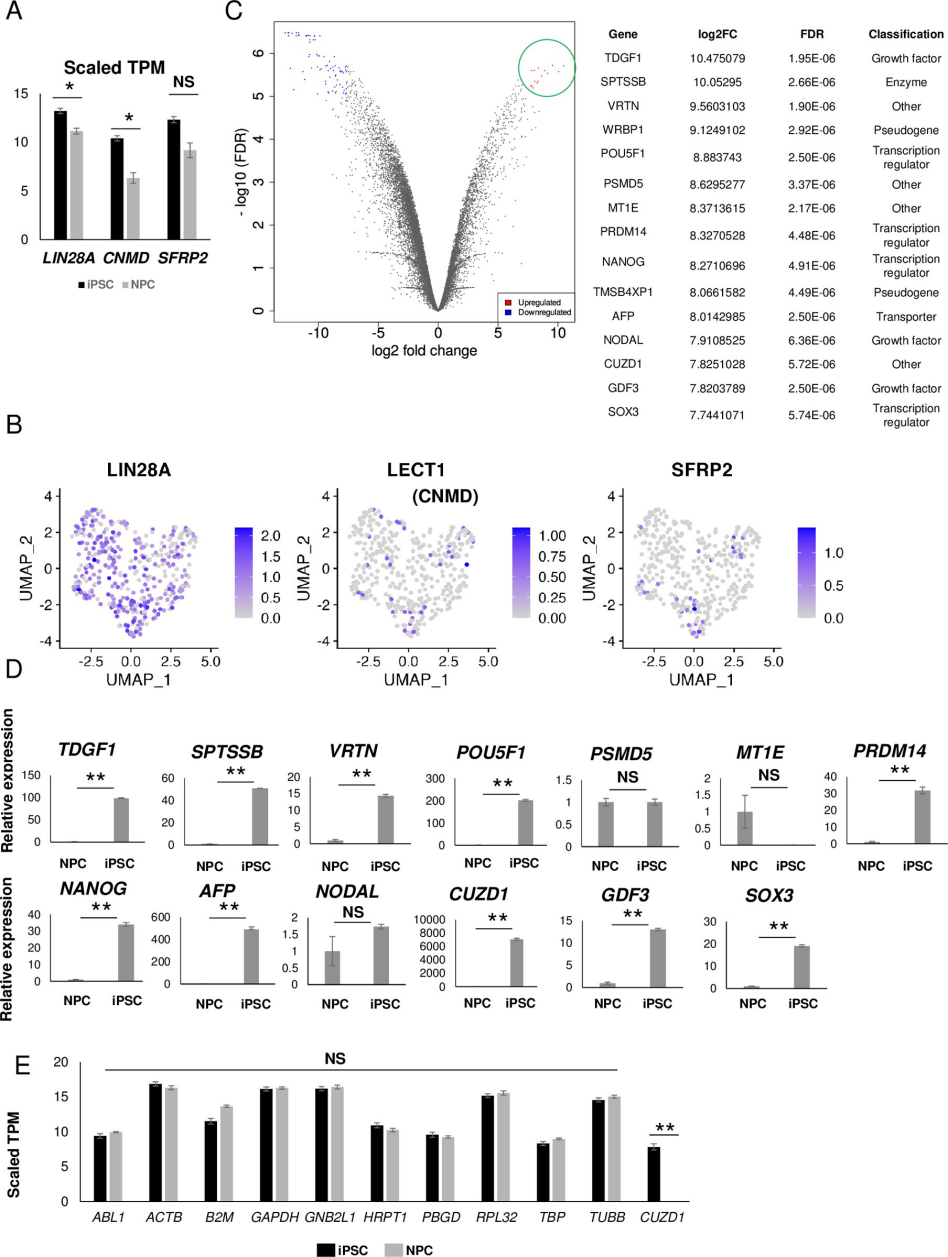
Identification of markers highly expressed in human induced pluripotent stem cells (hiPSCs) compared to purified OSR1(+)SIX2(+) induced nephron progenitor cells (NPCs). (A) Scaled TPM values of *LIN28A*, *CNMD* and *SFRP2* in hiPSCs and NPCs. (B) Uniform Manifold Approximation and Projection (UMAP) plots for *LIN28A*, *LECT1* (*CNMD*), *SFRP2* expression in the scRNA-seq data of purified OSR1(+)SIX2(+) NPCs reported in Tsujimoto et al. (2020). (C) A volcano plot (left panel) of differentially regulated genes at log10(FDR) > 5 and log2(fold change) < − 7 (blue dots) or > 7 (red dots) and a table (right panel) of 15 candidate iPSC markers as genes with log10(FDR) > 5 and log2(fold change) > 7 (green circle). The gene classification by IPA is shown. (D) qRT-PCR analysis of the 13 candidate protein-coding iPSC markers. Each value was normalized to that of NPCs. (E) Scaled TPM values of 10 housekeeping genes and *CUZD1* in hiPSCs (black bars) and NPCs (grey bars). Data are represented as the mean ± SEM (n = 3) in (A), (D) and (E). *p <0.05 and **p <0.01 by paired Student’s t-tests against iPSCs with the Bonferroni correction. NS, not significant.

We next examined the expressions of *Lin28A*, *Cnmd* and *Sfrp2*, which encode proteins in mouse developing kidneys using GUDMAP, a publicly available molecular atlas of gene expressions for developing organs of the genitourinary tract [27], and confirmed relatively high *Lin28A* and *Sfrp2* and *Cnmd* expression in E11.5 metanephric mesenchyme and podocytes, respectively (S2 Fig). To confirm the expression of *LIN28A*, *CNMD* and *SFRP2* in our induced NPCs, we performed a differentially expressed gene (DEG) analysis of deposited bulk RNA-seq data for purified hiPSC-derived OSR1-GFP(+)/SIX2-tdTomato(+) NPCs [2, 28] and 201B7 hiPSCs [29, 30] using Salmon [31], which quantifies transcript abundance using cDNA sequences. We used Homo sapiens GRCh38 cDNA gene annotation (Ensembl), which covers all transcripts of Ensembl genes excluding ncRNA. An enrichment analysis using the gene ontology (GO) biological process for DEGs showed that gene sets related to kidney development (cluster B) were upregulated in NPCs, while gene sets related to stem cell maintenance (cluster A) were upregulated in hiPSCs (S3A Fig). These findings suggest that although the sequence datasets of hiPSCs and NPCs were from different origins, the analysis correctly reflected DEGs. We then confirmed that the difference in average scaled TPM values for *LIN28A*, *CNMD* and *SFRP2* between purified hiPSC-derived OSR1-GFP(+)/SIX2-tdTomato(+) NPCs and hiPSCs was not large, although the difference in *LIN28A* and *CNMD1* was statistically significant (Fig 1A).

We also re-analyzed our previously reported single-cell RNA-seq (scRNA-seq) data of purified hiPSC-derived OSR1-GFP(+)/SIX2-tdTomato(+) NPCs [29] and found that the cells expressing these genes are distributed unbiasedly compared to the distribution of the cells expressing NPC marker genes in the dataset (Figs 1B and S4). An analysis of correlation between the expression of these genes and NPC markers (*SIX2* and *PAX2*) showed weak or almost no correlation (S4F Fig). Thus, these data suggest that no specific NPC populations expresses of *LIN28A*, *CNMD* and *SFRP2*. The qRT-PCR analysis also showed non-negligible *LIN28A* expression when we induced NPCs using an HLA homozygous hiPSC line, Ff-I 14s04 (S3B–S3D Fig). Therefore, to detect hiPSCs intermingled in NPCs populations with high sensitivity, we sought new genetic markers that are highly expressed in hiPSCs compared to NPCs.

### Identification of genes highly expressed in hiPSCs compared to NPCs

We then analyzed the aforementioned bulk RNA-seq datasets of purified hiPSC-derived OSR1-GFP(+)/SIX2-tdTomato(+) NPCs and hiPSCs and extracted 15 genes with particularly high expression values (FC > 200 & FDR <10^−5^; Fig 1C) in hiPSCs. We selected 13 protein-coding genes and performed a qRT-PCR analysis to compare the expressions between NPCs and hiPSCs using the QHJI 14s04 iPSC line, which was derived from a clinical-grade iPSC bank [32]. Among them, *CUZD1* was markedly and differentially expressed in hiPSCs (Fig 1D). For a housekeeping gene, we selected *TBP*, which is stably expressed in NPCs and hiPSCs and similar to *CUZD1* in scaled TPM expression in hiPSCs (Fig 1E).

### Detection of undifferentiated hiPSCs in NPCs using *CUZD1* copy numbers determined by ddPCR

Next, to create a hypothetical situation where NPCs are contaminated with hiPSCs, we manually contaminated the NPCs with hiPSCs. By generating hiPSCs constitutively expressing a red fluorescent protein, tdTomato, we confirmed that the percentages calculated from the cell count and the actual percentage measured by flow cytometry were almost identical (S5A Fig).

Next, we performed one-step ddPCR [14] on hiPSCs mixed at a ratio of 1:10^4^ with NPCs. However, we could not achieve stable and sensitive detection (S5B Fig). We considered RNA degradation during the droplet preparation as the reason. Therefore, we switched to a two-step ddPCR, in which cDNA is used during the droplet preparation and PCR reaction and further normalization using a housekeeping gene is performed. For the housekeeping gene, we selected TBP, which shows relatively low expression, to reduce possible artifacts of the multiplex PCR reaction (Fig 1D). As a result, while the 1:10^4^ ratio might be sufficient to discriminate hiPSCs according to the estimated copy number ratio (estimated CUZD1 copy number/estimated TBP copy number) evaluated by ddPCR, we expected slight experimental errors would make them indistinguishable (S5C Fig). To further clarify the sensitivity of the ddPCR assay, we performed a ddPCR analysis using cDNAs derived from mixtures of hiPSCs and NPCs at various ratios (10^−6^:1 to 1:1). The *TBP*-normalized *CUZD1* expression pattern suggested limited assay performance when the iPSC proportion was low (S5D Fig). Therefore, we sought the differentiation stage when *CUZD1* expression was lowest, finding it was in day 4 cells (S6 Fig). Therefore, we also performed the assay using day 4 cells, but found only limited improvement (S5E Fig).

### Identification of lncRNA that has super-high differential expression in hiPSCs compared to NPCs or day 4 cells

**Fig 2:**
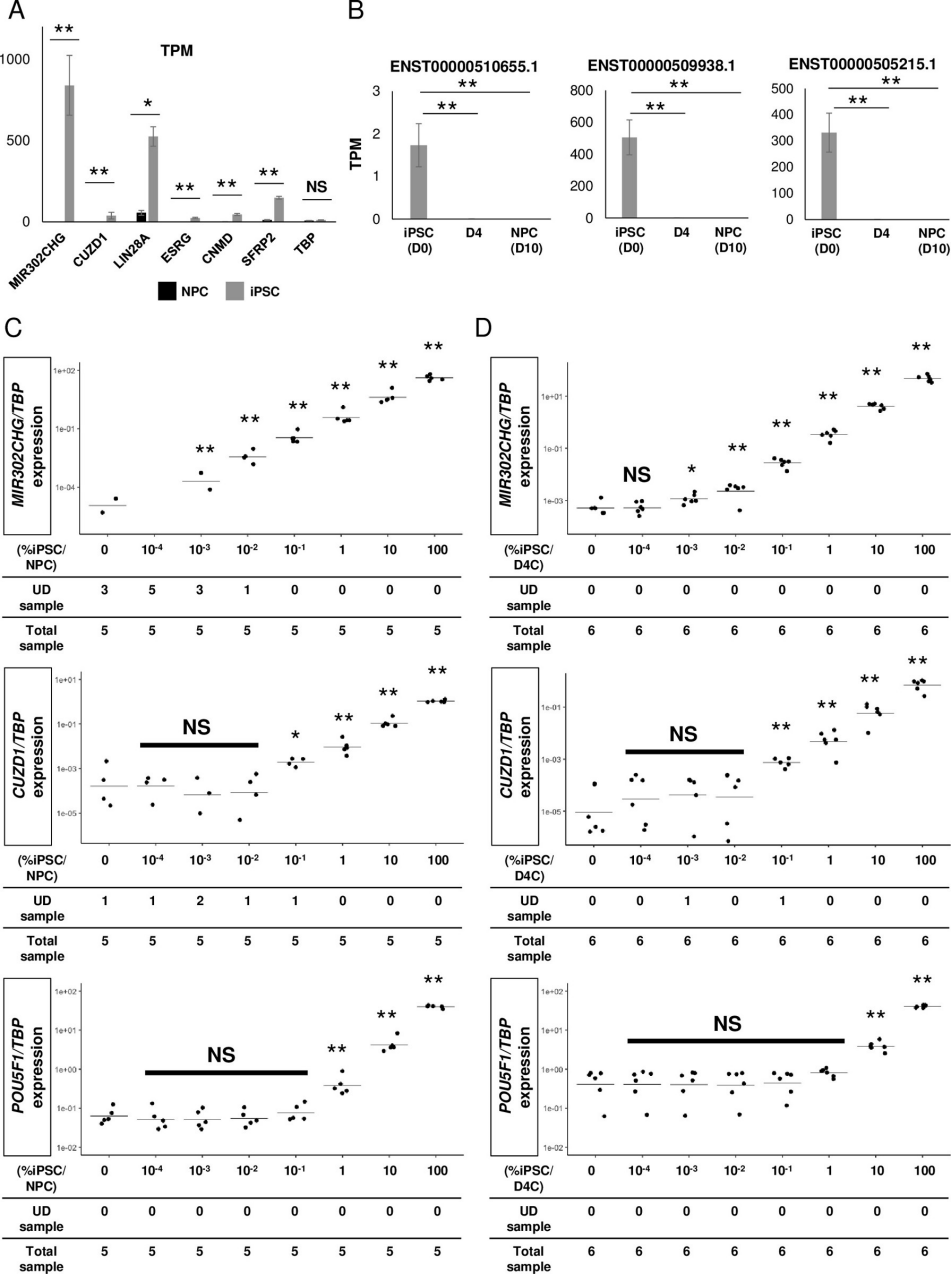
Identification of markers highly expressed in hiPSCs compared to NPCs derived from a clinical-grade iPSC stock line. (A) TPM values of *MIR302CHG*, *CUZD1*, *LIN28A*, *ESRG*, *CNMD*, *SFRP2* and *TBP* in hiPSCs and NPCs show the high absolute expression of *MIR302CHG*. Data are represented as the mean ± SEM (n = 3). (B) TPM values of three *MIR302CHG* splice variants, *ENST00000510655*.*1*, *ENST00000509938*.*1* and *ENST00000505215*.*1*, in hiPSCs and NPCs. Data are represented as the mean ± SEM (n = 3). (C, D) Scatter plots of the *TBP*-normalized gene expression of *MIR302CHG*, *CUZD1* and *POU5F1* by qRT-PCR for mixtures of hiPSCs and NPCs (C) or day 4 cells (D). The dots and lines in the center of the scatter plots indicate the experimental data and mean values of the data, respectively. UD: undetermined; D4C: day 4 cell. *p <0.05 and **p <0.01 by paired Student’s t-tests against iPSCs with the Bonferroni correction (A, B) and by Tukey-Kramer post hoc tests against the sample with 0% iPSCs (C, D). NS, not significant.

Based on the results of the ddPCR, we suspected that NPCs had a trace expression of *CUZD1*. Publicly available RNA-seq datasets of 27 different human tissues also showed a trace expression of *CUZD1* in several tissues including the kidney (S7A Fig) [33]. Thus, to identify markers that have a high expression in hiPSCs, but not in NPCs, we performed RNA-seq with increased sequencing depth. In addition, we applied a genomic mapping method using STAR [34] to extend our analysis to markers that do not encode proteins. The average leads per sample was 70M. We identified 26 extremely highly expressed genetic markers (log2FC > 10 & FDR <10^−5^) (S7B Fig) and found a pluripotency-associated lncRNA, *MIR302CHG* [35], exhibited higher TPM values in hiPSCs than either *LIN28A* or *CUZD1* (Fig 2A). Unexpectedly, we found *MIR302CHG* was highly differentially expressed in hiPSCs compared to both NPCs (S7B Fig) and day 4 cells from the NPC differentiation protocol (log2FC: 15.9 & FDR: 8.9×10^−30^; S1 Table). *MIR302CHG* is highly expressed not only in the QHJI 14s04 line but also in several hiPSC lines, including 1231A3 [36], 1383D6 [36], 201B7 [37] and 317–12 [38] (S7C Fig). An isoform analysis revealed that hiPSCs, human embryonic stem cells (hESCs), anterior primitive streak (PS) and definitive endoderm (DE) highly expressed splice variants 2 (ENST00000509938.1) and 3 (ENST00000505215.1) of *MIR302CHG* compared to differentiated cells such as pancreatic progenitors, foregut, hepatocytes, liver organoids, neural progenitors, neurons, cardiomyocytes, lung progenitors, lung organoids and endothelial cells (Figs 2B and S7D and S8–S10). Fortunately, since this lncRNA has a poly(A) 3’ tail (RNA central, release 19), we could perform a reverse transcription (RT) reaction using oligo deoxythymine (dT). Next, we performed qRT-PCR using a mixture of cDNAs derived from hiPSCs and NPCs or day 4 cells at various percentages (100% to 10^−4^%). The *TBP*-normalized *MIR302CHG* expression patterns showed better performance than the expression of *CUZD1* or *POU5F1* when the hiPSC percentages were low (Fig 2C and 2D and S1 Data). However, we sporadically obtained undermined CT values at low hiPSC percentages, suggesting a need for further improvement.

### Improvement of the qRT-PCR assay by magnetic bead-based enrichment

To overcome the difficulty in detecting a very small amount of hiPSCs, we tried iPSC enrichment using the hiPSC culture assay [15]. However, the assay was not suitable for NPCs because NPCs can grow in hiPSC culture media (Essential 8 or AK03N) (S11 Fig).

**Fig 3:**
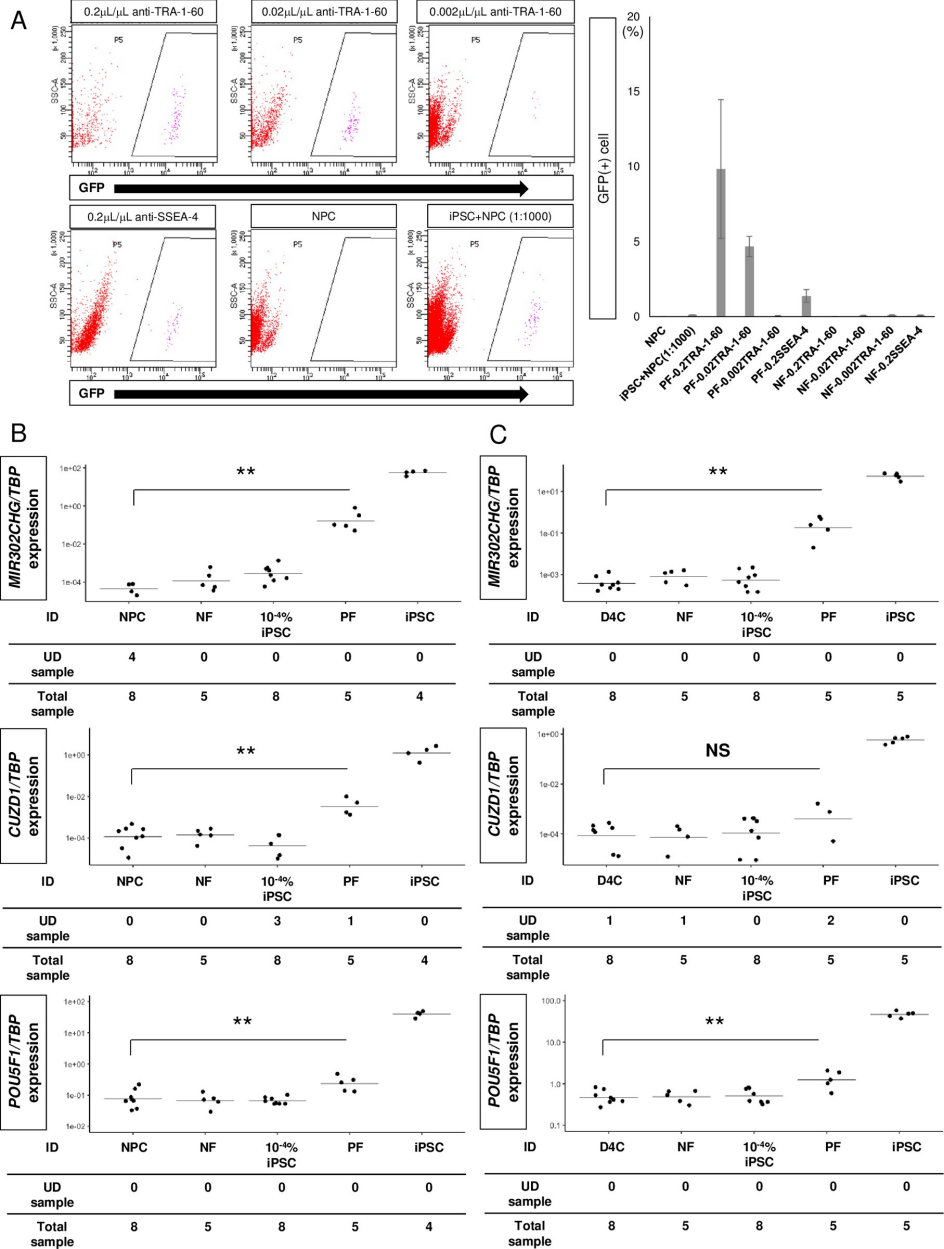
Combination assay using magnetic bead-based cell isolation and qRT-PCR analysis to detect hiPSCs intermingled in NPCs derived from a clinical-grade iPSC stock line. (A) Flow cytometric analysis of the MACS positive fractions of 1:1,000 mixtures of GFP (+) hiPSCs and GFP (-) NPCs at three different concentrations of anti-TRA-1-60 antibody-conjugated beads (upper panels) and one concentration of anti-SSEA-4 antibody-conjugated beads (lower left panel), GFP (-) NPCs (without MACS selection; lower center panel), and a 1:1,000 mixture of GFP (+) hiPSCs and GFP (-) NPCs (without MACS selection; lower right panel). The bar graph in the right panel shows the mean ± SEM of GFP (+) cells from the flow cytometric analysis at various bead concentrations in staining reagent. PF: positive fraction; NF: negative fraction; 0.2TRA-1-60: 0.2 μL TRA-1-60 beads / 1 μL staining reagent; 0.02TRA-1-60: 0.02 μL TRA-1-60 beads / 1 μL staining reagent; 0.002TRA-1-60: 0.002 μL TRA-1-60 beads / 1 μL staining reagent; 0.2SSEA-4: 0.2 μL SSEA-4 beads / 1 μL staining reagent. (B) Scatter plots of the *TBP*-normalized gene expressions of *MIR302CHG*, *CUZD1* and *POU5F1* in NPCs (NPC), the MACS negative fraction of the mixture (NF), 0.0001% hiPSC/NPC mixtures (10^−4^% iPSC), the MACS positive fraction of the mixture (PF), and hiPSCs (iPSC) by qRT-PCR analysis. (C) Scatter plots of the *TBP*-normalized gene expressions of *MIR302CHG*, *CUZD1*, and *POU5F1* in day 4 cells (D4C), NF, 10^−4^% iPSC, PF and iPSC by qRT-PCR. The dots and lines in the center of the scatter plots in (B) and (C) indicate the experimental data and mean values of the data, respectively. UD: undetermined. *p <0.05 and **p <0.01 by Tukey-Kramer post hoc tests against the sample with NPCs. NS, not significant.

Alternatively, we tested hiPSC enrichment using magnetic beads conjugated with antibodies against two well-known iPSC surface antigens, TRA-1-60 and SSEA-4 [15]. Using cell mixtures containing hiPSCs constitutively expressing EGFP (317–12 cells) [38] and NPCs derived from a non-fluorescent hiPSC line (QHJI 14s04), we evaluated the degree of magnetic separation of the positive and negative fractions by FACS using GFP as an indicator. As a result, we successfully enriched hiPSCs in NPC populations using beads conjugated with antibodies against TRA-1-60 but not SSEA-4 (Fig 3A).

Next, we prepared cell mixtures containing 1–3×10^2^ hiPSCs in 1–3×10^8^ NPCs or day 4 cells (0.0001%) and enriched hiPSCs with anti-TRA-1-60 antibody-conjugated magnetic beads and confirmed a marked increase in the *MIR302CHG*/*TBP* ratio upon qRT-PCR analysis (Fig 3B and 3C and S1 Data). We also confirmed that the expression values of *MIR302CHG*/*TBP* were below detection level in cDNAs derived from the positive fractions when NPCs alone were treated with the magnetic beads (S12 Fig). These results confirmed high sensitivity for hiPSC detection (0.0001% hiPSCs in NPCs) by qRT-PCR for *MIR302CHG* following MACS enrichment using anti-TRA-1-60 antibody-conjugated beads.

### Improvement of the qRT-PCR assay by ddPCR

**Fig 4:**
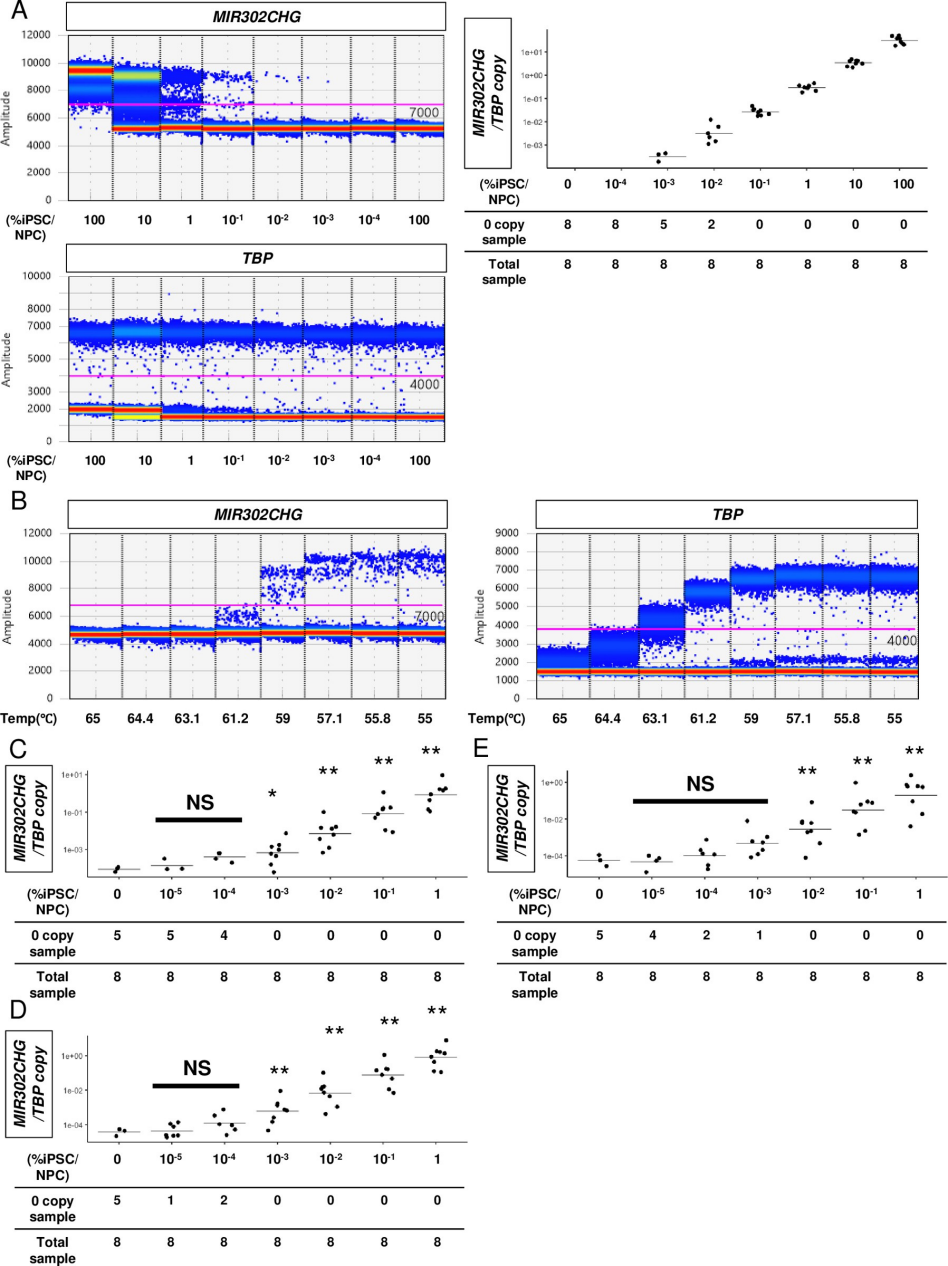
Development of a digital droplet PCR assay for hiPSCs intermingled in NPCs derived from a clinical-grade iPSC stock line. (A) Scatter plots of positive and negative droplets for digital droplet PCR (ddPCR) in an hiPSC/NPC cDNA dilution series sample. The purple lines indicate the cutoff values (left panels). A ddPCR analysis of *MIR302CHG* using an hiPSC cDNA dilution series sample in NPC cDNA shows a concentration-dependent change in the *TBP*-normalized copy number of *MIR302CHG* (right panel). (B) A representative result of ddPCR at annealing temperatures of 55°C to 65°C using 0.1% hiPSC/NPC cDNA samples shows a better separation of the positive and negative droplets of *MIR302CHG* and that *TBP* varies at an annealing temperature below 57.1°C. (C-E) Scatter plots of the ddPCR-estimated copy number ratios of *TBP*-normalized *MIR302CHG* using 5% (C), 15% (D) and 30% (E) of the RT products in the ddPCR reaction mix. *p <0.05 and **p <0.01 by the Tukey-Kramer post hoc tests against the sample with 0% hiPSCs. NS, not significant.

Although magnetic bead-based enrichment improves the sensitivity, the enrichment needs to be performed on the same day of the cell harvesting. Therefore, we also developed a method that can be performed only with cDNAs that can be stably cryopreserved. Using two-step ddPCR with the PCR parameters suggested by the manufacturer, we confirmed the *MIR302CHG*/*TBP* ratios evaluated by the ddPCR assay were generally proportional to the cDNA concentration ratios of hiPSCs in the NPC populations (Fig 4A). By optimizing the temperature (Fig 4B) and the loading amount of RT products (Fig 4C–4E and S1 Data), further improvement of the hiPSC detection was achieved, such that we could detect hiPSCs making up 0.001% of NPC populations without the magnetic bead-based enrichment (Fig 4D).

## Discussion

In this study, we examined protein-coding genes and comprehensively compared their gene expressions in hiPSCs and NPCs. By experimentally examining their usefulness as hiPSC markers, we showed that *CUZD1* did not perform as expected, probably due to its low absolute expression in hiPSCs. However, we confirmed experimentally that the lncRNA *MIR302CHG*, a non-protein encoding gene and known undifferentiated hESC marker in the MIR302/367 cluster host gene [39, 40], is a good marker. Previous reports have shown that the transduction of MIR302/367 using lentivirus vectors more efficiently reprograms human fibroblasts to the pluripotent state than the transduction of OCT3/4 (POU5F1), SOX2, KLF4 and MYC [35, 37] and that the direct transfection of a combination of MIR200c plus MIR302s and MIR369s family miRNAs reprograms human adipose stromal cells and fibroblasts to pluripotency [41]. Even for lncRNAs that do not encode proteins including *MIR302CHG* transcripts, a cDNA library can be synthesized and purified by RNA degradation after a RT reaction using poly dT sequences by taking advantage of the poly(A) 3’ tail. Accordingly, we discovered *MIR302CHG* is a suitable marker for the detection of hiPSCs intermingled in hiPSC-derived NPC products. Using *MIR302CHG*, we developed highly sensitive assays to detect residual hiPSCs using two approaches. Both of these approaches ensured the absence of residual undifferentiated hiPSCs intermingled in hiPSC-derived NPC populations.

TRA-1-60 is commonly used to assess the pluripotency of hiPSCs and hESCs. The TRA-1-60 antibody recognizes the type 1 lactosamine structure of podocalyxin [42, 43]. This glycan structure is different from that of podocalyxin expressed in the podocytes of kidneys [44]. Around 90% of the hiPSCs we used express the TRA-1-60 antigen (QHJI 14s04 Cell information). Although the discovery of novel hiPSC glycoproteins is in progress [45], the direct relationship between these antigens and tumorigenicity is unknown. Therefore, a detection method by ddPCR without using surface antigen enrichment is currently preferred.

In a previous report, a combination of magnetic bead-based enrichment and efficient culture assay could detect 10 (0.00002%) hiPSCs spiked into 5×10^7^ T cells [15]. Although the hiPSC culture assay is not suitable for NPCs because NPCs can grow in hiPSC culture media (Essential 8 or AK03N) (S11 Fig), by substituting the culture assay with a qRT-PCR assay, we achieved a comparably high sensitivity (0.0001% hiPSCs).

However, our method has several limitations. First, it is an *in vitro* assay and therefore should be combined with an *in vivo* tumorigenesis test. Ideally, future studies should investigate the minimum permissible level of the *MIR302CHG*/*TBP* ratio and the probability of tumorigenesis *in vivo* in large animals at doses similar to those in humans. Second, our study experimentally tested only hiPSC-derived NPCs and not absolute negative control cells, such as human embryonic NPCs. However, we are confident that our tested hiPSC-derived NPCs did not contain residual hiPSCs at levels higher than the desired sensitivity based on the results of the artificial contamination tests (Figs 3 and 4 and S12). The possible reason why we sporadically observed positive droplets even in samples without hiPSC addition (Figs 4 and S12) is that DNA fragments in the environment or residual DNA fragments after the DNase reaction could have been contaminated. Further improvement could be achieved by oligo dT bead purification and standardization of the loading amount for ddPCR using cDNA concentrations only derived from RNA with poly(A) 3’ tail. Third, we did not examine a method to remove the detected hiPSCs. Previous reports showed ways to eliminate hiPSCs intermingled in cardiomyocytes, neurons and hepatocytes using Orlistat [46] or Atorvastatin [47]. Future studies should develop such a method for NPCs. Fourth, our method cannot detect malignantly transformed cells associated with cultures, which are tumorigenic cells that form in cell preparations. Fifth, although we standardized and described the assay procedures in detail, the ddPCR and qRT-PCR results presented here are from a single institution (CiRA) and external validation at other institutions is also necessary. Finally, the sample size we used was small. The statistical analyses in Figs 3B and 3C and 4C and 4D tested whether the samples without hiPSCs show different values compared to the samples of interest. In future work, a cutoff value for the diagnosis of hiPSC contaminants should be established, and the sensitivity and specificity of the test at the cutoff value should be considered to guarantee the safety of the cell product. If these tests are to be used industrially, further validation will be necessary due to fluctuations in the manufacturing at cell conditioning facilities and the need to standardize the assays.

Despite the above, the use of the described *in vitro* assays will contribute to patient safety in treatments using hiPSC-derived cells.

## Materials and methods

### Ethics statement

Experiments using hiPSCs were approved by the ethics committee of the Center for iPS Cell Research and Application (CiRA), Kyoto University and performed according to the guidelines of the Declaration of Helsinki. No donors were from a vulnerable population, and all donors provided written informed consent that was freely given. We used 9 hiPSC lines including 5 parental hiPSCs (201B7, QHIJ 14s04, 585A1, 1231A3 and 1383D6) and their offspring hiPSCs (4A6, 317–12, Ff-I 14s04 and 585A1-tdTomato). The lines 201B7, 1231A3 and 1383D6 were established from human primary cells purchased from commercial cell distributors (201B7: Cell Applications, Inc., USA; 1231A3 and 1383D6: Cellular Technology Ltd, USA) in CiRA and widely distributed for biomedical research (e.g., RIKEN BioResource Research Center, https://web.brc.riken.jp/en/) [36, 37, 48]. 201B7 and the other 4 parental hiPSCs were established from dermal fibroblasts and peripheral blood mononuclear cells (PBMCs), respectively.

The PBMC donor recruitment was conducted from June 2008 to present including the donor of 585A1 and from January 2013 to July 2019 including the QHJI donor. Written informed consent was obtained from the QHJI donor (older than 20 years) from whom HLA homozygous hiPSCs were derived as part of a large initiative named ‘ Research on an HLA-Homozygous Donor-Derived iPS Cell Stock from Apheresis Donors for Regenerative Medicine’ (S1 File) [32, 49] and from an adult healthy donor (30–40 years old) from whom 585A1 (RIKEN BRC: HPS0354) and 585A1-tdTomato (this study) were derived as a part of another large initiative named ‘Genetic Analysis Study Using Human Disease-Specific iPS Cells’ and ‘The Generation of Human Disease-Specific iPS Cells and the Use of Such iPS Cells for Disease Analysis’ (S1 File) [50].

The donors of 585A1 and QHJI and their families did not receive monetary benefits. However, necessary research-related expenses were covered by the research fund of the initiatives. For the donors of human primary cells, which we purchased from commercial cell distributors (201B7, 1231A3 and 1383D6), no information about monetary reward was provided.

### Cell culture

hiPSCs were maintained with feeder-free cultures using Stem Fit AK02N medium (Ajinomoto) or Stem Fit AK03N medium (Ajinomoto) on cell culture plates coated with iMatrix [51]. The cells were passaged using 0.5 mM EDTA/PBS (Thermo Fisher Scientific) every four or five days and routinely tested for mycoplasma contamination. Details about the differentiation of hiPSCs towards NPCs are described elsewhere [29].

For the NPC growth assay using iPSC culture media, we used the OSR1-GFP/SIX2-tdTomato reporter iPSC-derived NPCs. NPCs were seeded into 96-well low cell-binding U-bottom plates (Thermo Fisher Scientific) at a density of 4.0 × 10^4^ NPCs/well with serum-free differentiation medium consisting of DMEM/F12 Glutamax (Thermo Fisher Scientific), B27 supplement minus vitamin A (Thermo Fisher Scientific) and 0.5×Penicillin/streptomycin (hereafter called basal medium) containing 1 μM CHIR99021 (Wako), 100 ng/mL FGF9 (Peprotech) and 10 μM Y-27632 to form aggregates. After spinning down the plate for 3 min at 200g, the cells were incubated for 24 h at 37°C. After the 24 h, the aggregates were subjected to iPSC growth media, Essential 8 medium (Thermo Fisher Scientific) or Stem Fit AK03N medium. The medium was changed every 1–2 days. We harvested NPC aggregates on days 1, 3, 5, 8 and 10. The aggregates were washed with PBS (-), incubated with Accumax (Innovative cell technologies) for 5–10 min at 37°C and dissociated into single cells by pipetting. The number of cells in the aggregates was counted using a TC20 Automated Cell Counter (Bio-Rad). The fluorescent images of days 0, 1 and 10 aggregates were captured using a BZ-X700 (KEYENCE).

### RT-PCR and real-time quantitative RT-PCR (qRT-PCR)

Total RNA was isolated using the RNeasy Mini kit (QIAGEN) according to the manufacturer’s recommendations followed by cDNA synthesis using standard protocols for ReverTra Ace (TOYOBO). qRT-PCR was performed using the QuantStudio 3 Real-Time PCR System (Thermo Fisher Scientific) and TB Green Premix Ex Taq II (Tli RNaseH Plus), ROX plus (Takara). Denaturation was performed at 95°C for 30 s, followed by 45 cycles at 95°C for 5 s and at 60°C for 34 s. Comparative Ct method quantification was used to analyze the data for the gene expression levels, and the values were normalized to those of *TBP* (housekeeping gene). The PCR reactions were performed in at least triplicate for each sample. The primer sequences are listed in S2 Table. Student’s t-tests against iPSCs with the Bonferroni correction based on the number of comparisons (genes) and zero imputation to the undetermined CT value were performed in the statistical analysis in Fig 1D. Statistical analyses in Figs 2C and 2D and 3B and 3C were performed using log10 transformed target expression values normalized to *TBP* expression values. Samples for which the CT values were not determined were removed from the analyses. One-way ANOVA and Tukey-Kramer post hoc tests were performed for multiple group comparisons.

### Immunostaining

NPC aggregates and kidney organoids were fixed with 4% PFA (Nacalai tesque)/PBS(-) overnight at 4°C. The fixed aggregates and organoids were washed with PBS(-) twice, treated with 30% sucrose (Nacalai tesque)/PBS(-) overnight at 4°C, and then frozen with OCT compound (Sakura Finetek). The frozen sections were incubated with Blocker Casein in PBS (Thermo Fisher Scientific) for 30 min at room temperature. Primary and secondary antibodies were incubated with the samples overnight at 4°C. The antibodies and lectins used in this study are listed in S3 Table.

### RNA sequencing

Total RNA was extracted using the RNeasy Mini Kit. We prepared sequencing libraries using TruSeq Stranded Total RNA (Illumina). The library was sequenced using the NovaSeq SP Reagent Kit v1.5 (200 cycles) with 101-8-8-101 cycles. Gene expression quantification was done using the analysis pipeline (2.3.4) used in the ENCODE project (https://www.encodeproject.org/pipelines/ENCPL002LPE/). GRCh38 ENSEMBL release 104 was used for the reference sequence. Gene definitions were based on GRCh38 GENCODE release 38. We performed a comparative analysis of hiPSCs and NPCs using DESeq2 [52] with the expected count calculated by the above pipeline (STAR-RSEM). Previously reported RNA-seq data were also re-analyzed (S4 Table). For NPCs [29] and hiPSCs (201B7) [30], transcript quantification was performed using Salmon [31] with Homo sapiens GRCh38 cDNA gene annotation (Ensembl). The output from Salmon was then processed using the R/Bioconductor package tximport to get gene expression values. DEG analysis was performed using iDEP (version 0.92) [53]. Enrichment (GO Biological Process) analysis for the k-means-clustered top 1,000 DEGs was performed using iDEP. We used GREIN [22] to obtain gene-level normalized counts per million (CPM) or (transcript-level) transcripts per million (TPM) from the RNA-seq data of the human fetal transcriptional atlas [21], hepatocyte-like cells [23], pancreatic progenitors [24], liver organoids [25], kidney organoids [26], endoderm lineages [54], three germ layer lineage cells [55], neuron lineages [56], cardiomyocytes [57, 58], lung lineages [59, 60], endothelial cells [58], hiPSCs [23, 24, 56–58] and hESCs [55, 58, 60]. We obtained the *CUZD1* read per million (RPKM) values of 27 types of organs and tissues from 95 human individuals [33] in the NCBI Gene database (Gene ID: 50624). The sum of the Scaled TPM (Fig 1A and 1D) or TPM (Fig 2A and 2B) plus 0.01 was subjected to log10 transformation and then used for the statistical analyses in Figs 1A and 1D and 2A and 2B. Student’s t-tests against hiPSCs with the Bonferroni correction based on the number of comparisons (genes or transcripts) were performed in the statistical analyses in Figs 1A and 1D and 2A and 2B.

### Single cell RNA sequencing

We obtained processed scRNA-seq data (count matrix) of our NPCs induced with or without activin A treatment at stage 4 from a public database (GSE146119), and alignment to the reference genome hg19, filtering, debarcoding and UMI counting was conducted using the Cell Ranger v2.1.0 pipeline (10X Genomics) [29].

We performed Seurat-based (Seurat v4.1.1) filtering using three criteria: number of detected features (nFeature_RNA) per cell, number of UMIs expressed per cell (nCount_RNA) and percent of mitochondrial gene count (percent.mt) using the following threshold parameters: nFeature_RNA (500 to 5000) and percentage of mitochondrial genes expressed (<5%) to remove multiplets (S4A Fig) [61].

We normalized our dataset using the SCTransform [62] framework with the percent of mitochondrial gene count to regress out in a second non-regularized linear regression. Then, we performed principal component (PC) analysis and determined the K-nearest neighbor graph using the first 30 PCs. The origin of the NPC dataset was visualized on a UMAP (RunUMAP, dims = 1:30). To visualize the expression of known marker genes for each cell on UMAP plots, we used the FeaturePlot function in Seurat. To visualize the expression of known markers in each NPC dataset, we used the VlnPlot function in Seurat. An analysis of correlation between the expression of hiPSC markers (*LIN28A*, *CNMD*, and *SFRP2*) and NPC markers (*SIX2* and *PAX2*) was performed using FeatureScatter function in Seurat.

### One-step RT-ddPCR

Total RNA was extracted using the RNeasy Mini Kit. One-Step RT-ddPCR was performed using a One-Step RT-ddPCR Advanced Kit for Probes (Bio-Rad), 900 nM forward and reverse primers, 250 nM probes labeled with FAM dye and ZEN/Iowa Black fluorescent quencher, and 1 mM manganese acetate solution (Bio-Rad), as previously described [14] with slight modifications. A total RNA sample (1 ng) was added to the mixture. The available sequences of primers and probes used in the present study are listed in S5 Table. Droplets were generated in 8-well cartridges using the QX200 droplet generator (Bio-Rad) or Automated Droplet Generator (Bio-Rad) according to the manufacturer’s instructions. The water-in-oil emulsions were transferred to a 96-well plate, and RT-PCR was performed using a C1000 Touch Thermal Cycler (Bio-Rad). The thermal cycling conditions were as follows: 30 min reverse transcription at 60°C, followed by 5 min enzyme activation at 95°C, and 40 cycles of a thermal profile comprising 30 s denaturation at 94°C and 60 s annealing/extension at 63°C. After the PCR amplification, the products were denatured for 10 min at 98°C and cooled at 4°C until the fluorescence intensity was measured. Fluorescence intensities of each droplet from the samples were measured using the QX200 droplet reader. Positive droplets containing amplification products were distinguished from negative droplets and counted by applying a fluorescence amplitude threshold in QuantaSoft software. The threshold was manually determined based on the distribution of the droplet and set at 4000. QuantaSoft software provides concentration results in copies of target per microliter (copies/μL). The number of copies of target per template RNA was calculated as a concentration (copies/μL) multiplied by the reaction volume (20 μL).

### RT-ddPCR

Total RNA was isolated using the RNeasy Mini Kit according to the manufacturer’s recommendations followed by cDNA synthesis using standard protocols for ReverTra Ace (TOYOBO). RT-ddPCR was performed using ddPCR™ Supermix for Probes (No dUTP, Bio-Rad), 900 nM forward and reverse primers, 250 nM probes labeled with FAM or HEX dye and ZEN-Iowa Black Fluorescent quencher according to the manufacturer’s recommendations. A cDNA sample from a total RNA sample (300–1,000 ng) was added to the mixture. The available sequences of primers and probes used in the present study are listed in S5 Table. Droplet generation, water-in-oil emulsions transfer, and RT-PCR were done following the one-step RT-ddPCR procedure [14]. The thermal cycling conditions were as follows: 10 min enzyme activation at 95°C and 40 cycles of a thermal profile comprising 30 s denaturation at 94°C and 60 s annealing/extension at 57.1°C. After the PCR amplification, the products were denatured for 10 min at 98°C and cooled at 4°C until the fluorescence intensity was measured. The fluorescence intensities of each droplet from the samples were measured using the QX200 droplet reader. Positive droplets containing amplification products were distinguished from negative droplets and counted by applying a fluorescence amplitude threshold in QuantaSoft software. The threshold was manually determined based on the distribution of the droplet and set at 4000 for *TBP* and 7000 for *MIR302CHG*. QuantaSoft software provides the estimated concentration results in copies of target per microliter (copies/μL). The ratio of the copy number of target genes per copy number of *TBP* was calculated.

The sum of the *TBP*-normalized copy number plus the minimum *TBP*-normalized copy number of the dataset were subjected to log10 transformation and then used for the statistical analyses in Fig 4C–4E. One-way ANOVA and Tukey-Kramer post hoc tests were performed for multiple group comparisons.

### Magnetic bead-based enrichment

Magnetic bead-based enrichment was performed bv MACS (Miltenyi Biotec). hiPSCs and NPCs were dissociated using Accumax (Innovative Cell Technologies, Inc.), and the cell suspension was spun down at 200g for 7 min at 4°C. After centrifugation, the supernatant was discarded, and the cell pellets were reconstituted in MACS rinsing solution with 0.5% BSA (Wako or Miltenyi Biotec) and 10 μM Y-27632. Cell suspensions containing hiPSCs and NPCs (1:10^6^) were prepared and incubated with 1 μL anti-human TRA-1-60 microbeads (Miltenyi Biotec) per 10^6^ cells for 15 min at 4°C. Tapping was performed every five minutes. The suspensions were then filtered using a 100-μm pore cell strainer (Falcon) and applied to an LS column attached to a MidiMACS separator (Miltenyi Biotec). The column was then washed twice using 3 mL of MACS rinsing solution with 0.5% BSA. In the process of applying the labeled cells and washing, the syringe was gently pressed to let the drops fall at about one drop per second. The positive fractions were flushed out and collected by gently pressing with a syringe at a rate of 2–3 drops per second. After centrifugation at 200g for 5 min, the cells from both the positive and negative fractions were dissolved using buffer RLT (QIAGEN) with β-mercaptoethanol and stored at -80°C until the analysis. Total RNA was isolated using the RNeasy Mini Kit with DNase I treatment according to the manufacturer’s instructions. The RNA quantity was determined using NanoDrop (Thermo Fisher Scientific). The RNA samples of the TRA-1-60 positive fraction (12.9 μL) and flow-through fractions (up to 1,000 ng of RNA) were used in a total of 20 μL RT reaction mix, and cDNA synthesis was performed according to standard protocols for ReverTra Ace (TOYOBO).

### Flow cytometry and cell sorting

We prepared cell samples by incubation with Accumax (Innovative Cell Technologies) for 7 min at 37°C and dissociation by pipetting or using each fraction of the MACS separation. Dead cells stained with 4’,6-diamidino-2-phenylindole, dihydrochloride (DAPI; 0.1 ng/mL; Thermo Fisher Scientific) were excluded from the analyses. The cells were analyzed and sorted using a FACS Aria II cell sorter (BD), and the data were analyzed using the FACS Diva (BD) software program.

In S4 Fig, we manually prepared and analyzed a 1:10,000 mixture of hiPSCs constitutively expressing tdTomato and induced NPCs from a non-fluorescent hiPSC line (ff-I 14s04) by flow cytometry and examined whether the manual mixing was done accurately. The tdTomato(+) fractions sorted by flow cytometry were analyzed again to test whether the positive fractions were not noise such as bubbles.

In Fig 3, we used the mixtures of hiPSCs constitutively expressing EGFP (317–12 cells) and NPCs derived from a non-fluorescent hiPSC line (QHJI 14s04) and evaluated the degree of magnetic bead-based enrichment in the positive and negative fractions by flow cytometry using GFP as an indicator.

### Generation of an hiPSC line constitutively expressing tdTomato

The hiPSC line constitutively expressing tdTomato was generated as described previously with some modifications [63, 64]. Briefly, the EGFP sequence between the Nco1 and EcoR1 sites was replaced with a PCR-amplified tdTomato fragment (see the primer sequences in S6 Table) in the *piggyBac* transposon vector pPV-EF1a-EiP-A (provided by Prof. Akitsu Hotta) to create pPV-EF1a-tdTomato-A, allowing us to ubiquitously express tdTomato under the human EF1a promoter. pPV-EF1a-tdTomato-A plasmids were electroporated into 585A1 hiPSCs [50] with the *piggyBac* transposase-expressing vector pHL-EF1a-hcPBase-A [63] using a NEPA21 electroporator (Nepa Gene). Highly tdTomato-expressing hiPSCs were FACS-sorted after a 5-day culture and isolated for single cell expansion. The isolated colony with the highest tdTomato expression was used in this study.

## Supporting information

S1 Fig Analysis of *LIN28A*, *ESRG*, *CNMD* and *SFRP2* expression in various human fetal tissues and hiPSC-derived progenitor cells and organoids using publicly available RNA sequencing datasets. (A) Scatter plots of the CPM values of *LIN28A*, *ESRG*, *CNMD* and *SFRP2* in the various human fetal tissues from first or second trimesters reported in Roost et al. (2015). (B-E) Scatter plots of the CPM values of *LIN28A*, *ESRG*, *CNMD* and *SFRP2* in hiPSCs, day 12 hiPSC-derived hepatoblasts, and day 20 hiPSC-derived hepatocytes with or without methoxamine treatment reported in Kotaka et al. (2017) (B), hiPSCs or hiPSC-derived pancreatic progenitors reported in Kimura et al. (2020) (C), day 6 hiPSC-derived foregut spheroids, days 20 and 25 hiPSC-derived liver organoids reported in Ouchi et al. (2019) (D), and hiPSC-derived kidney progenitors (day 0–3 pellets) and organoids (day 11–18 pellets) reported in Takasato et al. (2020) (E).
(TIF)

S2 Fig Microarray analysis of *Lin28a*, *Cnmd* and *Sfrp2* in mouse metanephric mesenchyme. (A-C) Microarray analysis of *Lin28a* (A), *Cnmd* (B) and *Sfrp2* (C) in mouse developing kidneys using GUDMAP.
(TIF)

S3 Fig Differential gene expression analysis of hiPSCs and hiPSC-derived OSR1(+)SIX2(+)NPCs and induction of NPCs and kidney organoids from a clinical-grade iPSC line. (A) Heatmap of the top 1,000 DEGs between hiPSCs and hiPSC-derived OSR1(+)SIX2(+)NPCs and the results of the enrichment analysis of three gene clusters defined by the gene expression patterns. The blue, yellow and purple bands on the left of the heatmap and the table correspond to each of the three clusters. (B) Representative immunofluorescence images of an induced NPC aggregate for NPC markers, SIX2 and PAX2. Scale bar, 500 μm. (C) Representative bright field and immunofluorescence images of kidney organoids derived from induced NPCs for markers of renal lineage cells (PAX8), glomeruli (PODX) and renal tubules (LTL and CDH1). Scale bars, 500 μm. (D) q RT-PCR analysis of the expression of *LIN28A* and markers for hiPSCs (*POU5F1* and *NANOG*) and NPCs (*OSR1* and *SIX2*). Each value was normalized to that of hiPSCs.
(TIF)

S4 Fig scRNA-seq analysis of purified OSR1(+)SIX2(+) induced NPCs for iPSC and NPC marker genes. (A) Violin plots of the cells in hiPSC-derived metanephric and mesonephric NPC populations reported in Tsujimoto et al. (2020) with standard quality control parameters after filtering. (B) UMAP plots for hiPSC-derived metanephric and mesonephric NPCs. The number of cells in each NPC population: metanephric NPCs, 190; and mesonephric NPCs, 199. (C, D) Violin plots of representative NPC (C) and iPSC (D) markers of hiPSC-derived metanephric and mesonephric NPCs. (E) UMAP plots of representative NPC markers (*SIX2*, *PAX2*, *PAX8* and *WT1*) in hiPSC-derived metanephric and mesonephric NPCs. (F) Scatter plots of iPSC markers (*LIN28A*, *CNMD* and *SFRP2*) and NPC markers (*SIX2* and *PAX2*). Numbers above the plots are Pearson correlation coefficients. Mesonephric NPCs were induced from hiPSCs by the same protocol as the metanephric NPC induction except for removing activin A at Stage 4. MESO, mesonephric NPC; META, metanephric NPC.
(TIF)

S5 Fig Digital droplet PCR assays of hiPSCs intermingled in induced NPCs using *CUZD1*. (A) Flow cytometric analysis of a 1:10,000 mixture of hiPSCs constitutively expressing tdTomato and induced NPCs from a clinical-grade hiPSC line (left and center panels) indicates that the manual mixing was done accurately. A histogram (right panel) of the tdTomato(+) fractions that were sorted and FACS analyzed again suggests that the positive fractions were not noise such as bubbles. (B) A box plot for the estimated number of *CUZD1* copy/total RNA (1 ng) of hiPSCs (iPSC), 1:10,000 mixture of hiPSCs and induced NPCs (0.01%iPSC), and NPCs (NPC) using a one-step ddPCR assay suggests that the estimation is biased for some technical reasons. (C) Representative scatter plots of positive and negative droplets for *CUZD1* and *TBP* using a RT-ddPCR of iPSC, 0.01%iPSC, and NPC (left panels) and a box plot for the *TBP*-normalized estimated number of *CUZD1* copies according to an RT-ddPCR (right panel). (D, E) A ddPCR analysis of *CUZD1* using an hiPSC cDNA dilution series sample diluted by the cDNAs of NPCs (D) or day 4 cells (E) shows some concentration-dependent changes in the *TBP*-normalized copy number of *CUZD1*.
(TIF)

S6 Fig Marker gene expressions in hiPSCs, primitive streak and NPCs at each stage of the NPC induction protocol. Scatter plots of *TBP*-normalized expression values by qRT-PCR for marker genes of hiPSCs (*MIR302CHG*, *CUZD1*, and *POU5F1*), primitive streak (*TBX6*) and NPCs (*OSR1* and *SIX2*). UD: number of samples with undetermined CT values.
(TIF)

S7 Fig Identification of a lncRNA, *MIR302CHG*, as a novel detection marker for hiPSCs intermingled in induced NPCs. (A) A scatter plot of the RPKM values of *CUZD1* in 27 tissue samples from 95 human individuals reported by Fagerberg et al. (2014). (B) A volcano plot of the DEGs at log10(FDR) > 5 and log2(fold change) < −10 (blue dots) or > 10 (red dots) and a list of 26 candidate hiPSC markers. A gene classification by IPA is shown. (C) Scatter plots of *TBP*-normalized expression values of several hiPSC lines normalized to the QHJI 14s04 hiPSC line by qRT-PCR for marker genes of hiPSCs (*MIR302CHG*, *CUZD1* and *POU5F1*). (D) A scatter plot of *TBP*-normalized qRT-PCR dCT values of hiPSCs (iPSC) and induced NPCs (NPC) for 15 *MIR302CHG* primers. Primers #1-#5 are specific for both ENST00000509938.1 and ENST00000505215.1, while primers #6-#15 are specific for ENST00000509938.1. Primer #2 was used for the other qRT-PCR assays. UD: undetermined CT values.
(TIF)

S8 Fig *MIR302CHG* expression in hiPSC-derived pancreatic progenitor cells, hESC-derived endoderm lineages, and hiPSC-derived hepatocyte and liver organoids from publicly available RNA-seq datasets. (A-D) Scatter plots of the TPM values of each transcript variant of *MIR302CHG* in hiPSCs and hiPSC-derived pancreatic progenitors reported in Kimura et al. (2020) (A), hESC-derived endoderm lineages reported in Loh et al. (2013) (B), hiPSCs, hiPSC-derived D12 hepatoblasts and D20 hepatocytes reported in Kotaka et al. (2017) (C), and hiPSC-derived day 6 foregut spheroids and days 20 and 25 hiPSC-derived liver organoids reported in Ouchi et al. (2019) (D).
(TIF)

S9 Fig *MIR302CHG* expression in hESC-derived three germ layer lineages, and hiPSC-derived neuron, cardiomyocyte, and lung lineages from publicly available RNA-seq datasets. (A-D) Scatter plots of the TPM values of each transcript variant of *MIR302CHG* in hESC-derived definitive endoderm, splanchnic mesoderm, neural progenitor cells and pre-neural crest cells reported in Cliff et al. (2017) (A), hiPSCs and hiPSC-derived neural progenitor cells and neurons reported in Chen et al. (2013) (B), hiPSCs from 58 Yoruba individuals and hiPSC-derived cardiomyocytes reported in Banovich et al. (2018) (C), hiPSC-derived D5 definitive endoderm, D10 anterior foregut, D15 and D20 lung progenitors and D52 and D66 lung epithelial cells reported in Kerschner et al. (2020) (D), and hESCs, hESC-derived D15 lung progenitors, D35 SFTPC(+) and (-) cells of lung organoids, and GW21 human fetal lung cells (E).
(TIF)

S10 Fig *MIR302CHG* expression in hiPSC- and hESC-derived cardiomyocytes and endothelial cells from publicly available RNA-seq datasets. Scatter plots of the TPM values of each transcript variant of *MIR302CHG* in hiPSCs, in vitro fertilization embryo-derived hESCs, somatic cell nuclear transfer-derived hESCs, and their derivative cardiomyocytes and endothelial cells reported in Zhao et al. (2017).
(TIF)

S11 Fig NPC growth assay using iPSC media. (A) The cell number of each cell aggregate treated with Essential 8 or AK03N media at 1, 3, 5, 8 and 10 days after seeding. (B, C) Representative brightfield or fluorescent images of OSR1 (GFP) SIX2 (tdTomato) from days 0 (B), 1 and 10 (C) cell aggregates treated with Essential 8 or AK03N media. **p <0.01 by paired Student’s t-tests comparing the cell number in day 1 (D1) and day 10 (D10) cell aggregates. Data are represented as the mean ± SEM (n = 12). Scale bars, 300 μm.
(TIF)

S12 Fig The combination assay of magnetic bead-based cell isolation and qRT-PCR analysis for NPCs. Scatter plots of the *TBP*-normalized gene expressions by qRT-PCR for *MIR302CHG*, *CUZD1*, and *POU5F1* in NPCs, and MACS positive or negative fraction flowthroughs of the NPCs (PF or NF). The dots and lines in the center of the scatter plots indicate the experimental data and mean values of the data, respectively. UD: number of samples with undetermined CT values.
(TIF)

S1 Table Highly expressed genetic markers (log2FC > 10 & FDR <10^−5^) in hiPSCs compared to day 4 cells from the NPC differentiation protocol. (DOCX)

S2 Table Primer sequences for qRT-PCR used in this study. (DOCX)

S3 Table Antibodies and lectin used in this study. (DOCX)

S4 Table RNA sequencing data used in this study. (DOCX)

S5 Table Primer and probe sequences for ddPCR used in this study. (DOCX)

S6 Table Recombinant DNA used in this study. (DOCX)

S1 Data Adjusted *p*-values by Tukey-Kramer post hoc tests. (XLSX)

S1 Dataset Transcript count data. (XLSX)

S1 File Informed consent forms. (ZIP)
