# Supplementary figures and images for: In vitro methods to ensure absence of residual undifferentiated human induced pluripotent stem cells intermingled in induced nephron progenitor cells
Source: PLoS One. 2022 Nov 15;17(11):e0275600. doi: 10.1371/journal.pone.0275600 (PMC9665373; doi:10.1371/journal.pone.0275600)

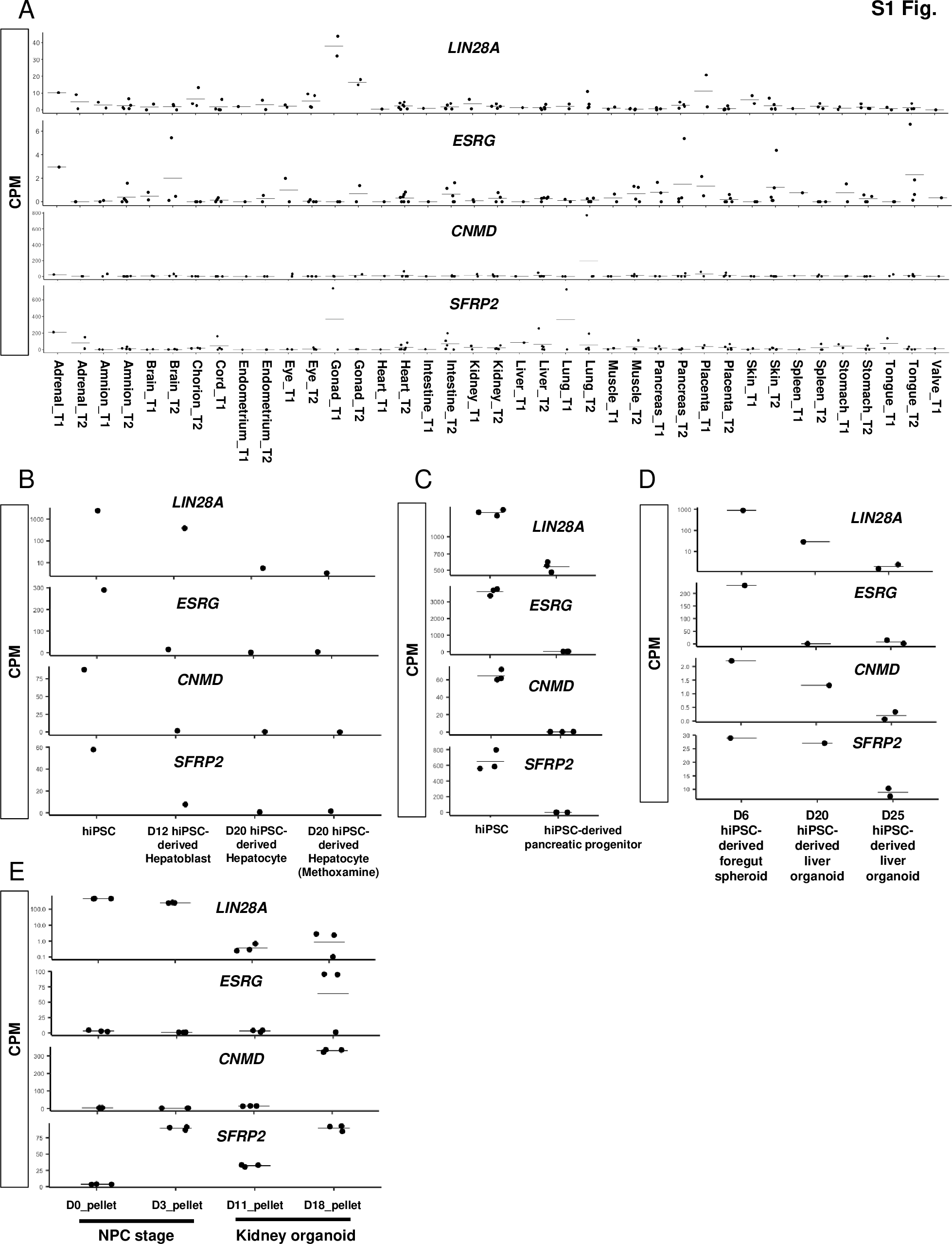

Supplement: S1 Fig — (A) Scatter plots of the CPM values of LIN28A, ESRG, CNMD and SFRP2 in the various human fetal tissues from first or second trimesters reported in Roost et al. (2015). (B-E) Scatter plots of the CPM values of LIN28A, ESRG, CNMD and SFRP2 in hiPSCs, day 12 hiPSC-derived hepatoblasts, and day 20 hiPSC-derived hepatocytes with or without methoxamine treatment reported in Kotaka et al. (2017) (B), hiPSCs or hiPSC-derived pancreatic progenitors reported in Kimura et al. (2020) (C), day 6 hiPSC-derived foregut spheroids, days 20 and 25 hiPSC-derived liver organoids reported in Ouchi et al. (2019) (D), and hiPSC-derived kidney progenitors (day 0–3 pellets) and organoids (day 11–18 pellets) reported in Takasato et al. (2020) (E). (TIF) [file pone.0275600.s001.tif]

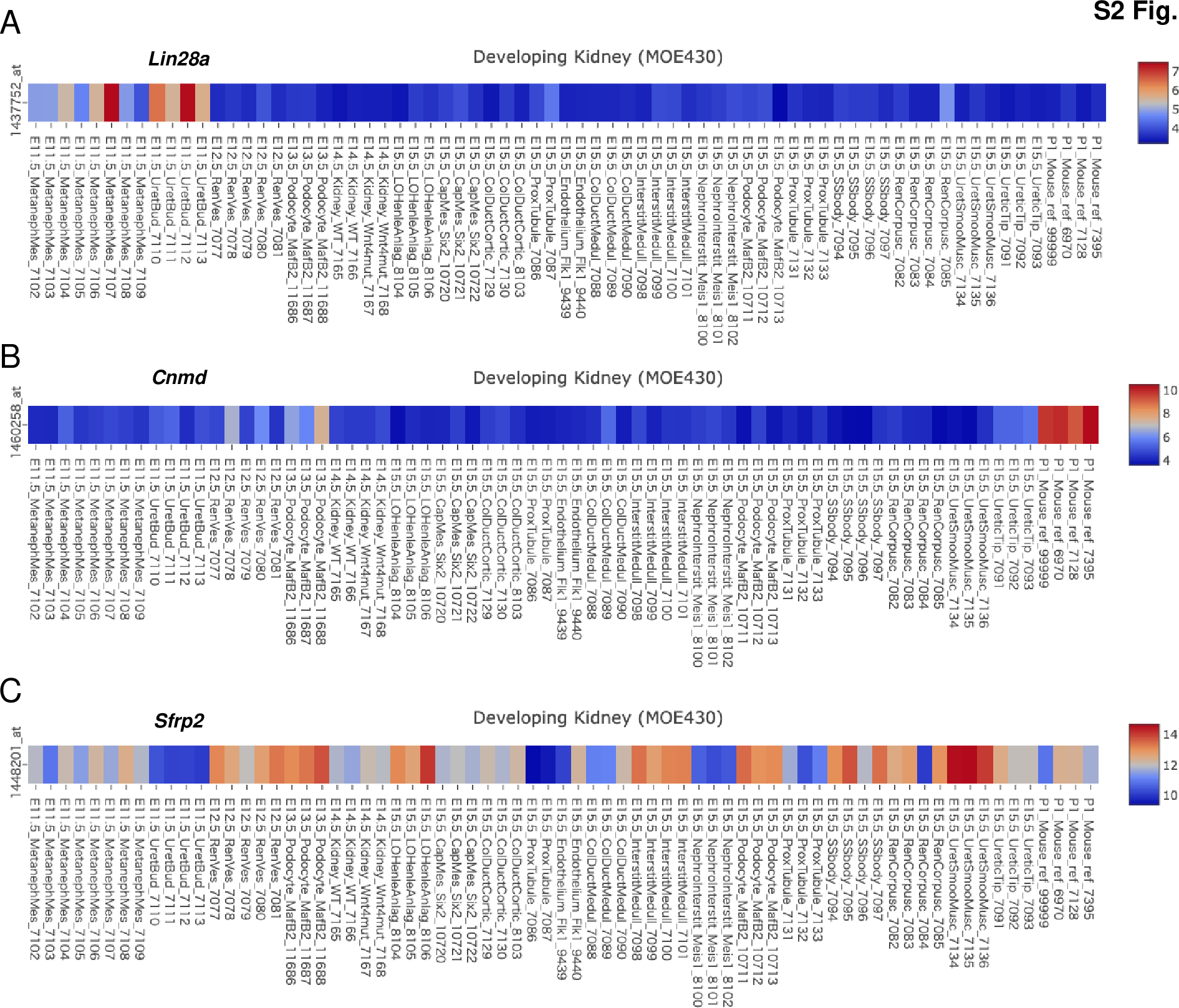

Supplement: S2 Fig — (A-C) Microarray analysis of Lin28a (A), Cnmd (B) and Sfrp2 (C) in mouse developing kidneys using GUDMAP. (TIF) [file pone.0275600.s002.tif]

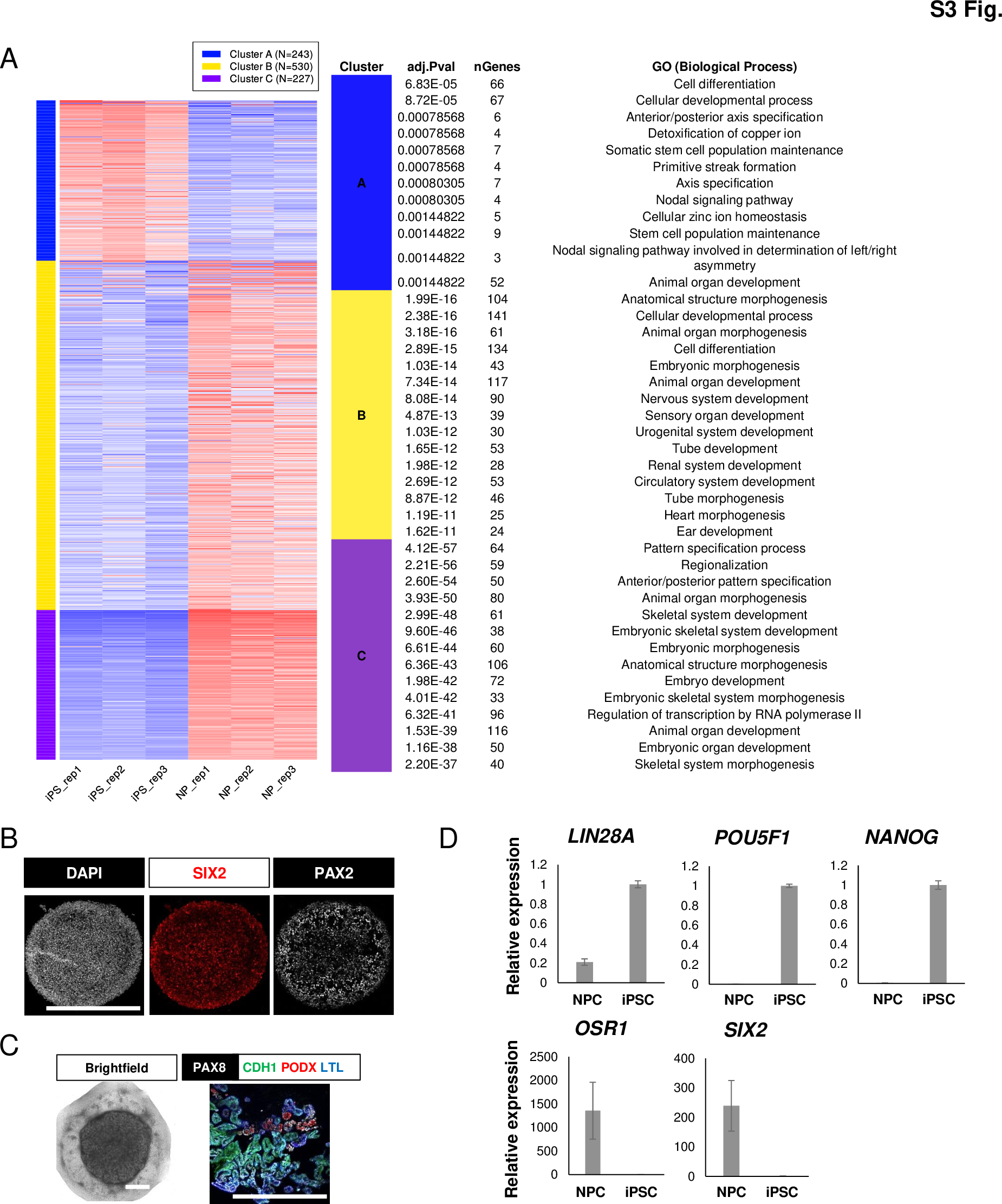

Supplement: S3 Fig — (A) Heatmap of the top 1,000 DEGs between hiPSCs and hiPSC-derived OSR1(+)SIX2(+)NPCs and the results of the enrichment analysis of three gene clusters defined by the gene expression patterns. The blue, yellow and purple bands on the left of the heatmap and the table correspond to each of the three clusters. (B) Representative immunofluorescence images of an induced NPC aggregate for NPC markers, SIX2 and PAX2. Scale bar, 500 μm. (C) Representative bright field and immunofluorescence images of kidney organoids derived from induced NPCs for markers of renal lineage cells (PAX8), glomeruli (PODX) and renal tubules (LTL and CDH1). Scale bars, 500 μm. (D) q RT-PCR analysis of the expression of LIN28A and markers for hiPSCs (POU5F1 and NANOG) and NPCs (OSR1 and SIX2). Each value was normalized to that of hiPSCs. (TIF) [file pone.0275600.s003.tif]

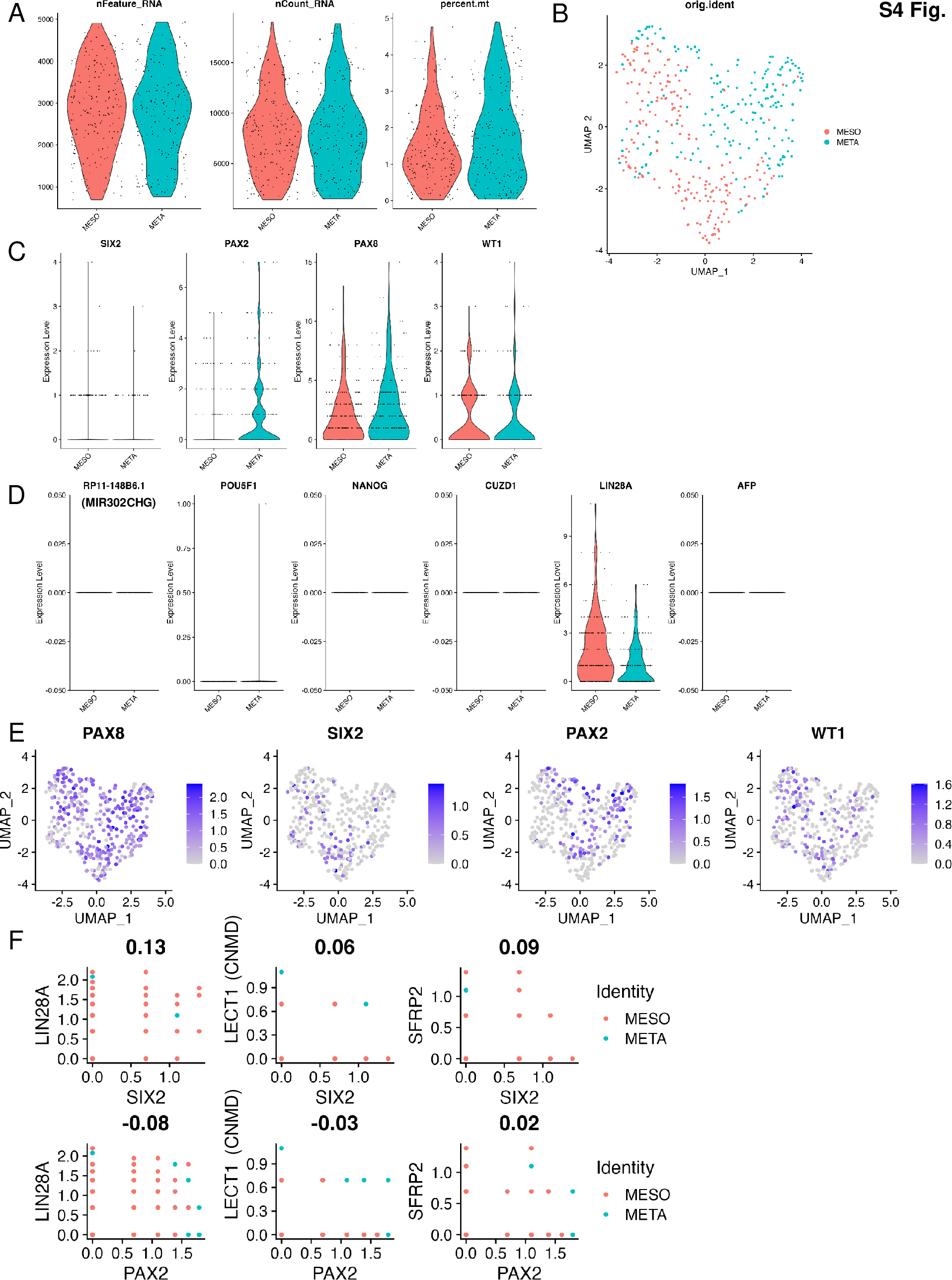

Supplement: S4 Fig — (A) Violin plots of the cells in hiPSC-derived metanephric and mesonephric NPC populations reported in Tsujimoto et al. (2020) with standard quality control parameters after filtering. (B) UMAP plots for hiPSC-derived metanephric and mesonephric NPCs. The number of cells in each NPC population: metanephric NPCs, 190; and mesonephric NPCs, 199. (C, D) Violin plots of representative NPC (C) and iPSC (D) markers of hiPSC-derived metanephric and mesonephric NPCs. (E) UMAP plots of representative NPC markers (SIX2, PAX2, PAX8 and WT1) in hiPSC-derived metanephric and mesonephric NPCs. (F) Scatter plots of iPSC markers (LIN28A, CNMD and SFRP2) and NPC markers (SIX2 and PAX2). Numbers above the plots are Pearson correlation coefficients. Mesonephric NPCs were induced from hiPSCs by the same protocol as the metanephric NPC induction except for removing activin A at Stage 4. MESO, mesonephric NPC; META, metanephric NPC. (TIF) [file pone.0275600.s004.tif]

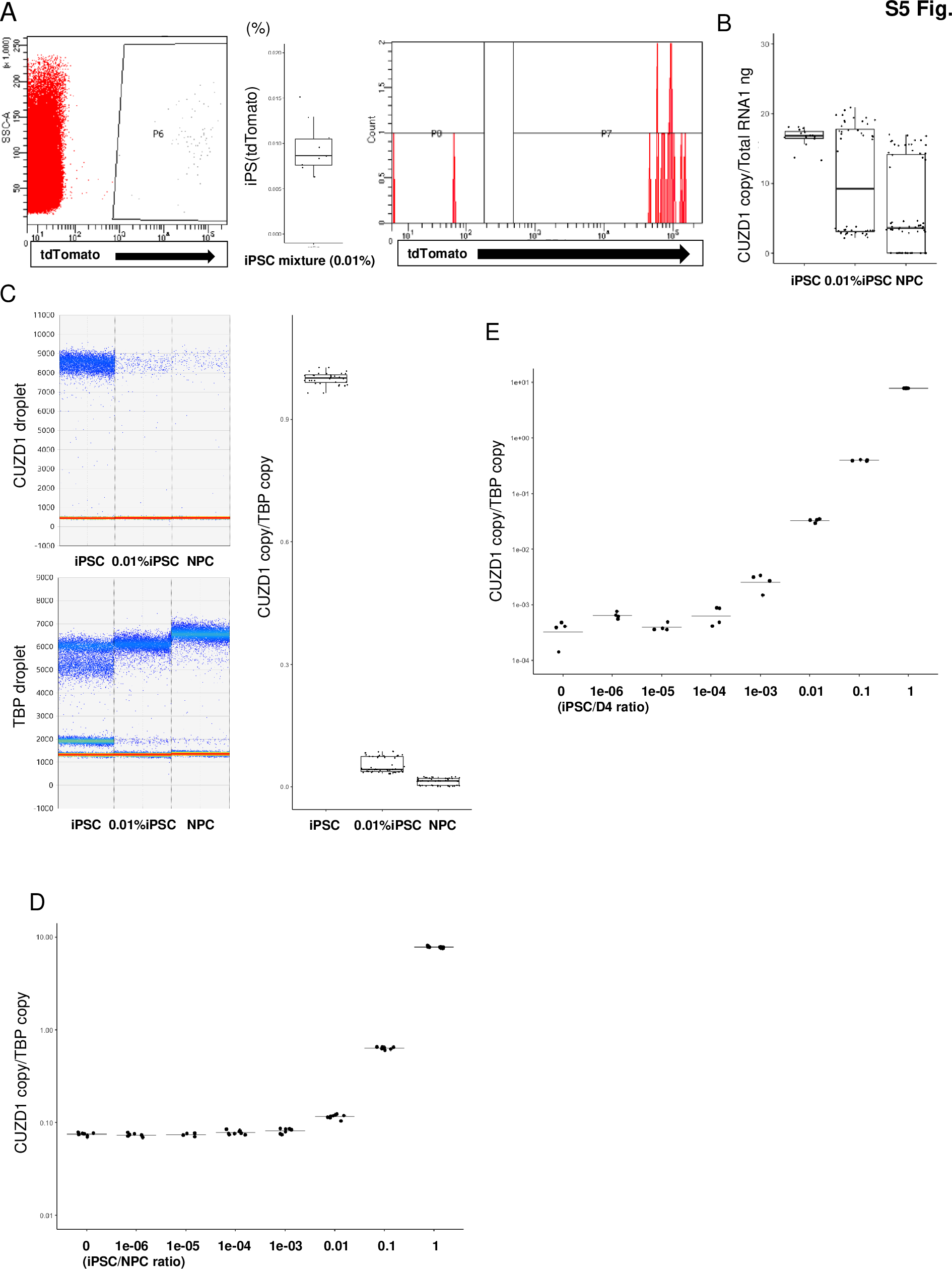

Supplement: S5 Fig — (A) Flow cytometric analysis of a 1:10,000 mixture of hiPSCs constitutively expressing tdTomato and induced NPCs from a clinical-grade hiPSC line (left and center panels) indicates that the manual mixing was done accurately. A histogram (right panel) of the tdTomato(+) fractions that were sorted and FACS analyzed again suggests that the positive fractions were not noise such as bubbles. (B) A box plot for the estimated number of CUZD1 copy/total RNA (1 ng) of hiPSCs (iPSC), 1:10,000 mixture of hiPSCs and induced NPCs (0.01%iPSC), and NPCs (NPC) using a one-step ddPCR assay suggests that the estimation is biased for some technical reasons. (C) Representative scatter plots of positive and negative droplets for CUZD1 and TBP using a RT-ddPCR of iPSC, 0.01%iPSC, and NPC (left panels) and a box plot for the TBP-normalized estimated number of CUZD1 copies according to an RT-ddPCR (right panel). (D, E) A ddPCR analysis of CUZD1 using an hiPSC cDNA dilution series sample diluted by the cDNAs of NPCs (D) or day 4 cells (E) shows some concentration-dependent changes in the TBP-normalized copy number of CUZD1. (TIF) [file pone.0275600.s005.tif]

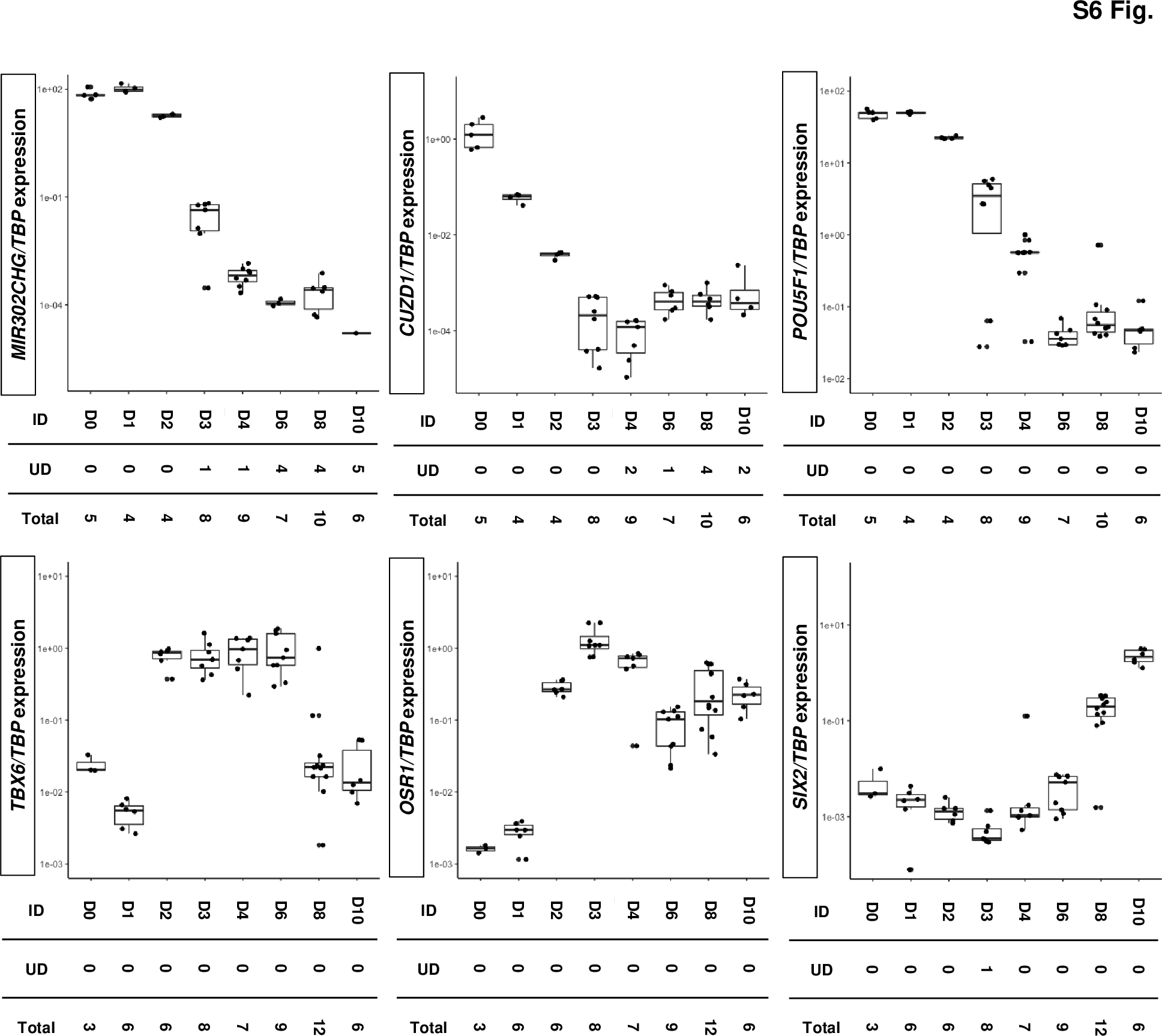

Supplement: S6 Fig — Scatter plots of TBP-normalized expression values by qRT-PCR for marker genes of hiPSCs (MIR302CHG, CUZD1, and POU5F1), primitive streak (TBX6) and NPCs (OSR1 and SIX2). UD: number of samples with undetermined CT values. (TIF) [file pone.0275600.s006.tif]

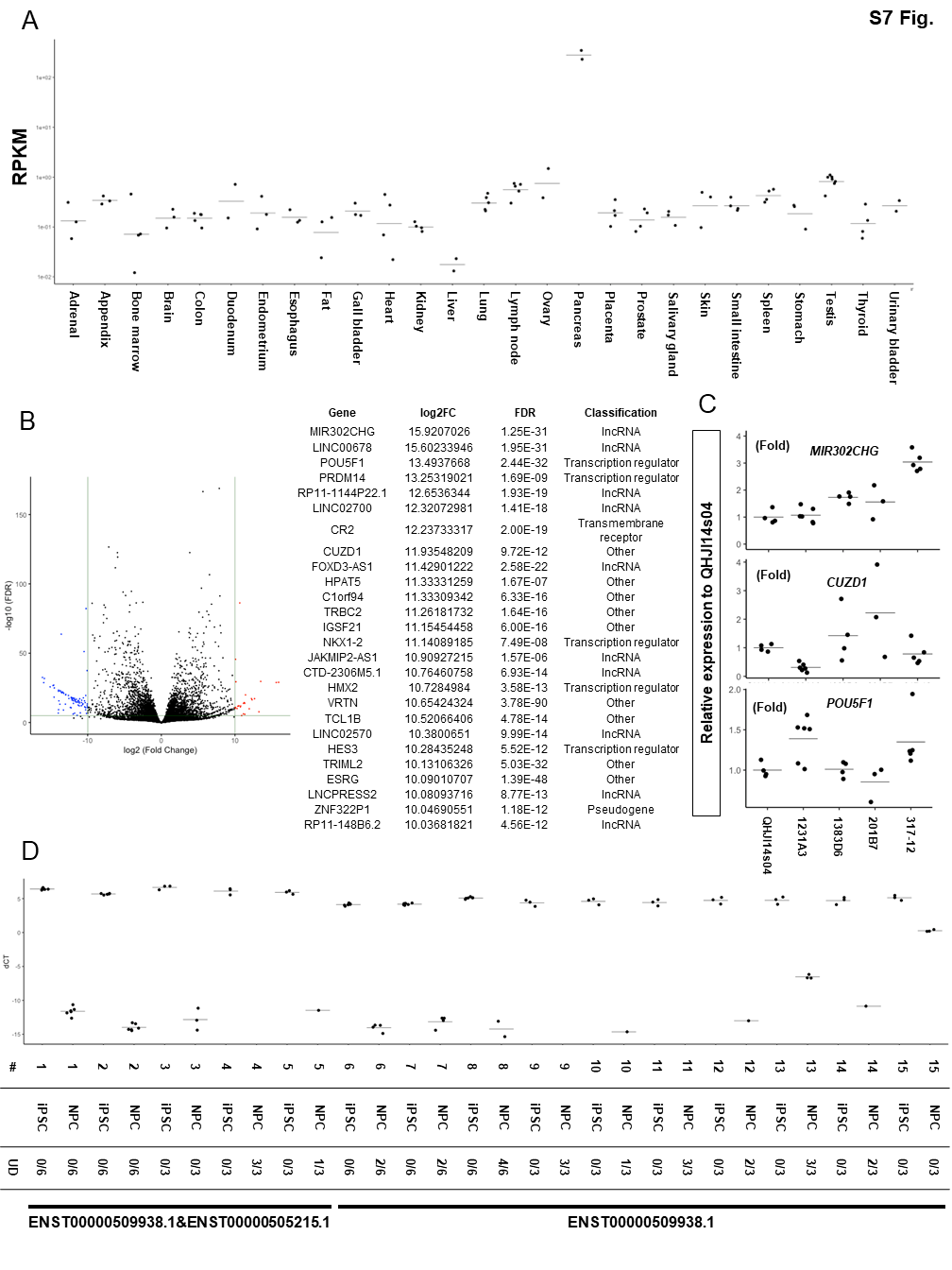

Supplement: S7 Fig — (A) A scatter plot of the RPKM values of CUZD1 in 27 tissue samples from 95 human individuals reported by Fagerberg et al. (2014). (B) A volcano plot of the DEGs at log10(FDR) > 5 and log2(fold change) < −10 (blue dots) or > 10 (red dots) and a list of 26 candidate hiPSC markers. A gene classification by IPA is shown. (C) Scatter plots of TBP-normalized expression values of several hiPSC lines normalized to the QHJI 14s04 hiPSC line by qRT-PCR for marker genes of hiPSCs (MIR302CHG, CUZD1 and POU5F1). (D) A scatter plot of TBP-normalized qRT-PCR dCT values of hiPSCs (iPSC) and induced NPCs (NPC) for 15 MIR302CHG primers. Primers #1-#5 are specific for both ENST00000509938.1 and ENST00000505215.1, while primers #6-#15 are specific for ENST00000509938.1. Primer #2 was used for the other qRT-PCR assays. UD: undetermined CT values. (TIF) [file pone.0275600.s007.tif]

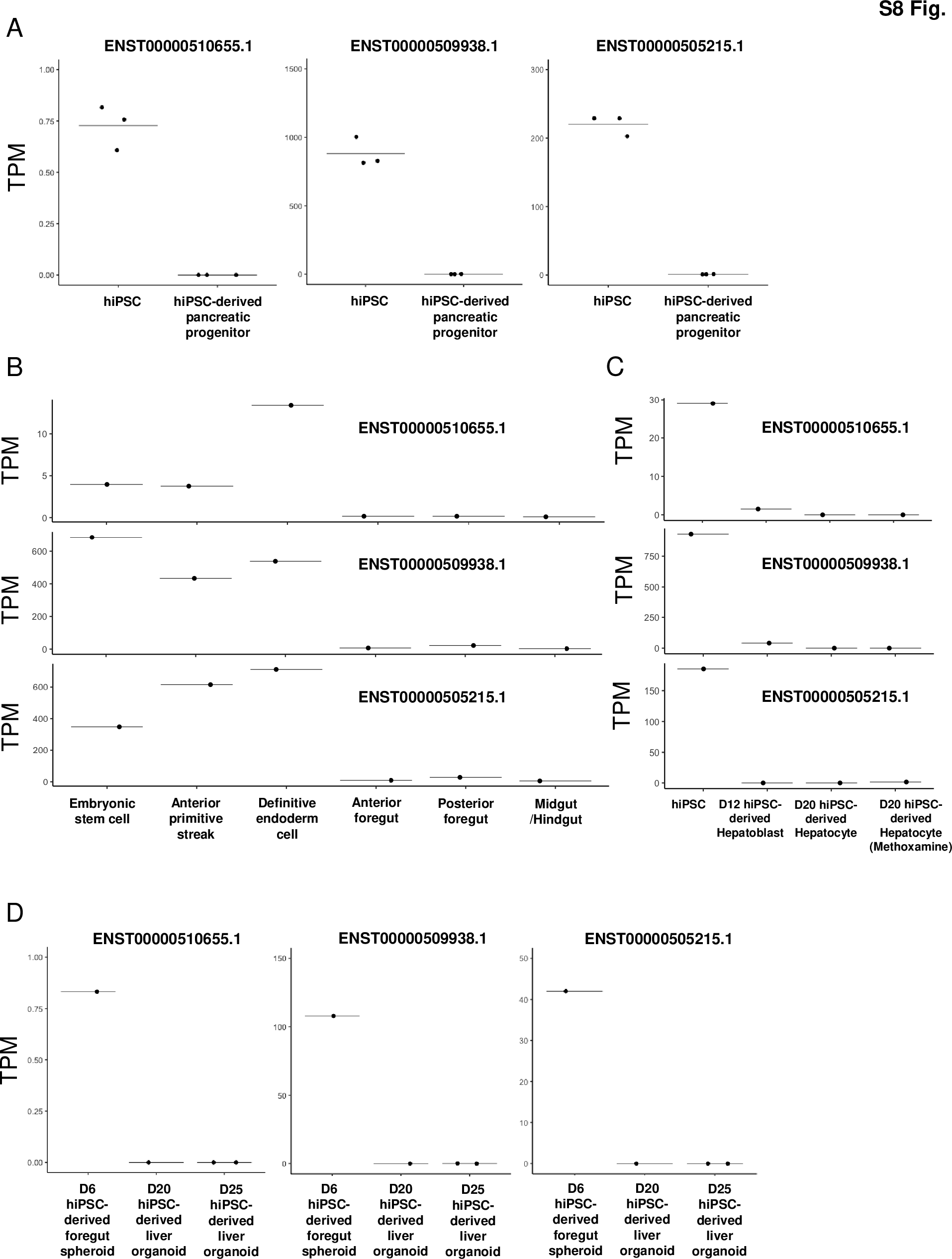

Supplement: S8 Fig — (A-D) Scatter plots of the TPM values of each transcript variant of MIR302CHG in hiPSCs and hiPSC-derived pancreatic progenitors reported in Kimura et al. (2020) (A), hESC-derived endoderm lineages reported in Loh et al. (2013) (B), hiPSCs, hiPSC-derived D12 hepatoblasts and D20 hepatocytes reported in Kotaka et al. (2017) (C), and hiPSC-derived day 6 foregut spheroids and days 20 and 25 hiPSC-derived liver organoids reported in Ouchi et al. (2019) (D). (TIF) [file pone.0275600.s008.tif]

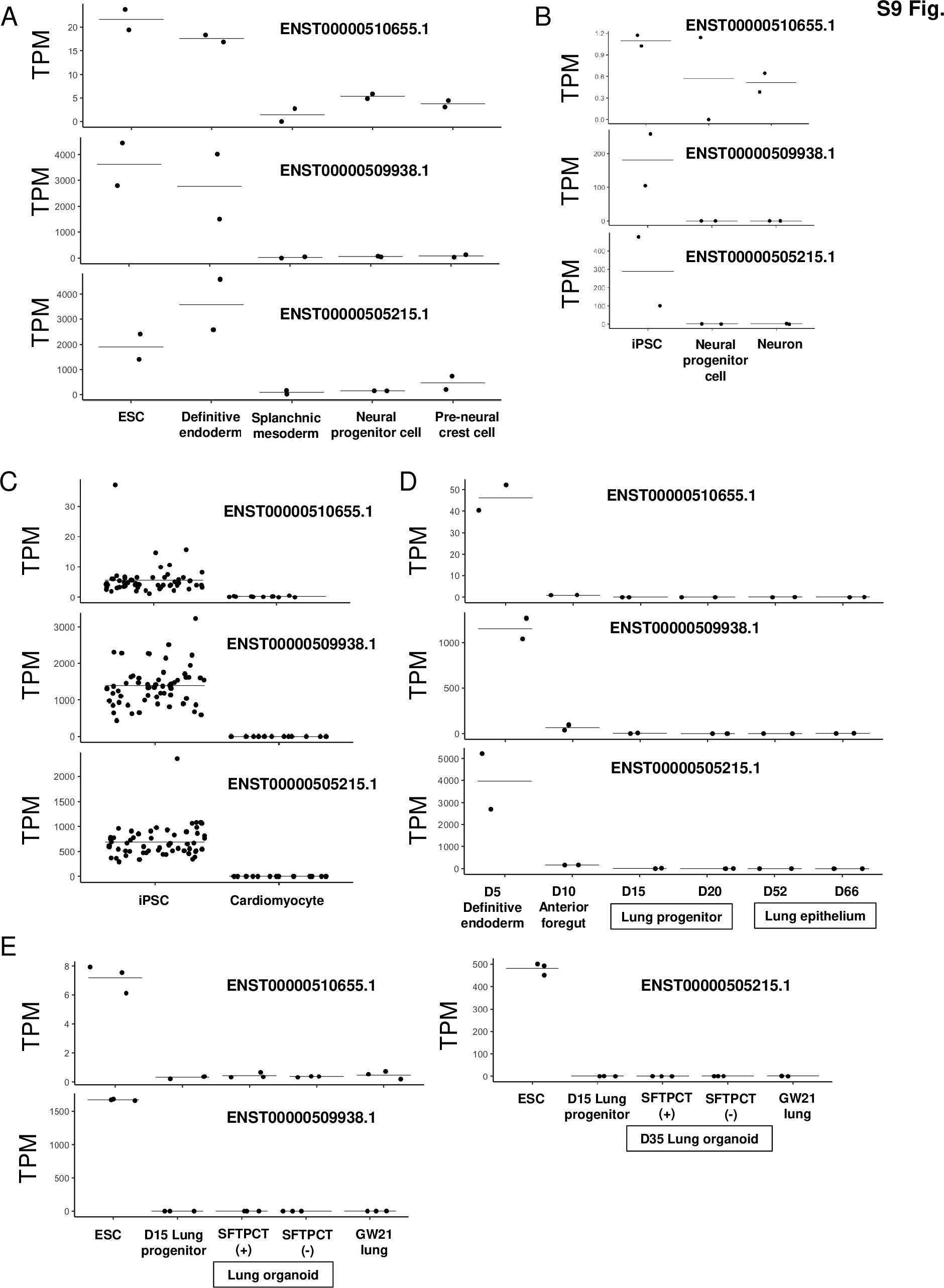

Supplement: S9 Fig — (A-D) Scatter plots of the TPM values of each transcript variant of MIR302CHG in hESC-derived definitive endoderm, splanchnic mesoderm, neural progenitor cells and pre-neural crest cells reported in Cliff et al. (2017) (A), hiPSCs and hiPSC-derived neural progenitor cells and neurons reported in Chen et al. (2013) (B), hiPSCs from 58 Yoruba individuals and hiPSC-derived cardiomyocytes reported in Banovich et al. (2018) (C), hiPSC-derived D5 definitive endoderm, D10 anterior foregut, D15 and D20 lung progenitors and D52 and D66 lung epithelial cells reported in Kerschner et al. (2020) (D), and hESCs, hESC-derived D15 lung progenitors, D35 SFTPC(+) and (-) cells of lung organoids, and GW21 human fetal lung cells (E). (TIF) [file pone.0275600.s009.tif]

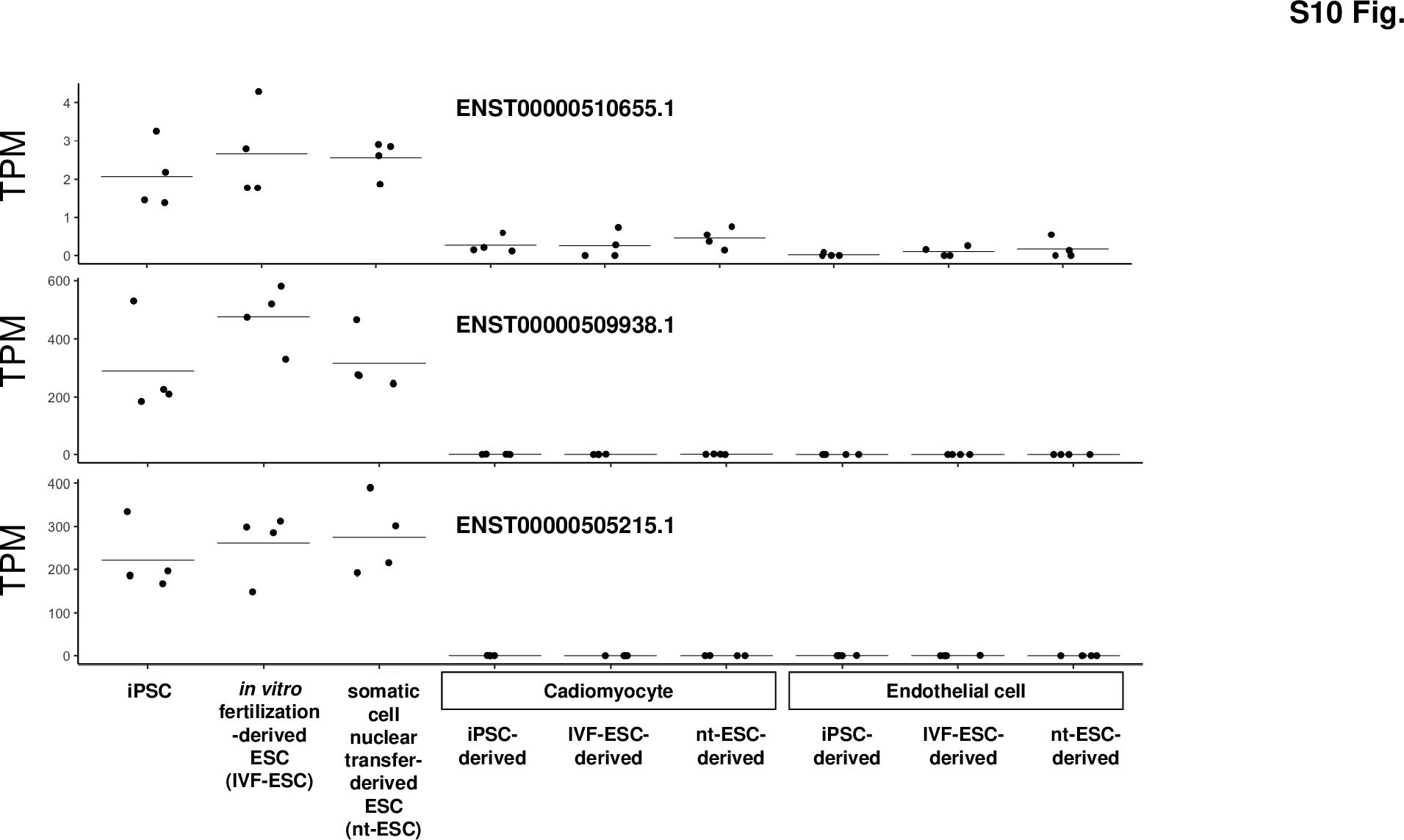

Supplement: S10 Fig — Scatter plots of the TPM values of each transcript variant of MIR302CHG in hiPSCs, in vitro fertilization embryo-derived hESCs, somatic cell nuclear transfer-derived hESCs, and their derivative cardiomyocytes and endothelial cells reported in Zhao et al. (2017). (TIF) [file pone.0275600.s010.tif]

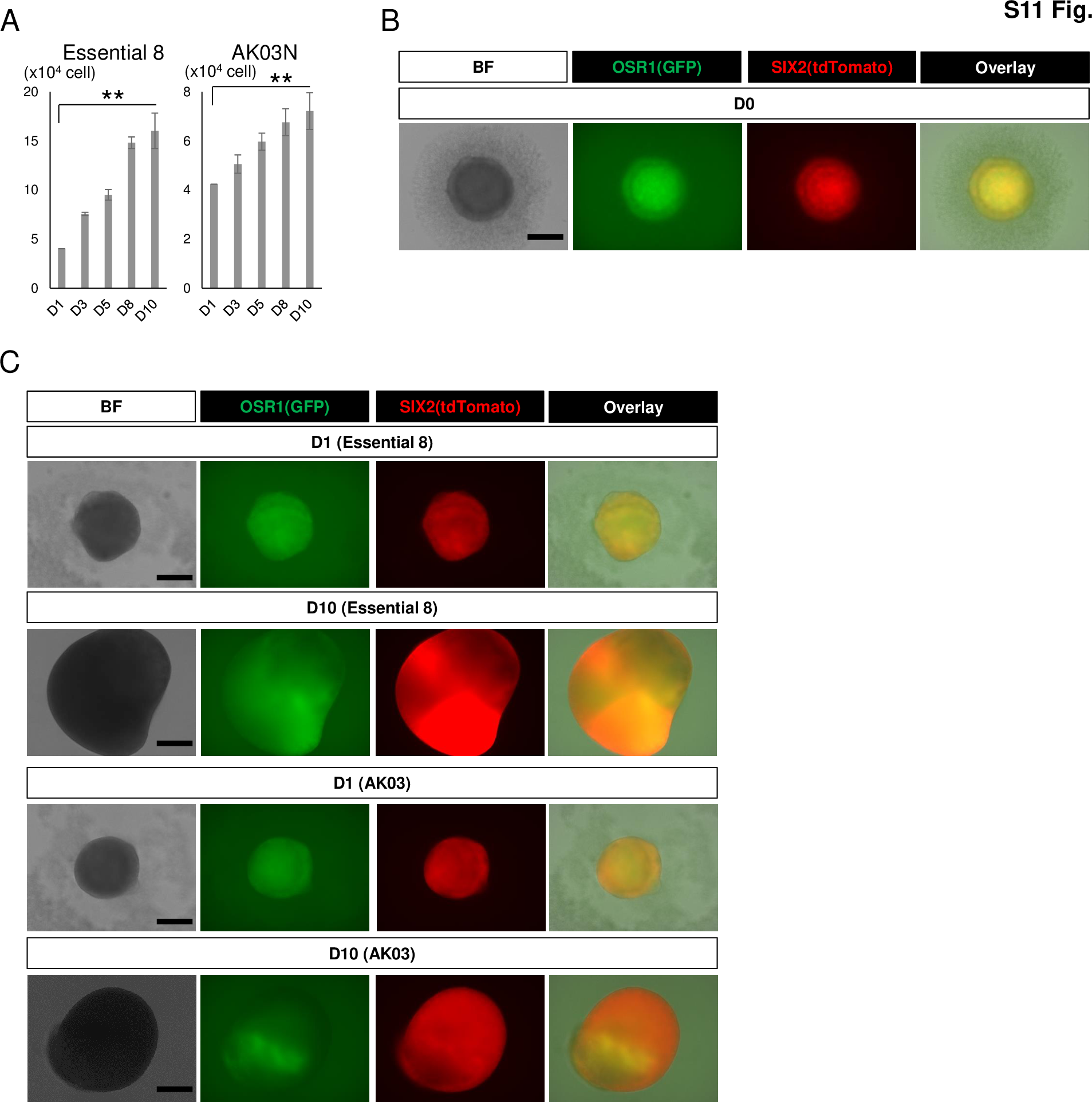

Supplement: S11 Fig — (A) The cell number of each cell aggregate treated with Essential 8 or AK03N media at 1, 3, 5, 8 and 10 days after seeding. (B, C) Representative brightfield or fluorescent images of OSR1 (GFP) SIX2 (tdTomato) from days 0 (B), 1 and 10 (C) cell aggregates treated with Essential 8 or AK03N media. **p <0.01 by paired Student’s t-tests comparing the cell number in day 1 (D1) and day 10 (D10) cell aggregates. Data are represented as the mean ± SEM (n = 12). Scale bars, 300 μm. (TIF) [file pone.0275600.s011.tif]

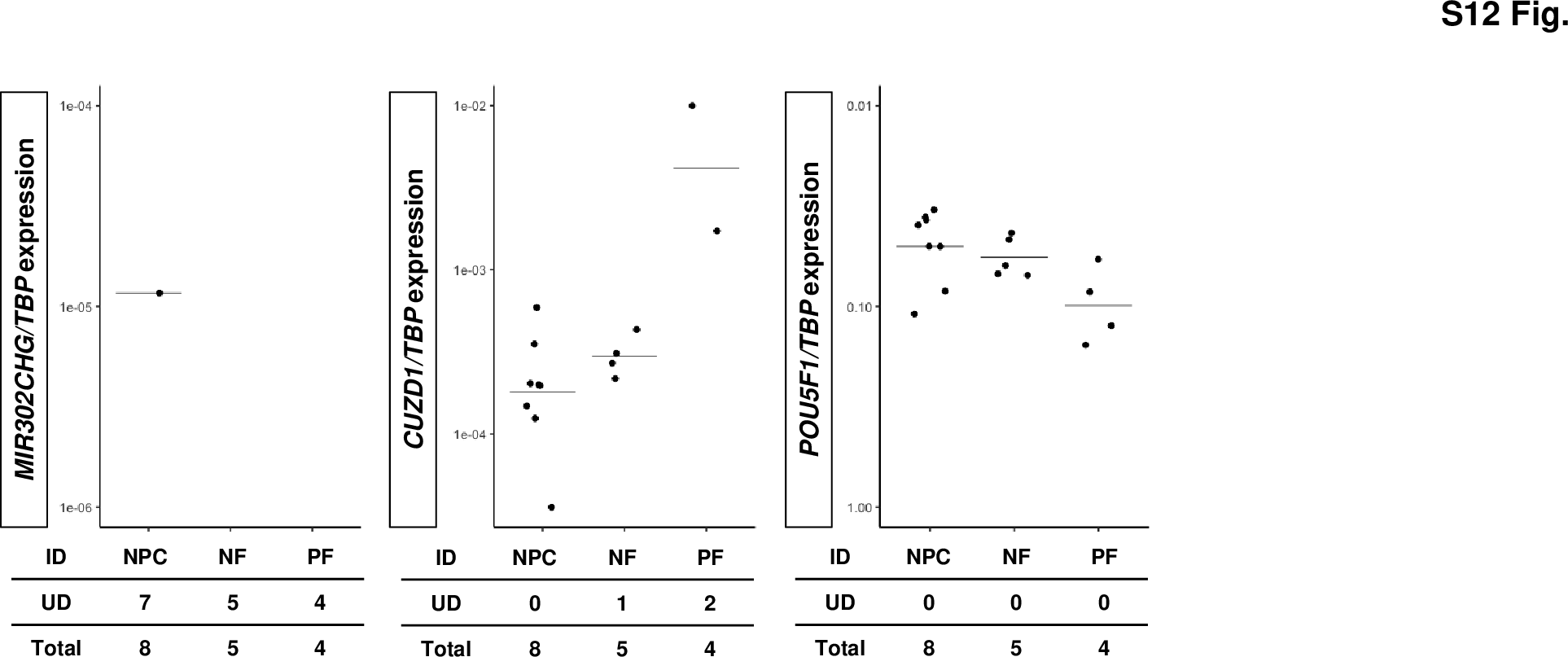

Supplement: S12 Fig — Scatter plots of the TBP-normalized gene expressions by qRT-PCR for MIR302CHG, CUZD1, and POU5F1 in NPCs, and MACS positive or negative fraction flowthroughs of the NPCs (PF or NF). The dots and lines in the center of the scatter plots indicate the experimental data and mean values of the data, respectively. UD: number of samples with undetermined CT values. (TIF) [file pone.0275600.s012.tif]
