# Supplementary material for: In vitro methods to ensure absence of residual undifferentiated human induced pluripotent stem cells intermingled in induced nephron progenitor cells
Source: PLoS One. 2022 Nov 15;17(11):e0275600. doi: 10.1371/journal.pone.0275600 (PMC9665373; doi:10.1371/journal.pone.0275600)
Supplement: S1 Table — (DOCX) [file pone.0275600.s013.docx]

**S1 Table. Highly expressed genetic markers (log2FC > 10 & FDR <10^-5^) in hiPSCs compared to day 4 cells from the NPC differentiation protocol.**

| Gene | log2FC | FDR | Classification |
| --- | --- | --- | --- |
| MIR302CHG | 15.919568 | 8.93E-30 | lncRNA |
| LINC00678 | 15.597548 | 1.36E-29 | lncRNA |
| CUZD1 | 14.526835 | 3.98E-12 | Other |
| FOXD3-AS1 | 14.045298 | 3.53E-24 | lncRNA |
| MARVELD3 | 12.878192 | 2.72E-20 | Other |
| IDO1 | 12.833227 | 1.63E-18 | Enzyme |
| CALB1 | 12.771838 | 8.29E-20 | Enzyme |
| TMEM30B | 12.651093 | 8.12E-19 | Transporter |
| TJP3 | 12.4513 | 3.93E-17 | Other |
| NPTX1 | 12.415639 | 1.29E-14 | Other |
| RP11-209K10.2 | 12.327168 | 2.20E-18 | lncRNA |
| LINC02700 | 12.319703 | 4.22E-17 | lncRNA |
| FOXD3 | 12.294333 | 6.64E-18 | Transcription regulator |
| LAD1 | 12.263516 | 2.16E-08 | Other |
| CR2 | 12.236595 | 6.45E-18 | Transmembrane receptor |
| BICDL2 | 12.191143 | 3.17E-17 | Other |
| SLC27A2 | 11.933075 | 4.02E-17 | Transporter |
| HLA-DPB2 | 11.866938 | 7.28E-17 | Other |
| RP11-69I8.2 | 11.555165 | 4.37E-05 | lncRNA |
| SYT4 | 11.52145 | 8.64E-16 | Transporter |
| TLE2 | 11.367715 | 1.14E-14 | Transcription regulator |
| TINAGL1 | 11.286966 | 2.07E-09 | Transporter |
| TRBC2 | 11.25961 | 4.00E-15 | Other |
| SIX6 | 11.245342 | 5.03E-08 | Transcription regulator |
| GRID2 | 11.140598 | 1.83E-19 | Ion channel |
| CCKBR | 11.103719 | 1.22E-07 | G-protein coupled receptor |
| JAKMIP2-AS1 | 10.917093 | 1.16E-05 | lncRNA |
| MYH2 | 10.8297 | 5.14E-12 | Enzyme |
| BSPRY | 10.764719 | 1.25E-13 | Other |
| NANOG | 10.48304 | 8.97E-12 | Transcription regulator |
| TDGF1P3 | 10.467367 | 2.89E-17 | Other |
| RAB17 | 10.405785 | 1.55E-31 | Enzyme |
| VWC2 | 10.332885 | 1.09E-11 | Other |
| MFSD6 | 10.332837 | 1.48E-12 | Transmembrane receptor |
| HTR2C | 10.329044 | 1.86E-11 | G-protein coupled receptor |
| ALDH1A1 | 10.152499 | 3.38E-12 | Enzyme |
| RIPOR2 | 10.149614 | 9.51E-15 | Other |
| RP11-1144P22.1 | 10.146431 | 3.73E-15 | lncRNA |
| SLC52A3 | 10.10358 | 1.60E-06 | Transporter |
| NMRK2 | 10.098372 | 4.00E-16 | Kinase |
| CHST4 | 10.084789 | 2.17E-16 | Enzyme |
| LNCPRESS2 | 10.080748 | 1.48E-11 | lncRNA |
| MBP | 10.065631 | 3.83E-16 | Other |
| ZNF322P1 | 10.048407 | 1.97E-11 | Pseudogene |
