# Supplementary material for: In vitro methods to ensure absence of residual undifferentiated human induced pluripotent stem cells intermingled in induced nephron progenitor cells
Source: PLoS One. 2022 Nov 15;17(11):e0275600. doi: 10.1371/journal.pone.0275600 (PMC9665373; doi:10.1371/journal.pone.0275600)
Supplement: S2 Table — (DOCX) [file pone.0275600.s014.docx]

| **S2 Table.** **Primer sequences for qRT-PCR used in this study.** | | |  |
| --- | --- | --- | --- |
| **Gene** | **Forward** | **Reverse** |  |
| MIR302CHG (ENST00000509938.1&ENST00000505215.1) | | |  |
| #1 | CCCTTCTGGAGGAGAACACG | GGAGATCCAAAGGGATTTGCG |  |
| #2 | CCTTCTGGAGGAGAACACGA | AGCAGCAAAAGCAATTGAGGT |  |
| #3 | ACCCTTCTGGAGGAGAACAC | AGGGATTTGCGTTTGTGGAA |  |
| #4 | CCTTCTGGAGGAGAACACGAA | AAGGGATTTGCGTTTGTGGA |  |
| #5 | CCCTTCTGGAGGAGAACACGA | AAGGGATTTGCGTTTGTGGAA |  |
| MIR302CHG (ENST00000509938.1) | | | |
| #6 | CCTTCTGGAGGAGAACACGAA | GAGGAAGAGAAGCAGCTCTGA |  |
| #7 | ACCCTTCTGGAGGAGAACACG | AGCAGCTCTGAAGATGCAGAAT |  |
| #8 | CCCTGTTCTGACTTCAGGATCT | GGGATTTGCGTTTGTGGAATTTG |  |
| #9 | AGGAGAACACGAATCTTTGGG | GAGGAAGAGAAGCAGCTCTGAA |  |
| #10 | TTCTGGAGGAGAACACGAATCT | AGGAAGAGAAGCAGCTCTGAA |  |
| #11 | CTTCTGGAGGAGAACACGAATCT | AGAGGAAGAGAAGCAGCTCTGA |  |
| #12 | GAGGAGAACACGAATCTTTGGGA | TGAAGTCAGAACAGGGAAGAGG |  |
| #13 | ACTAGTTCAGGAAGGGATTCTGC | GATCCTGAAGTCAGAACAGGGA |  |
| #14 | GGAGAACACGAATCTTTGGGAA | CCTGAAGTCAGAACAGGGAAGA |  |
| #15 | TCAGGAAGGGATTCTGCATCTT | GTCCAGATCCTGAAGTCAGAACA |  |
| Others | | |  |
| TDGF1 | CCCTCCTTCTACGGACGGAA | CAGGGAACACTTCTTGGGCAG |  |
| SPTSSB | GCTGTGCTGTTTTAGAGCCCT | CCAGGCGAATGTGGATTGG |  |
| VRTN | AGGGCATGATCGACTCCAAAG | ACAGAGGCGAGAGCATACAAG |  |
| POU5F1 | GAAACCCACACTGCAGCAGA | TCGCTTGCCCTTCTGGCG |  |
| PSMD5 | CTGTAGCAAAAGCGGCTATCA | CACCCTGTATCGAACAATGTCA |  |
| MT1E | TCAGGTTGGGAGGGAACTCAA | GAAAGCCTGGAGAGGGAATGA |  |
| PMDM14 | ACACGCCTTTCCCGTCCTA | GGGCAGATCGTAGAGAGGCT |  |
| NANOG | ACAACTGGCCGAAGAATAGCA | GGTTCCCAGTCGGGTTCAC |  |
| AFP | AGTGAGGACAAACTATTGGCCT | ACACCAGGGTTTACTGGAGTC |  |
| NODAL | CAGTACAACGCCTATCGCTGT | TGCATGGTTGGTCGGATGAAA |  |
| CUZD1 | ATGGAGCTTGTAAGAAGGCTCA | GAGTTGCAGGATCATGGCTTT |  |
| GDF3 | TCCTGGAGATACTGGTCAAAGAA | GAGCATCTTAGTCTGGCACAG |  |
| SOX3 | GACCTGTTCGAGAGAACTCATCA | CGGGAAGGGTAGGCTTATCAA |  |
| OSR1 | GCTGTCCACAAGACGCTACA | CCAGAGTCAGGCTTCTGGTC |  |
| POU5F1 | GAAACCCACACTGCAGCAGA | TCGCTTGCCCTTCTGGCG |  |
| SOX2 | TACAGCATGTCCTACTCGCAG | GAGGAAGAGGTAACCACAGGG |  |
| SIX2 | AGGAAAGGGAGAACAACGAGAA | GGGCTGGATGATGAGTGGT |  |
| TBP | GAGCCAAGAGTGAAGAACAGTC | GCTCCCCACCATATTCTGAATCT |  |
