# Supplementary material for: In vitro methods to ensure absence of residual undifferentiated human induced pluripotent stem cells intermingled in induced nephron progenitor cells
Source: PLoS One. 2022 Nov 15;17(11):e0275600. doi: 10.1371/journal.pone.0275600 (PMC9665373; doi:10.1371/journal.pone.0275600)
Supplement: S3 Table — (DOCX) [file pone.0275600.s015.docx]

| **S3 Table.** **Antibodies and lectin used in this study.** | | |  |
| --- | --- | --- | --- |
| Target for antibody or lectin | Source | Identifier |  |
| SIX2 | Proteintech | 11562-1-AP |  |
| PAX2 | R&D | ﻿AF3364 |  |
| PAX8 | Proteintech | 10336-1-AP |  |
| CDH1 | BD | BD610181 |  |
| ﻿PODX | R&D | ﻿AF1658 |  |
| LTL | ﻿Vector laboratories | ﻿B-1325 |  |
| ﻿Alexa Fluor 546 Donkey Anti-Rabbit IgG (H+L) | ThermoFisher | ﻿A10040 |  |
| ﻿Alexa Fluor 647 Donkey Anti-Goat IgG (H+L) | ThermoFisher | ﻿A21447 |  |
| ﻿Alexa Fluor 647 Donkey Anti-Rabbit IgG (H+L) | ThermoFisher | ﻿A31573 |  |
| ﻿Alexa Fluor 488 Donkey Anti-mouse IgG (H+L) | ThermoFisher | ﻿A21202 |  |
| ﻿Alexa Fluor 546 Donkey Anti-Goat IgG (H+L) | ThermoFisher | ﻿A11056 |  |
| ﻿Streptavidin, DyLight 405 conjugate | ThermoFisher | ﻿21831 |  |
