# Supplementary material for: In vitro methods to ensure absence of residual undifferentiated human induced pluripotent stem cells intermingled in induced nephron progenitor cells
Source: PLoS One. 2022 Nov 15;17(11):e0275600. doi: 10.1371/journal.pone.0275600 (PMC9665373; doi:10.1371/journal.pone.0275600)
Supplement: S4 Table — (DOCX) [file pone.0275600.s016.docx]

| **S4 Table. RNA sequencing data used in this study.** | | | |
| --- | --- | --- | --- |
| Material | Source | Identifier | Location used in this paper |
| ﻿A human fetal transcriptional atlas | Roost et al. 2015 | ﻿ ﻿GEO: GSE66302 | S1 Fig |
| Kidney organoids | Takasato et al. 2015 | ﻿ ﻿GEO: GSE70101 | S1 Fig |
| Hepatocyte-like cells | Kotaka et al. 2017 | GEO: GSE83480 | S1&S8 Fig |
| Pancreatic progenitors | Kimura et al. 2020 | ﻿GEO: GSE153806 | S1&S8 Fig |
| ﻿Liver organoids | Ouchi et al. 2019 | GEO: GSE130074 | S1&S8 Fig |
| Induced NPCs | Tsujimoto et al. 2020 | ﻿GEO: GSE146119 | Fig 1, S3 &S4 Fig |
| iPSCs | Matsuda et al. 2020 | GEO: ﻿GSE116935. | Fig 1&S3 Fig |
| hiPSCs and induced NPCs | This paper | CR3-002 (Nucleotide sequence data are available upon request) | Fig 2, S7 Fig &S1 Dataset (transcript count data) |
| Endoderm lineages | Loh et al. 2014 | GEO: GSE52657 | S8 Fig |
| hESCs and three germ layer lineage cells | Cliff et al. 2017 | GEO: GSE101655 | S9 Fig |
| hiPSCs and neuron lineages | Chen et al. 2013 | GEO: GSE43143 | S9 Fig |
| hiPSCs and cardiomyocytes | ﻿Banovich et al. 2018 | GEO: GSE107654 | S9 Fig |
| Lung lineages | ﻿Kerschner et al. 2020 | GEO: GSE136858 | S9 Fig |
| hESC and lung lineages | Jacob et al. 2017 | GEO: GSE96642 | S9 Fig |
| hiPSCs, hESCs, cardiomyocytes and endothelial cells | Zhao et al. 2017 | GEO: GSE94267 | S10 Fig |
