# Supplementary material for: In vitro methods to ensure absence of residual undifferentiated human induced pluripotent stem cells intermingled in induced nephron progenitor cells
Source: PLoS One. 2022 Nov 15;17(11):e0275600. doi: 10.1371/journal.pone.0275600 (PMC9665373; doi:10.1371/journal.pone.0275600)
Supplement: S5 Table — (DOCX) [file pone.0275600.s017.docx]

| **S5 Table.** **Primer and probe sequences for ddPCR used in this study.** | | | |
| --- | --- | --- | --- |
| **Gene** | **Forward** | **Reverse** | **Probe** |
| MIR302CHG (ENST00000509938.1) | CTGCTTCTCTTCCTCTTC | GCGTTTGTGGAATTTGAA | ACTTCAGGATCTGGACTTACCTCAATT |
| CUZD1 | Bio-Rad UniqueAssayID: dHsaCPE5037182 | | |
| TBP | Bio-Rad UniqueAssayID: dHsaCPE5058363 | | |
