# Supplementary material for: In vitro methods to ensure absence of residual undifferentiated human induced pluripotent stem cells intermingled in induced nephron progenitor cells
Source: PLoS One. 2022 Nov 15;17(11):e0275600. doi: 10.1371/journal.pone.0275600 (PMC9665373; doi:10.1371/journal.pone.0275600)
Supplement: S6 Table — (DOCX) [file pone.0275600.s018.docx]

| **S6 Table. ﻿Recombinant DNA used in this study.** | | | |
| --- | --- | --- | --- |
| **Material** | **Source** | **Reference** | **Location used in this paper** |
| ﻿ pPV-EF1a-EiP-A | A gift from Prof. Hotta | ﻿41, 42 | Figure S5 |
| pHL-EF1a-hcPBase-A | A gift from Prof. Hotta | 41 | Figure S5 |
| **Primers for EGFP-to-tdTomto replace** | | | |
| **Material** | **Sequences** | | |
| NcoI-tdTomato | 5’-GAGAATTAGCCATGGTGAGCAAGGGCGAGGAGGTCATCAA-3’ | | |
| tdTomato-EcoRI | 5’-AGAGGGGCGGAATTCTTACTTGTACAGCTCGTCCATGCCG-3' | | |
