# Supplementary material for: In vitro methods to ensure absence of residual undifferentiated human induced pluripotent stem cells intermingled in induced nephron progenitor cells
Source: PLoS One. 2022 Nov 15;17(11):e0275600. doi: 10.1371/journal.pone.0275600 (PMC9665373; doi:10.1371/journal.pone.0275600)
Supplement: S1 File — (ZIP) [file pone.0275600.s021.zip › S1_documents/585A1_ICF_ippan_r0091_20160407.pdf]

20151001（(Supplement 1)

Information for Patients or Healthy Family Members of Patients

Study title:

The Generation of Human Disease-Specific iPS Cells and the Use of Such iPS Cells for Disease Analysis

1. Your participation is voluntary and you are free to withdraw your consent at any time

You are free to choose to participate or not to participate in the study "The Generation of Human Disease-Specific iPS Cells and the Use of Such iPS Cells for Disease Analysis." If you change your mind later, you may withdraw your consent at any time. Your participation is voluntary. If you are not of legal age but are 16 years or older, both you and your legal representative are responsible for deciding whether or not to participate. If you are younger than 16 years, your legal representative is responsible for deciding whether or not you should participate. If you are an adult patient and it is difficult to confirm your understanding and intention to participate in this research, we may ask your legal representative to decide. You have no obligation to participate in the study. Your decision to participate or not to participate will have no influence on your current and future relationship with our hospital. We will always provide you with the treatment that is in your best interests.

If you agree to donate a sample of your cells after reading this information document and listening to your doctor, please sign or print your name with your seal on the informed consent form (Attachment).

If you consent to the study and change your mind later, you may withdraw your consent by just writing to us. You do not have to explain the reason. There is no penalty or loss of benefits if you decide to leave the study. If you withdraw your consent, the specimen you have donated, the iPS cells generated from your cells, and medical information associated with the donated specimen and iPS cells derived from the specimen will be destroyed and will not be used for research from that time on. Note that, however, recovery and disposal of your specimens may sometimes be difficult at the time when you withdraw your consent; for example, when the study using your specimens has made certain progress, a paper including data from the study has been published, or iPS cells generated from your specimen have been distributed to other institutions from a cell bank (this will be described later in this leaflet). In such cases, use of your specimen and iPS cells derived from the specimen and/or

the data obtained from these specimens may continue despite your withdrawal of consent.

The original of the signed informed consent form for the present study will be kept by the hospital. You will be given a duplicate of the original informed consent form.

2. The plan of the study has been approved by the Ethics Committee

The plan of this study has been reviewed by the Medical Ethics Committee at the Graduate School of Medicine and the University Hospital, and approved by the dean of the Graduate School of Medicine, Kyoto University and the director of Kyoto University Hospital.

3. Purpose of the study

Many types of treatments and various combinations of treatments, such as combination of drug with rehabilitation, have been used to improve your disease, or the patient's disease if you are a legal representative of the patient. However, the currently available treatments are not perfect. Researchers all around the world are working to develop better treatments. Development of new treatments requires many studies to be conducted in order to find out more about the disease, for example, what causes the disease, what type of drugs can cure the disease or improve disease symptoms and, if such drug is found, whether the drug is safe for use, etc. The ideal way of studying the disease is to use the diseased part of the patient's body (tissue). However, the use of diseased tissue involves many issues, for example, sampling of the diseased tissue may impose a severe burden on the patient or is sometimes technically impossible; in addition, because the amount of diseased tissue obtainable is limited, the diseased tissue cannot be used repeatedly for research.

Recently, an innovative technique, which can reduce the burden on patients, has been developed by Kyoto University. This innovative technique enables the generation of iPS cells from skin cells, as you may have seen in newspapers and on TV. Induced pluripotent stem cells, abbreviated as iPS cells, are generated by introducing 3 or 4 types of genes into the cells extracted from skin tissue. As the name “pluripotent stem cell” indicates, iPS cells can be differentiated into the various tissues that comprise our body. This feature can be used to develop, for example, blood cells if a researcher wants to study disease affecting blood cells, liver cells if a researcher wants to study liver disease, or nerve cells to study neurological disease. Because all these specific cells can be developed from iPS cells in test tubes, there is no need to ask a patient to donate his/her tissue over and over again.

On the other hand, researchers are also working to generate iPS cells from various organ cells (stomach, liver, cheek, blood, and bone marrow cells) as well as from skin tissue. In mice, it is now possible to generate iPS cells from stomach or liver cells. We now know that

the property of iPS cells is somewhat different depending on what type of body cell is used to generate the iPS cells. Researchers expect to use iPS cells of different origin depending on what type of treatment the researcher is trying to develop. Because of this, human iPS cells of different origin including human cells extracted from the cheek, blood, bone marrow, stomach, and liver need to be generated.

We are asking you to participate in this study because we want to generate iPS cells from your body cells in order to find out more about the cause of the disease for which you are currently being treated, or the patient's disease if you are a legal representative of the patient, and to develop new effective treatments.

Please note that it will take a number of years to develop a new treatment based on the data obtained in this study. We do not use the human iPS cells generated in this study for treatment, for example, the modified iPS cells will not be directly put back into the patient's body as treatment.

Also, we think it is especially important that the cells collected as well as information and data obtained in this study will be registered in public resource banks such as the those of RIKEN Bioresource Center and National Bioscience Database Center to make them readily available to research institutions (including laboratories inside pharmaceutical companies) in and outside Japan (this will be discussed in more detail later). This will help researchers working in a range of fields to bring together ideas and experiences in iPS cell research and facilitate elucidation of the mechanisms of currently incurable diseases and the development of new treatments.

4. Administrative organization of the study

This study will be conducted as collaborative research by Shinya Yamanaka (Professor at Center for iPS Cell Research and Application, Kyoto University) and the hospital departments at Kyoto University Hospital. The study will be supervised by Tatsutoshi Nakahata (Professor at the Center for iPS Cell Research and Application). The subinvestigators in charge of individual clinical departments are listed in Appendix 1. The intended study period is from the date of approval to March 31, 2018. However, depending on the progress of the study, the study period may be extended after approved by the Ethics Committee.

5. Study procedures

This study involves obtaining a sample of your body tissue (referred to as “Somatic cells” and described in the next section). The sample is used to extract cells. The extracted cells will be then sent to the Kyoto University Institute for Frontier Medical Sciences or

Kyoto University Center for iPS Cell Research and Application where iPS will be generated. If the Kyoto University personnel to generate iPS cells becomes insufficient or the working pipeline at Kyoto University cannot meet the needs for iPS cell generation to further the study, the generation of iPS cells may be conducted under contract by a for-profit entity that will be selected fairly and properly by Kyoto University Center for iPS Cell Research and Application. In this case, the Somatic cells will be transported from Kyoto University Center for iPS Cell Research and Application to the contractor by appropriate means. The Somatic cells will be coded (to be explained herein below) prior to the transportation, therefore carrying no personal information (such as name) that can identify you. Such outsourcing of iPS cell generation will be subject to the review and approval by the contractors internal ethical committee in advance.

iPS cells are currently generated by introducing genes using viral components. In the future, however, more effective and safer techniques may become available. We will use the most suited method available at the time. The iPS cells generated in this study will be used for research to find out the cause of the disease and to develop new treatments.

Sometimes healthy and diseased cells need to be compared in order to assure the reliability of the disease study. We may ask healthy family members of the patient or people requiring treatment or undergoing surgery (as part of regular treatment not related to this study) in plastic and reconstructive surgery or orthopedic surgery to donate body tissue to generate iPS cells. In such cases, the iPS cells will be generated and used in accordance with the same rules applied to the generation and use of iPS generated from patients. If you (healthy family members of the patient or surgery patient) agree to this study, healthy family members of the patient will be asked to sign the same form as the consent form for patients, and those who receive treatment or undergoing surgery in plastic and reconstructive surgery or orthopedic surgery will be asked to sign another consent form for healthy volunteers.

6. Sampling of body tissue

Before the sampling procedure, you (study participants) will be asked to take blood tests to see if you are infected with certain viruses. We can make the test results available to you at your request. We will decide whether or not to take a tissue sample from you based on the results of the screening tests for virus infection.

A sample of one of the following tissue types will be taken from you. The sample will be used to extract cells.

1) Skin: A piece of skin will be taken from an area where the scar will be minimally visible (e.g., inner thigh or inner upper arm). The area will first be disinfected, and then made numb by local anesthesia (injection). Then, a piece of skin will be taken

from the area using a 3-5 mm metal punch (trepan). After a piece of skin is taken from the area, the wound is usually sutured with a single stitch, and a sterile dressing will be applied to the area. The suture can be removed after 1 week or so. The skin sample will be cultured in a laboratory to increase the number of skin cells by several hundred-fold, and then used to generate iPS cells. Except for the discomfort related to the sampling procedure, there will no serious risk associated with sampling of skin tissue. However, infants need to be held tight during the procedure, which may impose a psychological burden on the infants. The most practical risk is that if you scratch the wound later, some complications may occur; for example, bacteria may get into the wound and develop an infection (pus) or the wound may reopen. However, since we keep the area of biopsy clean, the development of such a complication is extremely rare in our experience.

2) Cheek cells (buccal mucosa cells): A sample of cells will be collected by gently scraping the inside of the cheek with a cotton swab.
3) Blood: A sample of blood will be collected using the same procedure as for standard blood tests.
4) Bone marrow: A sample of bone marrow will be collected by either of the following 2 methods. One is to obtain a sample using the standard bone marrow biopsy procedure. After administering local anesthesia to reduce the pain, a sample will be taken from the sternum or ilium. A bone marrow aspiration needle will be inserted through the skin to the bone surface and then into the bone marrow, and a sample of bone marrow fluid will be aspirated using a syringe. The other method is to obtain a sample when a bone graft is taken from the ilium during orthopedic surgery. A sample of bone marrow cells will be taken from the site of the bone graft on the ilium using an aspiration needle. There will be no pain because these procedures are performed under systemic or lumbar anesthesia.
5) Stomach tissue (gastric mucosa): A sample of stomach tissue can be obtained from a portion of the stomach removed by surgery, or a sample can be obtained during endoscopy. Patients will be given information about the donation of a tissue sample when the patient undergoes stomach surgery or endoscopy.
6) Liver tissue: A sample of liver tissue will be obtained from a portion of the liver removed by surgery. Patients will be given information about the donation of a tissue sample when the patient undergoes surgery.
7) Lung tissue: A sample of lung tissue will be obtained from a portion of the lung removed by surgery as treatment or by biopsy. Patients will be given information about the donation of a tissue sample when the patient undergoes surgery.

8) Oral mucosa: A specimen will be obtained during oral surgery. A piece of oral mucosa will be collected from the tissue excised from a surgical incision. No additional incision or invasive procedure is required for donation of your sample.
9) Wisdom tooth germs, extracted tooth, and deciduous tooth pulp: Dental pulp will be collected from a tooth that needs to be extracted for medical reasons or a deciduous tooth that has fallen out. No additional incision or invasive procedure is required for donation of your sample.
10) Urogenital tissue: A sample of urogenital tissue will be collected from the kidney removed from patients undergoing a kidney transplant. Tumor and healthy tissues excised during surgery for a urogenital tumor, etc. will also be used. No additional incision or invasive procedure is required for donation of your sample.
11) Heart tissue: A sample of heart tissue will be collected, for instance from a surgical incision in the heart or from partial heart resection. Patients will be given information about the donation of a tissue sample when the patient undergoes surgery.

7. Use of iPS cells generated in this study

The iPS cells generated in this study will be used to help find the cause of your disease or to develop new treatments. However, the iPS cells will not be used as an actual treatment. There are detailed regulatory rules regarding the use of embryonic stem cells (ES cells), which, like iPS cells, can be developed into various types of cells. On the other hand, there are no detailed rules regarding the use of iPS cells. Currently, the following uses of iPS cells are prohibited.

1) Generation of whole bodies from human iPS cells by either transplantation of the embryo, which develops into a fetus, that has been developed from human iPS cells into a human or animal uterus, or any other means
2) Introduction of human iPS cells into human embryos
3) Introduction of human iPS cells into human fetuses
4) If germ cells, sperms and eggs, are developed from human iPS cells, the use of such germ cells to develop human embryos.

These 4 rules will be strictly applied to the use of iPS cells generated in this study. In the future, the laws and guidelines may be revised or deregulated. If the laws and guidelines are revised in the future, we will use iPS cells in compliance with the revised laws and guidelines, following necessary steps accordingly which might include revisiting your consent decision, your reaffirmation or re-consent.

8. Gene analysis

In order to make progress in research that utilizes the iPS cells generated in this study, we may need to analyze the genes of the cells. We have to make a separate plan for gene analysis. The plan will be submitted to the committee in charge of reviewing human gene analysis studies at Kyoto University for review. The plan must be approved by the committee before starting the gene analysis. Thus, we would like to give you an explanation about the gene analysis using a separately prepared information consent document. If you agree to the gene analysis, your sample will be subjected to gene analysis.

9. Your personal information

Your Somatic cells and the iPS cells generated from the Somatic cells in this study will be given a code name after removing the personal information (name, address, etc.) that can identify you. This procedure is called anonymization. The link table used to link the code with patient's personal information will be managed by a personal information custodian who is not involved in this study. This prevents any person other than the custodian from knowing whose cells are used to generate the iPS cells. On the other hand, if information on new study findings is requested by the donor, the researcher will be able to obtain the patient's personal information from the custodian (this is called linkable anonymization), and give the donor the requested information with the proviso that providing the information does not diminish the originality of the study. Such personal information is obtainable only by the researchers involved in studies conducted at the Center for iPS Cell Research and Application, Kyoto University, the Institute for Frontier Medical Sciences, Kyoto University, and Graduate School of Medicine, Kyoto University.

10. Provision of specimens to third-party research institutions

Third-party researchers (researchers other than those who are participating in this study) may request your Somatic cells, ~~and~~ the iPS cells generated from the cells and the iPS cell-derived differentiated cells. If we receive such a request, we would like to supply the iPS cells to third-party researchers provided they meet the following criteria.

1) The plan of the study in which the cells will be used has been reviewed and approved by the Ethics Committee or equivalent of the third-party institution (unless the Ethics Committee or equivalent decides such review or approval is not required according to the applicable rules or guidelines) ; and
2) The study plan, including the purpose of the research, the methods, and how the study was reviewed by the third-party institution or how the institution determined it may proceed with the study etc., are considered to be appropriate by the researcher(s) who have been involved in the generation of the iPS cells.

* In order to effectively utilize the iPS cells to develop new treatments, it is important to encourage medical/pharmaceutical research conducted by commercial companies, including pharmaceutical companies. Thus, we would like to supply Somatic cells and iPS cells to companies if their research plan is appropriate and approved by an Ethics Committee or equivalent. Please note that the provision of the cells derived from you may be done through a for-profit entity that will be selected by Kyoto University Center for iPS Cell Research and Application. This may lead to the development and eventual marketing of new effective drugs that may be beneficial to you by pharmaceutical companies.

If you agree to the transfer of the iPS cells generated from your sample to third-party institutions, please choose “I agree” under the section “Provision of iPS cells to third-party institutions” in the consent form. If you do not want to let us provide iPS cells generated from your sample under any circumstances, please choose “I do not agree.” Your decision to agree or not to agree to the provision of the iPS cells generated from your sample will not affect your regular medical care. The iPS cells will be supplied to third-parties after they are anonymized in a linkable manner. Thus, if you later withdraw your consent, we will contact the third-party institution and tell them to stop using the iPS cells generated from your sample.

11. iPS cell banking and database registration

RIKEN Bioresource Center (hereinafter abbreviated as RIKEN BRC) organizes an iPS cell bank in order to help researchers conduct research using various iPS cells. RIKEN BRC has received the support from the national government (from the Ministry of Education, Culture, Sports, Science and Technology [MEXT]) and has gathered and distributed a large number of iPS cells from and to researchers in and outside Japan. RIKEN BRC has ample resources for preservation of iPS cells and gives lectures on the techniques for handling iPS cells. We would like to consider deposition of your Somatic cells and the iPS cells generated from your Somatic cells with RIKEN BRC so that many researchers can utilize them. If you agree, the iPS cells will be anonymized in a such manner that your personal information cannot be linked to the iPS cells at RIKEN BRC, and the cells will be then sent to RIKEN BRC. This will protect your privacy. RIKEN BRC will distribute your Somatic cells as well as iPS cells generated from your Somatic cells to researchers and institutions (including pharmaceutical companies) in and outside Japan together with data such as your medical records in accordance with proper procedures and the rules established by the Japanese government. The cells will then be used in research to elucidate the mechanisms of illnesses

and assist in the development of new treatments. Neither RIKEN BRC or us will contact you upon the distribution of your Somatic cells or iPS cells, but RIKEN BRC will release the cells only to research that has been judged to be appropriate by the specialist committee (Ethics Committee) of the institution to which belong the individual researchers requesting the cells.

Please make sure you understand and consider the meaning of the banking. If you agree to deposition of iPS cells to RIKEN BRC, please choose “I agree” under the section “Deposition of iPS cells to Cell Banks” in the consent form. If you do not agree, please choose “I do not agree.” Your decision to agree or not to the deposition of the iPS cells generated from your sample will not affect your regular medical care.

Data generated in this study including genetic information will also be useful for other medical research. Data obtained from you will be, after anonymization (removal of the information including your name and address that can be used to identify you), registered in publicly funded academic databases so that researchers can access the data. We plan to register data from this study in the database of the National Bioscience Database Center (NBDC) of the Japan Science and Technology Agency (JST). JST is an agency under MEXT and promotes and funds scientific research projects in Japan. NBDC was founded in 2011. Data registered in the NBDC will be made accessible to researchers from various fields and will help in the development of new technologies, elucidation of the mechanisms of currently incurable diseases, and discovery of new treatments and prophylactic therapies. If you agree to have your data registered in NBDC, please choose “I agree” under the section “Database registration” in the consent form. If you do not agree, please choose “I do not agree.” Your decision about data registration will not affect your regular medical care.

12. If you want to learn more about the study plan

If you want to learn more about the study plan, we can show you the study protocol excluding the portions of the protocol where information is confidential due to intellectual property rights, etc.

13. Publication of study data

Data obtained from this study may be presented at academic society meetings or published in academic journals or databases. However, we will take appropriate measures to ensure a donor's personal information is protected. Personal information of donors (e.g., name) will not be released to any third party, and will not appear in any presentations or publications. If you withdraw your consent during the study, the iPS cells generated from your sample will not be used for research from that time on, and thus, no new data will be

published. However, the data published (in reports, journals, etc.) prior to withdrawal of your consent will not be retractable.

14. Expected benefits and risks of participating in the study

Note that you will not receive immediate therapeutic benefits as a result of your participation in this study. Because research on iPS cells began very recently, it is uncertain if we can obtain useful research findings that can contribute positively to your actual treatment. Nevertheless, if the cause of your illness is discovered or a new drug or therapy is developed through participation in the study, you and others who have the same illness as you could potentially receive benefits in terms of disease diagnosis and treatment in the future. The expected risks are 1) the risks related to sampling of your tissue and 2) invasion of privacy due to leak of personal information. With regard to risk 1), we can reduce the risk by choosing the least invasive sampling method, and perform the sampling procedure for each tissue sample with care. With regard to risk 2), we will do everything we can do to protect the confidentiality of your personal information, this includes anonymization. Your personal information will be kept under strict security.

This clinical study is not covered by the clinical research liability insurance policy. Thus, if study-related injury occurs, you will be promptly provided with appropriate diagnostic and medical care using health insurance. There will be no cost to you regarding your treatment for study-related injury.

15. Preservation of samples and information after completion of the study

As already explained, your Somatic cells, the iPS cells generated from the cells and the iPS cell-derived differentiated cells are very valuable. Therefore, these cells along with the information obtained in the course of the study such as your genetic information, DNA or RNA will be preserved at Kyoto University as well as at the repositories if the cells are deposited and/or the information is registered for research use. The preservation period might be a long time even after the completion of this study because such cells and information could lead to new research findings in the future.

16. Intellectual property generated from this study

Intellectual property (e.g., patents) and intellectual property rights may be generated from the outcomes of the studies conducted using iPS cells generated from your tissue. Such intellectual property rights are not given to the donated sample itself but to the value generated by the work of researchers (research, the use of research outcomes, etc.). Thus, the donor or affiliates of the donor cannot claim the rights by saying, “Because the donor is the

one who donated the sample, the intellectual property rights related to the sample should be given to the donor.” For the same reason, if monetary profit is obtained from the intellectual property, the donor cannot claim the right to receive the profit. As a rule, all of the intellectual property is managed by Kyoto University.

17. Costs

All necessary research-related expenses will be paid through our research funds (government grants from MHLW and MEXT or research funds provided through industry-university collaboration). There will no cost to you (Supplements 5 and 6).

18. Contact information

If you have questions or concerns about your participation in this study, please feel free to call your study doctor.

Your study doctor: ______________________
TEL: ______________________

19. Please note that such iPS cells generated from a healthy family member of the patient will be used in comparisons with any type of disease without limitation.

Please take your time to read the document carefully until you fully understand the information given in this informed consent document. After carefully reading this document, if you choose to participate in this study, please sign and date the consent form (Attachment) and give the signed consent form to your study doctor.

Date:

Doctor who conducted the informed consent discussion: (signature)

20151001（(Supplement 2)

Information for Healthy Volunteers Participating in This Study

(Please take your time to read the document carefully before you decide if you are willing to participate)

Study title:
The Generation of Human Disease-Specific iPS Cells and the Use of Such iPS Cells for Disease Analysis

- ◆ The purpose of this consent document is to give you information to help you decide if you want to participate in the study.
- ◆ Before you make your decision, please read this document carefully until you fully understand the information contained in the document. Please decide whether you agree or not agree to participate in the study on the basis of your own free will.
- ◆ If you have any questions or concerns, please feel free to ask your study doctor.
- ◆ Your participation in this study is voluntary. If you decide not to participate, there will be no penalty or loss of benefits to which you are otherwise entitled.
- ◆ If you consent to this study, you may withdraw your consent at any time. It is OK to change your mind after you have given your consent. If you withdraw your consent, there will be no penalty or loss of benefits to which you are otherwise entitled.

1.Introduction

In our hospital, we are conducting research to develop more efficient treatments for patients suffering from intractable disease. The development of new treatments requires many studies to be conducted in order to find out more about the disease, for example, what causes the disease, what type of drugs can cure the disease or improve disease symptoms and, if such a drug is found, whether the drug is safe for use, etc. For this research, we need help from both healthy volunteers and patients to conduct the medical studies. Clinical studies are a type of medical study aimed at finding the cause of a disease; to find ways to prevent, diagnose, and treat the disease more efficiently; and to improve the quality of life of patients. Please consider carefully the nature of clinical studies, which are a different aspect of medicine and distinct from regular medical care.

２. **The plan of this study has been approved by the Medical Ethics Committee:** In order to protect human rights and safety of participants, the plan of this study has been reviewed by the Medical Ethics Committee at Graduate School of Medicine, Kyoto University and the Kyoto University Hospital. The ethical and scientific adequacy of the study has been confirmed by the dean of the Graduate School of Medicine, Kyoto University and the director of Kyoto University Hospital.

３. **Purpose of the study:** As described in section 1 ‘Introduction,’ we need to find the cause of a disease in order to develop new treatments. The ideal way of studying the disease is to use the diseased part of a patient's body (tissue). However, the use of diseased tissue involves many issues, for example, sampling of the diseased tissue may impose a severe burden on the patient or is sometimes technically impossible; in addition, because the amount of diseased tissue obtainable is limited, the diseased tissue cannot be repeatedly used for research.

Recently, an innovative technique, which can reduce the burden on patients, has been developed by Kyoto University. This innovative technique enables the generation of iPS cells from the skin cells, as you may have seen in newspapers and on TV. Induced pluripotent stem cells, abbreviated as iPS cells, are generated by introducing 3 or 4 types of genes into the cells extracted from skin tissue.

As the name “pluripotent stem cell” indicates, iPS cells can be differentiated into the various tissues that comprise our body. This feature can be used to develop, for example, blood cells if a researcher wants to study disease affecting blood cells, or liver cells if a researcher wants to study liver disease, or nerve cells to study neurological disease. Because all these specific cells can be developed from iPS cells in Petri dishes, there is no need to ask

the patient to donate his/her tissue over and over again.

In order to find the cause of diseases, understand the properties of disease-affected cells of patients, and conduct various studies, it is extremely important to compare diseased cells with healthy cells (comparator cells). For this reason, we are asking you to participate in this study because we want to generate iPS cells from your body cells in order to help find out the cause of various diseases and to develop new effective treatments.

Also, we think it is especially important that the cells collected as well as information and data obtained in this study will be registered in public resource banks such as RIKEN Bioresource Center and National Bioscience Database Center to make them readily available to research institutions (including laboratories inside pharmaceutical companies) in and outside Japan (this will be discussed in more detail later). This will help researchers working in a range of fields to bring together ideas and experiences in iPS cell research and facilitate elucidation of the mechanisms of currently incurable diseases and the development of new treatments.

４. **Study procedures:** This study involves obtaining a sample of your body tissue. The sample is used to extract cells (these cells are called Somatic cells). The extracted cells will be then sent to Institute for Frontier Medical Sciences, Kyoto University or Center for iPS Cell Research and Application, Kyoto University where iPS will be generated using the most suitable method available at the time. The iPS cells generated in this study will be used in research to find out the causes of diseases and to develop new treatments.

If the Kyoto University personnel to generate iPS cells becomes insufficient or the working pipeline at Kyoto University cannot meet the needs for iPS cell generation to further the study, the generation of iPS cells may be conducted under contract by a for-profit entity that will be selected fairly and properly by Kyoto University Center for iPS Cell Research and Application. In this case, the Somatic cells will be transported from Kyoto University Center for iPS Cell Research and Application to the contractor by appropriate means. The Somatic cells will be coded (to be explained herein below) prior to the transportation, therefore carrying no personal information (such as name) that can identify you. Such outsourcing of iPS cell generation will be subject to the review and approval by the contractor’s internal ethical committee in advance.

Please note that it will take many years to develop a new treatment based on the data obtained in this study. We do not use the human iPS cells generated in this study as a treatment, for example, the modified iPS cells will not be used directly to put back into a patient's body as a treatment.

5．**Sampling of body tissue:** Before the sampling procedure, you will be asked to take blood tests to see if you are infected with certain viruses. We will decide whether or not to take a tissue sample from you, based on the results of screening tests for virus infection.

A sample of one of the following 2 types of tissue will be taken from you. The sample will be used to extract cells. The extracted cells will be then used to generate iPS cells.

1) Skin: In surgery, when the incision on the skin is sutured, sometimes a piece of skin becomes available as surplus. Using the surplus, we would like to obtain a square piece of skin measuring from 2–3 mm to 1 cm on a side. The collected skin sample will be cultured in a laboratory to increase the number of skin cells by several hundred-fold, and then used to generate iPS cells. Otherwise we will collect a small tissue sample by excision from an inconspicuous part of your body (e.g., inside of the arm) in a manner as painless as possible (called punch biopsy). The skin biopsy takes about one hour. Punch biopsy involves the following steps:

① Select an inconspicuous part of the body.
② Disinfect the skin and cover with a sterilized cloth.
③ The skin area is anesthetized to prevent you from feeling pain. (The anesthetic agent is administered by injection and does cause minor pain.)
④ A circular blade (punch) is inserted into the skin and rotated to collect a skin tissue sample about 5 mm in diameter and 5 mm in length.
⑤ Pressure is applied to the area to stop bleeding. One stitch may be used if necessary to close the wound. The suture will be removed after about a week.

2) Blood: A sample of blood will be collected using the same procedure as is standard in blood tests. The amount of blood collected is approximately 20 mL.

6．**Administrative organization of the study:** This study will be conducted as collaborative research by Shinya Yamanaka (Professor at the Center for iPS Cell Research and Application, Kyoto University) and the hospital departments at Kyoto University Hospital.

1) Person in charge of supervising the study as a whole
Tatsutoshi Nakahata (Deputy Director, Center for iPS Cell Research and Application)

2) The following researchers are in charge of conducting studies using skin samples obtained from surgery patients.

- Studies in plastic and reconstructive surgery: Shigehiko Suzuki (Professor, Graduate School of Medicine, Kyoto University), Motoko Naitoh (Lecturer, Graduate School of Medicine, Kyoto University)
- Studies in orthopedic surgery: Takeshi Okamoto (Lecturer, Kyoto University Hospital)

3) Researchers in charge of generating and preserving iPS cells

Shinya Yamanaka (Director, Center for iPS Cell Research and Application)
Takafumi Kimura (Professor, Center for iPS Cell Research and Application)
Isao Asaka (Professor, Center for iPS Cell Research and Application)
Megumu Saito (Associate Professor, Center for iPS Cell Research and Application)
Haruhisa Inoue (Professor, Center for iPS Cell Research and Application)
Kenji Osafune (Professor, Center for iPS Cell Research and Application)
Masato Nakagawa (Lecturer, Center for iPS Cell Research and Application)
Keisuke Okita (Lecturer, Center for iPS Cell Research and Application)

4) Study period: From the date of approval to March 31, 2018.

* Depending on the progress of the study, the study period may be extended after approved by the Ethics Committee.

7. Interview about your medical history

Because comparator cells are used to compare with iPS cells generated from patients with various diseases, we need information on your medical history to determine whether your cells are suitable as comparator cells. Your information registered in a separate interview form will be kept with the sample of your cells. The information registered in the interview form will be kept as your personal information under strict security, and will be used only for this study. When we supply or deposit your cells to the research institutions collaborating with us or to RIKEN Bioresource Center (RIKEN BRC) cell banks, they receive only the minimum of anonymized information that cannot identify you personally.

We may ask you for another interview about your health in several years. If you agree to this, we would like to contact you when we need further interview by using your personal information obtained from the personal information custodian mentioned below.

８. Expected benefits and risks of participating in the study

In terms of a contribution to your medical treatment, there will be no benefits to you from taking part in this study. Nevertheless, if the cause of some illness is discovered or a new drug or therapy is developed through participation in the study, a large number of people could potentially receive benefits in terms of disease diagnosis and treatment in the future. The expected risks are 1) the risks related to sampling of your tissue and 2) invasion of privacy due to leak of personal information. With regard to risk 1), we can reduce the risk by choosing the least invasive sampling method, and perform the sampling procedure for each tissue sample with care. If surplus skin from surgery is used, there will no additional burden on your body. If a blood or skin sample is collected from you, the sampling procedure may cause discomfort or bleeding.With regard to risk 2), we will do everything we can do to protect the confidentiality of your personal information, this includes anonymization. Your personal information will be kept under strict security.

This clinical study is not covered by the clinical research liability insurance policy. Thus, if study-related injury occurs, you will be promptly provided with appropriate diagnostic and medical care using health insurance. There will be no cost to you regarding your treatment for study-related injury.

９. **Costs to you:** All necessary research-related expenses (including the fees for tests) will be paid through our research funds (government grants from MHLW and MEXT or research funds provided through industry-university collaboration). There will be no cost to you.

１０. Your participation is voluntary and you are free to withdraw your consent at any time

Please take your time to consider the information in this document carefully before you decide if you are willing to participate. If you choose not to participate, there will be no penalty or loss of benefits to which you are otherwise entitled. We will always provide you with the treatment that is in your best interests.

If you consent to this study, you may withdraw your consent at any time you want to stop participating. If you want to stop participating, please contact your study doctor. There will be no penalty or loss of benefits if you decide to leave the study. If you stop participating in the study, the specimen you have donated, the iPS cells generated from your cells, and medical information associated with the donated specimen and the iPS cells will be destroyed and will not be used for research from that time on. Note that, however, recovery and disposal of your specimens may sometimes be difficult at the time when you withdraw your consent; for example, when the study using your specimens has made certain progress,

a paper including data from the study has been published, or iPS cells generated from your specimen have been distributed to other institutions from a cell bank (this will be described later in this leaflet). In such cases, use of these specimens and/or the data obtained from your specimens may continue despite your withdrawal of consent.

If you are not of legal age but 16 years or older, you and your legal representative is responsible for deciding whether or not to participate. If you are younger than 16 years, your legal representative is responsible for deciding whether or not you can participate. Please take your time to make your decision. You are under no obligation to participate in the study.

１１． **The iPS cells generated in this study will be used appropriately:** The iPS cells generated from your tissue will be used for research aimed at finding the cause of diseases and at developing new treatments. Such iPS cells will not be used as an actual treatment. Research on iPS cells has to be conducted in compliance with relevant laws and guidelines stipulated by the government. Specifically, as of April 2010, the following uses of iPS cells are prohibited.

1) Generation of whole bodies from human iPS cells by either transplantation of the embryo, which develops into a fetus, that has been developed from human iPS cells into a human or animal uterus, or any other means
2) Introduction of human iPS cells into human embryos
3) Introduction of human iPS cells into human fetuses
4) If germ cells, sperms and eggs, are developed from human iPS cells, the use of such germ cells to develop human embryos.

In the future, the laws and guidelines may be revised or deregulated. If the laws and guidelines are revised in the future, we will use iPS cells in compliance with the revised laws and guidelines, following necessary steps accordingly which might include revisiting your consent decision, your reaffirmation or re-consent.

１２． **Your personal information will be strictly protected:** Your privacy will always be protected. The Somatic cells and iPS cells generated from the Somatic cells in this study will be given a code name after removing the personal information (name, address, etc.) that can identify you personally. This procedure is called anonymization. Because your personal information will be handled and managed by a personal information custodian who is not involved in this study, any information that relates to your privacy will not be disclosed to the public.

In case you need to contact our researchers or in case our researchers need to contact you, the personal information will be anonymized in such a manner that we will be able to

contact you by obtaining your personal information from the personal information custodian (this is called linkable anonymization). Your personal information will be handled only in the Center for iPS Cell Research and Application, Kyoto University; the Institute for Frontier Medical Sciences, Kyoto University; and Graduate School of Medicine, Kyoto University. Your personal information will not be disclosed to the research institutions collaborating with us or RIKEN BRC described in section 15 ‘Provision of iPS cells to third-party research institutions’ and section 16 ‘iPS cell banking and database registration.’

１３. **Study data will be published after removing your personal information:** Data obtained from this study may be presented at academic society meetings or published in academic journals or databases. However, we will take appropriate measures to ensure that your personal information is protected. Your personal information (e.g., name) will not be disclosed to any third party, and will not appear in any presentations or publications. Thus, your privacy will be protected.

If you withdraw your consent during the study, the iPS cells generated from your sample will not be used for research from that time on, and thus, no new data will be published. However, the data published (in reports, journals, etc.) prior to withdrawal of your consent will not be retractable.

１４. **Gene analysis:** In order to make progress in research that utilizes the iPS cells generated in this study, we may need to analyze the genes of your cells (this is called gene analysis). In addition to the study plan described in the previous sections, we have to make a separate plan for gene analysis. The plan will be submitted to the committee in charge of reviewing human gene analysis studies at Kyoto University for review. The plan must be approved by the committee before starting the gene analysis. Thus, we would like to give you an explanation about the gene analysis using a separately prepared information consent document. If you or your legal representative agrees to the gene analysis, your sample will be subjected to gene analysis.

１５. **Provision of iPS cells to third-party research institutions:** In order to speed up the development of more effective treatments, third-party researchers or institutions other than Kyoto University may request your Somatic cells, and the iPS cells generated from the cells and the iPS cell-derived differentiated cells. If we receive such a request, we would like to supply the iPS cells to third-party researchers or institutions provided they meet the following criteria.

1) The plan of the study in which your cells will be used has been reviewed and approved

by the relevant Ethics Committee or equivalent of the third party (unless the Ethics Committee or equivalent decides such review or approval is not required according to the applicable rules or guidelines); and

2) The study plan, including the purpose of the research, the methods, and how the study was reviewed by the third-party institution or how the institution determined it may proceed with the study etc., are considered to be appropriate by the researcher(s) who have been involved in the generation of the iPS cells.

* In order to effectively utilize the Somatic cells and iPS cells to develop new treatments, it is important to encourage medical/pharmaceutical research conducted by commercial companies, including pharmaceutical companies. Thus, we would like to supply iPS cells to companies. Please note that the provision of the cells derived from you may be done through a for-profit entity that will be selected by Kyoto University Center for iPS Cell Research and Application. This may lead to the development and eventual marketing of new effective drugs that may be beneficial to patients by pharmaceutical companies.

If you agree to the donation of the cells generated from your sample to third-party institutions provided they meet the criteria above, please choose “I agree” under the section “Donation of iPS cells to third-party institutions” in the consent form. If you do not want to let us provide iPS cells, please choose “I do not agree.” Your decision to donate or not to donate the iPS cells will not affect your regular medical care. If you consent to the donation, you may withdraw your consent later at any time. We will contact the third-party institution and tell them to stop using the iPS cells generated from your sample.

１６．**iPS cell banking and database registration** As iPS research progresses, RIKEN Bioresource Center (hereinafter abbreviated as RIKEN BRC) organizes an iPS cell bank in order to help researchers conduct research using various iPS cells. RIKEN BRC has received support from the national government (from the Ministry of Education, Culture, Sports, Science and Technology) and has gathered and distributed a large number of iPS cells from and to researchers in and outside Japan. RIKEN BRC has ample resources for preservation of iPS cells and gives lectures on the techniques for handling iPS cells. We would like to consider deposition for your Somatic cells and the iPS cells generated from your Somatic cells to RIKEN BRC so that many researchers can utilize them.

Your privacy will be protected even if the cells are sent from Kyoto University to RIKEN BRC. Your cells can be linked to your personal information only at the Kyoto University (this is called linkable anonymization), whereas RIKEN BRC cannot link the cells with your personal information. RIKEN BRC will distribute your Somatic cells as well as iPS cells

generated from your Somatic cells to researchers and institutions (including pharmaceutical companies) in and outside Japan together with data such as your medical records in accordance with proper procedures and the rules established by the Japanese government. The cells will then be used in research to elucidate the mechanisms of illnesses and assist in the development of new treatments. Neither RIKEN BRC or us will contact you upon distribution of your Somatic cells or iPS cells, but RIKEN BRC will release the cells only to research that has been judged to be appropriate by the specialist committee (Ethics Committee) of the institution to which belong the individual researchers requesting the cells.

Please make sure you understand and consider the meaning of the banking. If you agree to deposition of iPS cells to RIKEN BRC, please choose “I agree” under the section “Deposition of iPS cells to Cell Banks” in the consent form. If you do not agree, please choose “I do not agree.” Your decision to agree or not to deposition of the iPS cells generated from your sample will not affect your regular medical care.

Data generated in this study including genetic information will also be useful for other medical research. Data obtained from you will be, after anonymization (removal of the information including your name and address that can be used to identify you), registered in publicly funded academic databases so that researchers can access the data. We plan to register data from this study in the database of the National Bioscience Database Center (NBDC) of the Japan Science and Technology Agency (JST). JST is an agency under MEXT and promotes and funds scientific research projects in Japan. NBDC was founded in 2011. Data registered in the NBDC will be made accessible to researchers from various fields and will help in the development of new technologies, elucidation of the mechanisms of currently incurable diseases, and discovery of new treatments and prophylactic therapies. If you agree to have your data registered in NBDC, please choose “I agree” under the section “Database registration” in the consent form. If you do not agree, please choose “I do not agree.” Your decision about data registration will not affect your regular medical care.

１７. **Preservation of samples and information after completion of the study**

As already explained, your Somatic cells, the iPS cells generated from the cells and the iPS cell-derived differentiated cells are very valuable. Therefore, these cells along with the information obtained in the course of the study such as your genetic information, DNA or RNA will be preserved at Kyoto University as well as the repositories if the cells are deposited and/or the information is registered for use for research. The preservation period might be a long time even after the completion of this study because such cells and information could lead to new research findings in the future.

１８．Intellectual property generated from this study

Intellectual property (e.g., patents) and intellectual property rights may be generated from the outcomes of the studies conducted using iPS cells generated from your tissue. Such intellectual property rights are not given to the donated sample itself but to the value generated by the work of researchers (research, the use of research outcomes, etc.). Thus, the donor or affiliates of the donor cannot claim the rights by saying, “Because the donor is the one who donated the sample, the intellectual property rights related to the sample should be given to the donor.” For the same reason, if monetary profit is obtained from the intellectual property, the donor cannot claim the right to receive the profit. As a rule, all of the intellectual property is managed by Kyoto University.

１９．Contact information:

If you have questions or concerns about your participation in this study, please feel free to call your study doctor.

If you want to learn more about the study plan, we can show you the study protocol excluding the portions of the protocol where information is confidential due to intellectual property rights, etc.

We cannot show you the study findings at this point because the study outcome has not been completely evaluated.

Your study doctor: ____________
TEL:________________________

Please take your time to read the document carefully until you fully understand the information contained in this informed consent document. After carefully reading this document, if you choose to participate in this study, please sign and date the consent form (Attachment) and give the signed consent form to your study doctor. The original of the signed informed consent form for the present study will be kept by the hospital. You will be given a duplicate of the original informed consent form.

Date:
Doctor who conducted the informed consent discussion: (signature)

Appendix　(20151001)

Investigator and subinvestigators

	Assigned role in the study
Investigator	Oversees the entire study.
Subinvestigator	Responsible for one or more of the following: generation, preservation, management, and distribution of iPS cell cultures; obtaining informed consent (IC), collecting tissue specimens, isolating cells, and conducting disease analysis research using iPS cells.
Physician in charge	Obtains informed consent and collects tissue specimens from patients who are unable to visit Kyoto University Hospital.

	Name	Job title	Role	Obtain IC? (specify the field if yes)
1	Tatsutoshi Nakahata	Part-time Lecturer at the Department of Pediatrics, Graduate School of Medicine, Kyoto University/Professor, Deputy Director at the Center for iPS Cell Research and Application, Kyoto University	Investigator	○ (Pediatrics)
2	Shinya Yamanaka	Director, Professor at the Center for iPS Cell Research and Application, Kyoto University	Subinvestigator	
3	Masato Nakagawa	Lecturer at the Center for iPS Cell Research and Application, Kyoto University	Subinvestigator	
4	Keisuke Okita	Lecturer at the Center for iPS Cell Research and Application, Kyoto University	Subinvestigator	
5	Junya Toguchida	Professor at the Institute for Frontier Medical Sciences/Deputy Director at the Center for iPS Cell Research and Application, Kyoto University	Subinvestigator	○ (Orthopaedic surgery)
6	Makoto Ikeya	Associate Professor at the Center for iPS Cell Research and Application, Kyoto University	Subinvestigator	
7	Toshio Heike	Professor at the Department of Pediatrics, Graduate School of Medicine, Kyoto University	Subinvestigator	○ (Pediatrics)
8	Souichi Adachi	Professor at the Human Health Science, Graduate School of Medicine, Kyoto University	Subinvestigator	○ (Pediatrics)
9	Megumu Saitou	Associate Professor at the Center for iPS Cell Research and Application, Kyoto University	Subinvestigator	○ (Pediatrics)
10	Kazuwa Nakao	Professor at the Innovation Center, Graduate School of Medicine, Kyoto University	Subinvestigator	○ (Diabetes and Clinical Nutrition)
11	Ryosuke Takahashi	Professor at the Department of Neurology, Graduate School of Medicine, Kyoto University	Subinvestigator	○ (Neurology)
12	Haruhisa Inoue	Professor at the Center for iPS Cell Research and Application, Kyoto University	Subinvestigator	○ (Neurology)
13	Shigehiko Suzuki	Professor at the Department of Plastic and Reconstructive Surgery, Graduate School of Medicine, Kyoto University	Subinvestigator	○ (Plastic and reconstructive surgery)
14	Motoko Naitoh	Lecturer at the Department of Plastic and Reconstructive Surgery, Graduate School of Medicine, Kyoto University	Subinvestigator	○ (Plastic and reconstructive surgery)
15	Hiroshi Nakase	Lecturer at the Endoscopy Unit, Kyoto University Hospital	Subinvestigator	○ (Gastroenterology and hepatology)
16	Hiroyuki Marusawa	Lecturer at the Department of Gastroenterology and Hepatology, Graduate School of Medicine, Kyoto University	Subinvestigator	○ (Gastroenterology and hepatology)
17	Shinji Uemoto	Professor at the Division of Hepato-pancreato-biliary Surgery and	Subinvestigator	○ (Hepato-pancreato-biliary

	Name	Job title	Role	Obtain IC? (specify the field if yes)
		Transplantation, Graduate School of Medicine, Kyoto University		surgery and transplantation)
18	Yoshiharu Sakai	Professor at the Department of Gastrointestinal Surgery, Graduate School of Medicine, Kyoto University	Subinvestigator	○ (Gastrointestinal surgery)
19	Shuichi Matsuda	Professor at the Department of Orthopaedic Surgery, Graduate School of Medicine, Kyoto University	Subinvestigator	○ (Orthopaedic surgery)
20	Motoko Yanagita	Professor at the Department of Nephrology, Graduate School of Medicine, Kyoto University	Subinvestigator	○ (Nephrology)
21	Kenji Osafune	Professor at the Center for iPS Cell Research and Application, Kyoto University	Subinvestigator	○ (Nephrology)
22	Michiaki Mishima	Professor at the Department of Respiratory Medicine, Graduate School of Medicine, Kyoto University	Subinvestigator	○ (Respiratory medicine)
23	Isao Ito	Assistant Professor at the Department of Respiratory Medicine, Graduate School of Medicine, Kyoto University	Subinvestigator	○ (Respiratory medicine)
24	Hiroshi Date	Professor at the Department of Thoracic Surgery, Graduate School of Medicine, Kyoto University	Subinvestigator	○ (Thoracic surgery)
25	Takeshi Kimura	Professor at the Department of Cardiovascular Medicine, Graduate School of Medicine, Kyoto University	Subinvestigator	○ (Cardiovascular medicine)
26	Takeru Makiyama	Assistant Professor at the Department of Cardiovascular Medicine, Graduate School of Medicine, Kyoto University	Subinvestigator	○ (Cardiovascular medicine)
27	Yoshinori Yoshida	Lecturer at the Center for iPS Cell Research and Application, Kyoto University	Subinvestigator	○ (Cardiovascular medicine)
28	Kazuhisa Bessho	Professor at the Department of Oral and Maxillofacial Surgery, Graduate School of Medicine, Kyoto University	Subinvestigator	○ (Oral and maxillofacial surgery)
29	Katsu Takahashi	Associate Professor at the Department of Oral and Maxillofacial Surgery, Graduate School of Medicine, Kyoto University	Subinvestigator	○ (Oral and maxillofacial surgery)
30	Tadashi Ikeda	Associate Professor at the Department of Cardiovascular Surgery, Graduate School of Medicine, Kyoto University	Subinvestigator	○ (Cardiovascular surgery)
31	Susumu Miyamoto	Professor at the Department of Neurosurgery, Graduate School of Medicine, Kyoto University	Subinvestigator	○ (Neurosurgery)
32	Akio Koizumi	Professor at the Department of Health and Environmental Sciences, Graduate School of Medicine, Kyoto University	Subinvestigator	
33	Shigeru Hirano	Associate Professor at the Department of Otolaryngology, Head and Neck Surgery, Graduate School of Medicine, Kyoto University	Subinvestigator	○ (Otolaryngology)
34	Shinichiro Kitajiri	Assistant Professor at the Department of Otolaryngology, Head and Neck Surgery, Graduate School of Medicine, Kyoto University	Subinvestigator	○ (Otolaryngology)
35	Kenji Kabashima	Professor at the Department of Dermatology, Graduate School of Medicine, Kyoto University	Subinvestigator	○ (Dermatology)
36	Osamu Ogawa	Professor at the Department of Urology, Graduate School of Medicine, Kyoto University	Subinvestigator	○ (Urology)

	Name	Job title	Role	Obtain IC?<br>(specify the field if yes)
37	Eijiro Nakamura	Associate Professor at the Medical Innovation Center, Graduate School of Medicine, Kyoto University	Subinvestigator	○ (Urology)
38	Masakatsu Sone	Lecturer at the Department of Diabetes and Clinical Nutrition, Graduate School of Medicine, Kyoto University	Subinvestigator	○ (Diabetes and Clinical Nutrition)
39	Junji Fujikura	Assistant Professor at the Department of Diabetes and Clinical Nutrition, Graduate School of Medicine, Kyoto University	Subinvestigator	○ (Diabetes and Clinical Nutrition)
40	Ryuta Nishikomori	Associate Professor at the Department of Pediatrics, Graduate School of Medicine, Kyoto University	Subinvestigator	○ (Pediatrics)
41	Tomonari Awaya	Assistant Professor at the Department of Pediatrics, Graduate School of Medicine, Kyoto University	Subinvestigator	○ (Pediatrics)
42	Katsutsugu Umeda	Assistant Professor at the Department of Pediatrics, Graduate School of Medicine, Kyoto University	Subinvestigator	○ (Pediatrics)
43	Shiro Baba	Assistant Professor at the Department of Pediatrics, Graduate School of Medicine, Kyoto University	Subinvestigator	○ (Pediatrics)
44	Akifumi Takaori	Professor at the Department of Hematology and Oncology, Graduate School of Medicine, Kyoto University	Subinvestigator	○ (Hematology and oncology)
45	Hiroshi Kawabata	Lecturer at the Department of Hematology and Oncology, Graduate School of Medicine, Kyoto University	Subinvestigator	○ (Hematology and oncology)
46	Isao Asaka	Professor at the Center for iPS Cell Research and Application, Kyoto University	Subinvestigator	
47	Yasuhiro Yamada	Professor at the Center for iPS Cell Research and Application, Kyoto University	Subinvestigator	
48	Hidetoshi Sakurai	Associate Professor at the Center for iPS Cell Research and Application, Kyoto University	Subinvestigator	○ (Nephrology)
49	Takafumi Kimura	Professor at the Center for iPS Cell Research and Application, Kyoto University	Subinvestigator	○ (iPS cell application development)
50	Jun Takahashi	Professor at the Center for iPS Cell Research and Application, Kyoto University	Subinvestigator	○ (Neurosurgery)
51	Jun Yamashita	Professor at the Center for iPS Cell Research and Application, Kyoto University	Subinvestigator	○ (Cardiovascular medicine)
52	Akira Niwa	Assistant Professor at the Center for iPS Cell Research and Application, Kyoto University	Subinvestigator	
53	Asuka Morizane	Assistant Professor at the Center for iPS Cell Research and Application, Kyoto University	Subinvestigator	
54	Daisuke Doi	Researcher at the Center for iPS Cell Research and Application, Kyoto University	Subinvestigator	○ (Neurosurgery)
55	Tetsuhiro Kikuchi	Researcher at the Center for iPS Cell Research and Application, Kyoto University	Subinvestigator	
56	Kazuhisa Chonabayashi	Researcher at the Center for iPS Cell Research and Application, Kyoto University	Subinvestigator	
57	Noriyuki Tsumaki	Professor at the Center for iPS Cell Research and Application, Kyoto University	Subinvestigator	○ (Orthopaedic surgery)
58	Yoshiya Kawaguchi	Professor at the Center for iPS Cell Research and Application, Kyoto University	Subinvestigator	○ (Hepato-pancreato-biliary surgery and transplantation)
59	Koji Eto	Professor at the Center for iPS Cell Research and Application, Kyoto University	Subinvestigator	○ (Cardiovascular medicine)
60	Toshiya Murai	Professor at the Department of Psychiatry, Graduate School of Medicine, Kyoto University	Subinvestigator	○ (Psychiatry)

	Name	Job title	Role	Obtain IC? (specify the field if yes)
61	Masaaki Hazama	Assistant Professor at the Department of Psychiatry, Graduate School of Medicine, Kyoto University	Subinvestigator	○ (Psychiatry)
62	Ikuo Konishi	Professor at the Department of Gynecology and Obstetrics, Graduate School of Medicine, Kyoto University	Subinvestigator	○ (Gynecology and obstetrics)
63	Junzo Hamanishi	Assistant Professor at the Department of Gynecology and Obstetrics, Graduate School of Medicine, Kyoto University	Subinvestigator	○ (Gynecology and obstetrics)
64	Tsuneyo Mimori	Professor at the Department of Rheumatology and Clinical Immunology, Graduate School of Medicine, Kyoto University	Subinvestigator	○ (Rheumatology and clinical immunology)
65	Koichiro Ohmura	Associate Professor at the Department of Rheumatology and Clinical Immunology, Graduate School of Medicine, Kyoto University	Subinvestigator	○ (Rheumatology and clinical immunology)
66	Hajime Yoshifuji	Assistant Professor at the Department of Rheumatology and Clinical Immunology, Graduate School of Medicine, Kyoto University	Subinvestigator	○ (Rheumatology and clinical immunology)
67	Nobuya Inagaki	Professor at the Department of Diabetes and Clinical Nutrition, Graduate School of Medicine, Kyoto University	Subinvestigator	○ (Diabetes and clinical nutrition)
68	Daisuke Tanaka	Assistant Professor at the Department of Diabetes and Clinical Nutrition, Graduate School of Medicine, Kyoto University	Subinvestigator	○ (Diabetes and clinical nutrition)
69	Nagahisa Yoshimura	Professor at the Department of Ophthalmology and Visual Sciences, Graduate School of Medicine, Kyoto University	Subinvestigator	○ (Ophthalmology and visual sciences)
70	Akio Ooishi	Assistant Professor at the Department of Ophthalmology and Visual Sciences, Graduate School of Medicine, Kyoto University	Subinvestigator	○ (Ophthalmology and visual sciences)
71	Taira Maekawa	Professor at the Department of Transfusion Medicine and Cell Therapy, Kyoto University Hospital	Subinvestigator	○ (Transfusion medicine and cell therapy)
72	Hideyo Hirai	Assistant Professor at the Department of Transfusion Medicine and Cell Therapy, Kyoto University Hospital	Subinvestigator	○ (Transfusion medicine and cell therapy)
73	Yasuo Miura	Assistant Professor at the Department of Transfusion Medicine and Cell Therapy, Kyoto University Hospital	Subinvestigator	○ (Transfusion medicine and cell therapy)
74	Hanako Ikeda	Associate Professor at the Department of Ophthalmology and Visual Sciences, Graduate School of Medicine, Kyoto University	Subinvestigator	○ (Ophthalmology and visual sciences)
75	Shin Kaneko	Associate Professor at the Center for iPS Cell Research and Application, Kyoto University	Subinvestigator	
76	WOLTJEN Knut	Associate Professor at the Hakubi Center/the Center for iPS Cell Research and Application, Kyoto University	Subinvestigator	
77	Takeshi Okamoto	Lecturer at the Department of Orthopaedic Surgery, Graduate School of Medicine, Kyoto University	Subinvestigator	○ (Orthopaedic surgery)
78	Hirofumi Yamashita	Assistant Professor at the Department of Neurology, Kyoto University Hospital	Subinvestigator	○ (Neurology)
79	Hodaka Yamakado	Assistant Professor at the Department of Neurology, Kyoto University Hospital	Subinvestigator	○ (Neurology)
80	Mitsujiro Osawa	Assistant Professor at the Center for iPS Cell Research and Application, Kyoto University	Subinvestigator	

	Name	Job title	Role	Obtain IC? (specify the field if yes)
81	Akitsu Hotta	Assistant Professor at the Center for iPS Cell Research and Application/the Institute for Integrated Cell-Material Sciences, Kyoto University	Subinvestigator	
82	Minoru Matsuura	Assistant Professor at the Department of Gastroenterology and Hepatology, Graduate School of Medicine, Kyoto University	Subinvestigator	○ (Gastroenterology and hepatology)
83	Genichi Sugihara	Assistant Professor at the Department of Psychiatry, ,Kyoto University Hospital	Subinvestigator	○ (Psychiatry)
84	Mitinori Saitou	Professor at the Anatomy and Cell Biology, Graduate School of Medicine and Faculty of Medicine, Kyoto University	Subinvestigator	
85	Takahito Wada	Associate Professor at the Medical Ethics and Medical Genetics, Graduate School of Medicine and Faculty of Medicine, Kyoto University	Subinvestigator	○（Clinical Genetics Unit）
86	Sihori Yokobayashi	Assistant Professor at the Center for iPS Cell Research and Application	Subinvestigator	
87	Takeshi Sakurai	Associate Professor at the Medical Inovation Center, Kyoto University	Subinvestigator	
88	Akira Ohta	Researcher at the Center for iPS Cell Research and Application, Kyoto University	Subinvestigator	
89	Yohei Nishi	Researcher at the Center for iPS Cell Research and Application, Kyoto University	Subinvestigator	
90	Toshio Kitawaki	Assistant Professor at the Department of Hematology and Oncology, Graduate School of Medicine, Kyoto University	Subinvestigator	○ (Hematology and oncology)
91	Shigeo Muro	Lecturer at the Department of Respiratory Medicine, Graduate School of Medicine, Kyoto University	Subinvestigator	○ (Respiratory medicine)
92	Hisako Matsumoto	Lecturer (Hospital) at the Department of Respiratory Medicine, Graduate School of Medicine, Kyoto University	Subinvestigator	○ (Respiratory medicine)
93	Shimpei Gotoh	Assistant Professor at the Department of Respiratory Medicine, Graduate School of Medicine, Kyoto University	Subinvestigator	○ (Respiratory medicine)
94	Eigaku Kim	Assistant Professor at the Department of Respiratory Medicine, Graduate School of Medicine, Kyoto University	Subinvestigator	○ (Respiratory medicine)

Appendix 2　(20151001)

The list of specimen collection centers

The subinvestigators who are assigned to obtain informed consent as in Appendix1 or the attending physician at the centers will obtain informed consent.

	Institution
1	Department of Dermatology, Chiba University School of Medicine
2	Division of Respirology, Neurology, and Rheumatology, Kurume University School of Medicine
3	Laboratory of Immune Regulation, Wakayama Medical University
4	Department of Pediatrics, Wakayama Medical University
5	Division of Neurology, Hyogo College of Medicine
6	Imamura Bun-in Hospital
7	Kitano Hospital, the Tazuke Kofukai Medical Research Institute
8	Nishi-Kobe Medical Center
9	Department of Neurology, School of Medicine, Gunma University
10	Miyagi Children's Hospital
11	National Hospital Organization Sagamihara National Hospital
12	Tokyo Metropolitan Neurological Hospital
13	Department of Neurology, Departmento of Pediatrics, Jichi Medical University
14	Okitama Public General Hospital
15	Seirei Hamamatsu General Hospital
16	Tokai University
17	Kawasaki Medical School
18	University of Tsukuba
19	The University of Tokushima
20	National Hospital Organization Utano Hospital
21	Shizuoka Institute of Epilepsy and Neurological Disorders
22	The Jikei University School of Medicine
23	Department of Respiratory Medicine, Juntendo University Faculty of Medicine
24	Shizuoka Children's Hospital
25	Department of Pediatric Nephrology, School of Medicine, Tokyo Women's Medical University
26	Department of Pediatrics, Graduate School of Medicine, Gifu University
27	Department of Pediatrics, National Hospital Organization Minami-Kyoto Hospital
28	Department of Neurology, Fukuoka University
29	Department of Dermatology, Wakayama Medical University
30	Department of Orthopaedics, Shiga Medical Center for Children
31	Department of Regulatory Medicine for Thrombosis, Nara Medical University
32	Ehime Proteo-Medicine Research Center, Ehime University
33	Center for Diabetes & Endocrinology, Kitano Hospital, the Tazuke Kofukai Medical Research Institute
34	Department of Pediatric Surgery, Department of Orthopaedic Surgery, Faculty of Medical Sciences, Kyushu University
35	Department of Neurology, Tokyo Metropolitan Neurological Hospital
36	Minami Kyushu National Hospital
37	Department of Pediatrics, Kobe University
38	Nagano Children’s Hospital
39	National Cerebral and Cardiovascular Center
40	Gifu University Hospital
41	Kawasaki Medical School Hospital
42	University of Yamanashi
43	Yamagata University
44	Shinshu University
45	Department of Pediatrics, Osaka University
46	Atomic Bomb Disease Institute, Nagasaki University
47	Dokkyo Medical University
48	Fukuchiyama City Hospital

	Institution
49	Nihonkai General Hospital
50	Department of Neurosurgery, The University of Tokyo
51	Carlo Besta Neurological Institute
52	Department of Neurology, Osaka University
53	Research Institute for Diseases of Old Age, Juntendo University
54	Department of Neurology, Toho University Omori Medical Center
55	Department of Neurology, Okayama University Graduate School of Medicine, Dentistry and Pharmaceutical Sciences
56	Department of Pediatrics, Tokyo Women’s Medical University
57	Institute of Medical Genetics, Tokyo Women’s Medical University
58	Division of Nephrology, Department of Medicine, Kurume University School of Medicine
59	Department of Medical Genetics, Osaka Medical Center and Research Institute for Maternal and Child Health
60	Department of Brain and Neurosciences, Faculty of Medicine, Tottori University
61	Kanagawa Children’s Medical Center
62	Department of Gastroenterology, Tohoku University
63	Department of Orthopaedic Surgery, Keio University School of Medicine, Keio University
64	Department of Orthopaedic Surgery, Kurashiki Central Hospital
65	Department of Neurology, Osaka City University Graduate School of Medicine, Osaka City University
66	Department of Pediatrics, University Hospital of Medicine, Tokyo Medical And Dental University

20151005 (Supplement 1)

Informed Consent Form
(for the General Study)

This consent form applies to patients and healthy family members of the patients

[Name of the person to whom the consent is given (subinvestigator) if the informed consent discussion is held in Kyoto University] or
[Name of the head of the medical institution, etc. if the informed consent discussion is held outside the Kyoto University]

Study title: The Generation of Human Disease-Specific iPS Cells and the Use of Such iPS Cells for Disease Analysis

I have been given information about this study, in which a sample of my body tissue will be taken to generate iPS cells. The following items regarding the study have been explained to me by the study doctor using the informed consent document. I volunteer to take part in this study.

1. That participation is voluntary and I am free to withdraw my consent
2. That the plan of the study has been approved by the Ethics Committee
3. The purpose of the study
4. The administrative organization of the study
5. The study procedures
6. The sampling of body tissue
7. The use of iPS cells generated in this study
8. Gene analysis
9. The protection of my personal information
10. Provision of specimens to third-party research institutions
11. iPS cell banking and database registration
12. How I can learn more about the study plan
13. Publication of study data
14. The expected benefits and risks of participating in the study
15. Preservation of samples and information after completion of the study
16. Intellectual property rights generated from this study
17. Costs
18. Contact information
19. That iPS cells generated from a healthy member of the patient will be used to compare with various diseases
20. Do you want to receive the result of screening test for virus infection?
    Yes　　　No
21. Do you agree to the provision of your somatic cells to third-party research institutions other than the Center for iPS Cell Research and Application, Kyoto University; the Institute for Frontier Medical Sciences, Kyoto University; and Graduate School of Medicine, Kyoto University?
    I agree.　　　I do not agree.
22. Do you agree to provision of the iPS cells to third-party institutions?
    I agree.　　　I do not agree.
23. Do you agree to deposition of your somatic cells to the iPS cell banking project?
    I agree.　　　I do not agree.
24. Do you agree to deposition of the iPS cells to the iPS cell banking project?
    I agree.　　　I do not agree.
25. Do you agree to have your data registered in a public database?
    I agree.　　　I do not agree.

Patient (Name)

Date of consent:

Donor	(Signature)
Legal representative	(Signature)
	(Relationship of the legal representative to the patient)

I confirm that I have given the donor detailed information about the study and that the donor has consented to the study on the basis of his/her free will.

Institution (Name)/ Department (Name)

Doctor who conducted the informed consent discussion (Name)

The hospital will keep the original of the signed form, and a copy will be given to the donor.

20151001 (Supplement 2)

Informed Consent Form[for the General Study]

This consent form applies to healthy volunteers.

[Name of the person to whom the consent is given (subinvestigator) if the informed consent discussion is held in Kyoto University] or
[Name of the head of the medical institution, etc. if the informed consent discussion is held outside the Kyoto University]

Study title:
The Generation of Human Disease-Specific iPS Cells and the Use of Such iPS Cells for Disease Analysis

I have been given information about this study, in which a sample of my body tissue will be taken to generate iPS cells. The following items regarding the study have been explained to me by the study doctor using the informed consent document. I volunteer to take part in this study.

- That the plan of the study has been approved by the Ethics Committee
- The study procedures
- The administrative organization of the study
- The expected benefits and risks of participating in the study
- That my participation is voluntary and I am free to withdraw my consent
- That my personal information will be strictly protected
- Gene analysis
- Contribution to iPS cell banking project and database registration
- Contact information
- The purpose of the study
- The sampling of body tissue
- That an interview about my medical history is required
- Costs
- That the iPS cells generated in this study will be used appropriately
- That the study data will be published after removing my personal information
- Provision of specimens to third-party research institutions
- Preservation of samples and information after completion of the study
- Intellectual property rights generated from this study

* Please check √ the box that applies.

Do you agree to take medical history interview?	(☐ I agree	☐ I do not agree)
Do you agree to provision of your somatic cells to third-party research institutions?	(☐ I agree	☐ I do not agree)
Do you agree to provision of the iPS cells to third-party research institutions?	(☐ I agree	☐ I do not agree)
Do you agree to deposition of your somatic cells to the cell banking project?	(☐ I agree	☐ I do not agree)
Do you agree to deposition of the iPS cells to the cell banking project?	(☐ I agree	☐ I do not agree)
Do you agree to have your data registered in a public database?	(☐ I agree	☐ I do not agree)

Date of consent:

Donor (Signature)

Legal representative (Signature)

(Relationship of the legal representative to the patient)

I confirm that I have given the donor detailed information about the study, including purpose, details, and protection of personal information, and the donor has consented to the study on the basis of his/her free will.

Date:

Institution (Name)/ Department (Name):

Doctor who conducted the informed consent discussion (Name)

The hospital will keep the original of the signed form, and a copy will be given to the donor.
