# Supplementary material for: In vitro methods to ensure absence of residual undifferentiated human induced pluripotent stem cells intermingled in induced nephron progenitor cells
Source: PLoS One. 2022 Nov 15;17(11):e0275600. doi: 10.1371/journal.pone.0275600 (PMC9665373; doi:10.1371/journal.pone.0275600)
Supplement: S1 File — (ZIP) [file pone.0275600.s021.zip › S1_documents/585A1_ICF_hito_g259_20160407.pdf]

Information on Genetic Analysis Study Using Human Disease-Specific iPS Cells

<What is a gene?>

"Heredity" is "the passing on of traits from parents to children." "Traits" include facial/body features, and susceptibility to certain diseases. The features of the human body are determined by heredity and the environment in which the individual grows up. However, heredity plays an important role as the basis for development of the body and mind. In Japanese, when the kanji "Ko (children)" is added to the kanji "Iden (heredity)," the combined kanji becomes a scientific term for "small units that determine heredity." In most organisms, the building blocks of genes are molecules called DNA. DNA is a chain consisting of 4 bases: A, T, G, and C. A gene comprises many of these bases that are linked together.

One cell includes tens of thousands of genes that are scattered throughout the cell. All the genetic information is collectively referred to as the "genome." The human body comprises about 60 trillion cells, and each cell includes all genes.

There are two major roles of genes. The first is to act as an accurate "blueprint for the body." It all starts from one fertilized cell. As the cell divides and increases in number, each cell develops into a particular type of cell; for example, one cell develops into an eye cell, and another into an intestinal cell. The number of cells increases up to about 60 trillion in a mature adult. The second role is to "preserve the species." The human race has been maintained in its present from our most distant ancestors up to the present, thanks to genes.

<Genes and disease>

Nearly all diseases occur as a result of the interactions among an individual's inherited biological make-up (genetic predisposition), pathogens, and the influence of lifestyle (environmental factors). Genetic predisposition and environmental factors may be intertwined in the pathology of some diseases; whereas, in other diseases, either genetic predisposition or environmental factors can be identified as the fundamental cause. Moreover, in some cases, disease occurs due to a combination of two or more genetic dispositions. Nevertheless, multiple factors including genetic predisposition (genetic differences) are involved in the onset of disease.

<Participation in the genetic diagnostic study>

This study will be conducted to search for the genes that may be involved in the onset of disease or that may influence an individual's response to a drug. Another purpose of the study is to investigate the genes suspected, for particular reasons, to be linked to a disease: the structure and function of the genes will be analyzed to determine if the genes are truly related to the disease. If the genotype of the patient is determined, the result can be used to determine the genetic polymorphism of blood relatives. This may raise concerns within the family. Our hospital offers a genetic counseling service in order to help relieve the anxiety the patient and family members may feel and address other issues.

The following sections give you the information related to the genetic diagnostic study, including the benefits and risks of participating in the study. Our intention is to explain the study using easy-to-understand language. If you have a question, please feel free to ask at any time. Please make sure you fully understand the information contained in this document before making a decision. If you choose to participate in this study and give your permission for the genes of the iPS cells generated from your body tissue to be analyzed, please fill out the information in the consent form. By signing the

consent form, you will have indicated that you understand the information and that you give your consent to participate in this study.

<Information to help you decide whether or not to participate in this genetic diagnostic study>
(1) You are free to choose to participate or not to participate in this genetic diagnostic study. If you change your mind later, you may withdraw your consent at any time. Your participation is voluntary. You are under no obligation to participate in this study. Your decision to participate or not will have no influence on your current and future relationship with our hospital. We will always treat you in your best interests regardless of your decision.
If you consent to the study and change your mind later, you may withdraw your consent simply by writing to us. You do not have to explain the reason. There is no penalty or loss or benefits for withdrawing from the study. If you withdraw your consent, the results of your genetic analysis will be destroyed and your medical record will also not be used for the study from that time on. Note that, however, recovery and disposal of your specimens may sometimes be difficult at the time when you withdraw your consent; for example, when the study using your specimens has made certain progress, a paper including data from the study has been published, or data from the study have been used by other institutions using the cell bank (this will be described later in this leaflet). In such cases, use of your specimens and/or the data obtained from your specimens may continue despite your withdrawal of consent.
Two originals of the signed informed consent form for the present study will be made. One of the originals will be kept by the hospital, and the other will be given to you.

(2) The plan of genetic diagnostic study is presented below.
The plan of this genetic analysis study has been reviewed by the Medical Ethics Committee in the Graduate School of Medicine, Kyoto University and Kyoto University Hospital, and approved by the head of the research institution conducting the study.

Study title:	Genetic Analysis Study Using Human Disease-Specific iPS Cells
Name of research institution	Graduate School of Medicine, Kyoto University/Center for iPS Cell Research and Application
Investigator (Name, Job title)	Study director:<br>Tatsutoshi Nakahata (Part-time Lecturer at the Department of Pediatrics, Graduate School of Medicine, Kyoto University/Department of Pediatrics at Kyoto University Hospital, Deputy Director at the Center for iPS Cell Research and Application, Kyoto University)<br><br>Subinvestigators:<br>Toshio Heike (Professor at the Department of Pediatrics, Graduate School of Medicine, Kyoto University)<br>Ryuta Nishikomori (Associate Professor at the Department of Pediatrics, Graduate School of Medicine, Kyoto University)<br>Tomonari Awaya (Assistant Professor at the Department of Pediatrics, Graduate School of Medicine, Kyoto University)<br>Katsutsugu Umeda (Assistant Professor at the Department of Pediatrics, Graduate School of Medicine, Kyoto University)<br>Shiro Baba (Assistant Professor at the Department of Pediatrics, Graduate

	School of Medicine, Kyoto University)<br>Souichi Adachi (Professor at the Human Health Science, Graduate School of Medicine, Kyoto University)<br>Masakatsu Sone (Lecturer at the Department of Diabetes and Clinical Nutrition, Graduate School of Medicine, Kyoto University)<br>Junji Fujikura (Assistant Professor at the Department of Diabetes and Clinical Nutrition, Graduate School of Medicine, Kyoto University)<br>Ryosuke Takahashi (Professor at the Department of Neurology, Graduate School of Medicine, Kyoto University)<br>Shigehiko Suzuki (Professor at the Department of Plastic and Reconstructive Surgery, Graduate School of Medicine, Kyoto University)<br>Motoko Naitoh (Lecturer at the Department of Plastic and Reconstructive Surgery, Graduate School of Medicine, Kyoto University)<br>Hiroshi Nakase (Lecturer at the Endoscopy Unit, Kyoto University Hospital)<br>Hiroyuki Marusawa (Lecturer at the Department of Gastroenterology and Hepatology, Graduate School of Medicine, Kyoto University)<br>Minoru Matsuura (Assistant Professor at the Department of Gastroenterology and Hepatology, Graduate School of Medicine, Kyoto University)<br>Shinji Uemoto (Professor at the Division of Hepato-pancreato-biliary Surgery and Transplantation, Department of Surgery, Graduate School of Medicine, Kyoto University)<br>Yoshiharu Sakai (Professor at the Department of Gastrointestinal Surgery, Graduate School of Medicine, Kyoto University)<br>Shuichi Matsuda (Professor at the Department of Orthopaedic Surgery, Graduate School of Medicine, Kyoto University)<br>Takeshi Okamoto (Lecturer at the Department of Orthopaedic Surgery, Graduate School of Medicine, Kyoto University)<br>Motoko Yanagita (Professor at the Department of Nephrology, Graduate School of Medicine, Kyoto University)<br>Michiaki Mishima (Professor at the Department of Respiratory Medicine, Graduate School of Medicine, Kyoto University)<br>Isao Ito (Assistant Professor at the Department of Respiratory Medicine, Graduate School of Medicine, Kyoto University)<br>Hiroshi Date (Professor at the Department of Thoracic Surgery, Graduate School of Medicine, Kyoto University)<br>Takeshi Kimura (Professor at the Department of Cardiovascular Medicine, Graduate School of Medicine, Kyoto University)<br>Takeru Makiyama (Assistant Professor at the Department of Cardiovascular Medicine, Graduate School of Medicine, Kyoto University)<br>Kazuhisa Bessho (Professor at the Department of Oral and Maxillofacial Surgery, Graduate School of Medicine, Kyoto University)<br>Katsu Takahashi (Associate Professor at the Department of Oral and Maxillofacial Surgery, Graduate School of Medicine, Kyoto University)<br>Tadashi Ikeda (Associate Professor at the Department of Cardiovascular Surgery, Graduate School of Medicine, Kyoto University)<br>Susumu Miyamoto (Professor at the Department of Neurosurgery, Graduate School of Medicine, Kyoto University)<br>Akio Koizumi (Professor at the Department of Health and Environmental Sciences, Graduate School of Medicine, Kyoto University)<br>Shigeru Hirano (Associate Professor at the Department of Otolaryngology, Head and Neck Surgery, Graduate School of Medicine, Kyoto University)<br>Shinichiro Kitajiri (Assistant Professor at the Department of Otolaryngology, Head and Neck Surgery, Graduate School of Medicine, Kyoto University)

	Kenji Kabashima (Professor at the Department of Dermatology, Graduate School of Medicine, Kyoto University)<br>Osamu Ogawa (Professor at the Department of Urology, Graduate School of Medicine, Kyoto University)<br>Kazuwa Nakao (Professor at the Medical Innovation Center, Graduate School of Medicine, Kyoto University)<br>Eijiro Nakamura (Associate Professor at the Medical Innovation Center, Graduate School of Medicine, Kyoto University)<br>Akifumi Takaori (Professor at the Department of Hematology and Oncology, Graduate School of Medicine, Kyoto University)<br>Hiroshi Kawabata (Lecturer at the Department of Hematology and Oncology, Graduate School of Medicine, Kyoto University)<br>Toshio Kitawaki (Assistant Professor at the Department of Hematology and Oncology, Graduate School of Medicine, Kyoto University)<br>Toshiya Murai (Professor at the Department of Psychiatry, Graduate School of Medicine, Kyoto University)<br>Masaaki Hazama (Assistant Professor at the Department of Psychiatry, Graduate School of Medicine, Kyoto University)<br>Genichi Sugihara (Assistant Professor at the Department of Psychiatry, ,Kyoto University Hospital)<br>Ikuo Konishi (Professor at the Department of Gynecology and Obstetrics, Graduate School of Medicine, Kyoto University)<br>Junzo Hamanishi (Assistant Professor at the Department of Gynecology and Obstetrics, Graduate School of Medicine, Kyoto University)<br>Tsuneyo Mimori (Professor at the Department of Rheumatology and Clinical Immunology, Graduate School of Medicine, Kyoto University)<br>Koichiro Ohmura (Associate Professor at the Department of Rheumatology and Clinical Immunology, Graduate School of Medicine, Kyoto University)<br>Hajime Yoshifuji (Assistant Professor at the Department of Rheumatology and Clinical Immunology, Graduate School of Medicine, Kyoto University)<br>Nobuya Inagaki (Professor at the Department of Diabetes and Clinical Nutrition, Graduate School of Medicine, Kyoto University)<br>Daisuke Tanaka (Assistant Professor at the Department of Diabetes and Clinical Nutrition, Graduate School of Medicine, Kyoto University)<br>Nagahisa Yoshimura (Professor at the Department of Ophthalmology and Visual Sciences, Graduate School of Medicine, Kyoto University)<br>Akio Ooishi (Assistant Professor at the Department of Ophthalmology and Visual Sciences, Graduate School of Medicine, Kyoto University)<br>Hanako Ikeda (Associate Professor at the Department of Ophthalmology and Visual Sciences, Graduate School of Medicine, Kyoto University)<br>Taira Maekawa (Professor at the Department of Transfusion Medicine and Cell Therapy, Kyoto University Hospital)<br>Hideyo Hirai (Assistant Professor at the Department of Transfusion Medicine and Cell Therapy, Kyoto University Hospital)<br>Yasuo Miura (Assistant Professor at the Department of Transfusion Medicine and Cell Therapy, Kyoto University Hospital)<br>Hirofumi Yamashita (Assistant Professor at the Department of Neurology, Kyoto University Hospital)<br>Hodaka Yamakado (Assistant Professor at the Department of Neurology, Kyoto University Hospital)<br>Mitinori Saitou (Professor at Anatomy and Cell Biology, Kyoto Graduate School of Medicine and Faculty of Medicine, Kyoto University)<br>Takahito Wada (Associate Professor at the Medical Ethics and Medical

	Genetics, Graduate School of Medicine and Faculty of Medicine, Kyoto University)<br>Takeshi Sakurai(Associate Professor at the Medical Inovation Center, Kyoto University)<br>Seishi Ogawa (Professor at Pathology and Tumor Biology, Kyoto University Graduate School of Medicine)<br>Shigeo Muro (Lecturer at the Department of Respiratory Medicine, Graduate School of Medicine, Kyoto University)<br>Hisako Matsumoto (Lecturer (Hospital) at the Department of Respiratory Medicine, Graduate School of Medicine, Kyoto University)<br>Shimpei Gotoh (Assistant Professor at the Department of Respiratory Medicine, Graduate School of Medicine, Kyoto University)<br>Eigaku Kim (Assistant Professor at the Department of Respiratory Medicine, Graduate School of Medicine, Kyoto University)
Name of collaborating research institutions, and the name of the representative	Shinya Yamanaka (Director, Professor at the Center for iPS Cell Research and Application, Kyoto University)<br>Junya Toguchida (Professor at the Institute for Frontier Medical Sciences, Kyoto University/Deputy Director at the Center for iPS Cell Research and Application)<br>Haruhisa Inoue (Professor at the Center for iPS Cell Research and Application, Kyoto University)<br>Megumu Saito(Associate Professor at the Center for iPS Cell Research and Application, Kyoto University)<br>Kenji Osafune (Professor at the Center for iPS Cell Research and Application, Kyoto University)<br>Isao Asaka (Professor at the Center for iPS Cell Research and Application, Kyoto University)<br>Yasuhiro Yamada (Professor at the Institute for Integrated Cell-Material Sciences/ Center for iPS Cell Research and Application, Kyoto University)<br>Hidetoshi Sakurai (Associate Professor at the Center for iPS Cell Research and Application, Kyoto University)<br>Takafumi Kimura (Professor at the Center for iPS Cell Research and Application, Kyoto University)<br>Jun Takahashi (Professor at the Center for iPS Cell Research and Application, Kyoto University)<br>Jun Yamashita (Professor at the Center for iPS Cell Research and Application, Kyoto University)<br>Akira Niwa (Assistant Professor at the Center for iPS Cell Research and Application, Kyoto University)<br>Asuka Morizane (Assistant Professor at the Center for iPS Cell Research and Application, Kyoto University)<br>Daisuke Doi (Researcher at the Center for iPS Cell Research and Application, Kyoto University)<br>Tetsuhiro Kikuchi (Researcher at the Center for iPS Cell Research and Application, Kyoto University)<br>Kazuhisa Chonabayashi (Researcher at the Center for iPS Cell Research and Application, Kyoto University)<br>Noriyuki Tsumaki (Professor at the Center for iPS Cell Research and Application, Kyoto University)<br>Yoshiya Kawaguchi (Professor at the Center for iPS Cell Research and

	Application, Kyoto University)<br>Koji Eto (Professor at the Center for iPS Cell Research and Application, Kyoto University)<br>Yoshinori Yoshida (Lecturer at the Center for iPS Cell Research and Application, Kyoto University)<br>Shin Kaneko (Associate Professor at the Center for iPS Cell Research and Application, Kyoto University)<br>Makoto Ikeya (Associate Professor at the Center for iPS Cell Research and Application, Kyoto University)<br>WOLTJEN Knut (Associate Professor at the Hakubi Center/the Center for iPS Cell Research and Application, Kyoto University)<br>Mitsujiro Osawa (Assistant Professor at the Center for iPS Cell Research and Application, Kyoto University)<br>Akitsu Hotta (Assistant Professor at the Center for iPS Cell Research and Application/the Institute for Integrated Cell-Material Sciences, Kyoto University)<br>Sihori Yokobayashi (Assistant Professor at the Center for iPS Cell Research and Application, Kyoto University)<br>Akira Ohta (Researcher at the Center for iPS Cell Research and Application, Kyoto University)<br>Yohei Nishi (Researcher at the Center for iPS Cell Research and Application, Kyoto University)<br>Masato Nakagawa (Lecturer at the Center for iPS Cell Research and Application, Kyoto University)<br>Keisuke Okita (Lecturer at the Center for iPS Cell Research and Application, Kyoto University)<br><br>RIKEN<br>Yoshihide Hayashizaki (Director at the Omics Science Center, RIKEN)<br>Other collaborating research institutions<br>Eri Muso (Kitano Hospital, the Tazuke Kofukai Medical Research Institute)<br>Yoshiyuki Hamamoto (Kitano Hospital, the Tazuke Kofukai Medical Research Institute)<br>Takashi Fukaya (Nishi-Kobe Medical Center)<br>Koichi Okamoto (Department of Neurology, School of Medicine, Gunma University)<br>Masuei Imaizumi (Miyagi Children’s Hospital)<br>Kazuko Hasegawa (National Hospital Organization Sagamihara National Hospital)<br>Akihiro Kawata (Tokyo Metropolitan Neurological Hospital)<br>Mitsuya Morita (Jichi Medical University)<br>Takaki Akahane (Okitama Public General Hospital)<br>Tsuyoshi Uchiyama (Seirei Hamamatsu General Hospital)<br>Miharu Yabe (Tokai University)<br>Yutaka Ohsawa (Kawasaki Medical School)<br>Akira Tamaoka, Takashi Asada (University of Tsukuba)<br>Ryuji Kaji (The University of Tokushima)<br>Hideyuki Sawada (National Hospital Organization Utano Hospital)<br>Yushi Inoue (Shizuoka Institute of Epilepsy and Neurological Disorders)<br>Michihiro Yoshimura (The Jikei University School of Medicine)<br>Shigeharu Oh (Shizuoka Children's Hospital)<br>Kuniaki Seyama (Juntendo University hospital)<br>Motoshi Hattori (Department of Pediatric Nephrology, School of Medicine,

	Tokyo Women's Medical University)<br>Naomi Kondo (Department of Pediatrics, Graduate School of Medicine, Gifu University)<br>Takeshi Miyanomae (Department of Pediatrics, National Hospital Organization Minami-Kyoto Hospital)<br>Yoshio Tsuboi (Department of Neurology, Fukuoka University)<br>Nobuo Kanazawa (Department of Dermatology, Wakayama Medical University)<br>Tohru Futami (Department of Orthopaedics, Shiga Medical Center for Children)<br>Hideto Matsui (Department of Regulatory Medicine for Thrombosis, Nara Medical University)<br>Tetsuro Miki (Ehime Proteo-Medicine Research Center, Ehime University)<br>Shoji Tsuji (Neurology, Graduate School of Medicine and Faculty of Medicine, the University of Tokyo)<br>Haruhiko Akiyama (Psychogeriatric Research Team, Tokyo Institute of Psychiatry)<br>Naotomo Kambe (Department of Dermatology, Chiba University School of Medicine)<br>Norihiro Nishimoto (Laboratory of Immune Regulation, Wakayama Medical University)<br>Koichi Nakanishi (Department of Pediatrics, Wakayama Medical University)<br>Hiroaki Ida (Division of Respirology, Neurology, and Rheumatology, Kurume University School of Medicine)<br>Hiroo Yoshikawa (Division of Neurology, Hyogo College of Medicine)<br>Atae Utsunomiya (Imamura Bun-in Hospital)<br>Tomoaki Taguchi (Department of Pediatric Surgery, Faculty of Medical Sciences, Kyushu University)<br>Takayo Arisato (Minami Kyushu National Hospital)<br>Keizo Sugaya (Department of Neurology, Tokyo Metropolitan Neurological Hospital)<br>Kazumoto Iijima (Department of Pediatrics, Kobe University)<br>Isao Hozumi (Gifu University)<br>Yoshihide Sunada (Department of Neurology, Kawasaki Medical School)<br>Takehiko Hiroma (Nagano Children's Hospital)<br>Kenichi Yamahara (National Cerebral and Cardiovascular Center)<br>Kanji Sugita (Department of Pediatrics, University of Yamanashi)<br>Shinobu Kawakatsu (Department of Psychiatry, Yamagata University)<br>Tomoki Kosho (Clinical and Molecular Genetics, Shinshu University)<br>Keiichi Ozono (Department of Pediatrics, Osaka University)<br>Akira Kinoshita (Atomic Bomb Disease Institute, Nagasaki University)<br>Noriko Fujii (Fukuchiyama City Hospital)<br>Tomoko Komagamine (Dokkyo Medical University)<br>Etsuro Ito ((Department of Pediatrics, Hirosaki University)<br>Kenta Nakai (The Institute of Medical Science, The University of Tokyo)<br>Nobuhito Saito (Department of Neurosurgery, The University of Tokyo)<br>Yuzuru Shibuya (Nihonkai General Hospital)<br>Masanori Takahashi (Department of Neurology, Osaka University)<br>Eri Hirasawa (Research Institute for Diseases of Old Age, Juntendo University)

	Hitoshi Osaka (Department of Pediatrics, Jichi Medical University)<br>Osamu Kano (Department of Neurology, Toho University Omori Medical Center)<br>Koji Abe (Department of Neurology, Okayama University Graduate School of Medicine, Dentistry and Pharmaceutical Sciences)<br>Satoru Nagata (Department of Pediatrics, Tokyo Women’s Medical University)<br>Kayoko Saito (Institute of Medical Genetics, Tokyo Women’s Medical University)<br>Harukazu Suzuki (Cellular Function Conversion Technology Team,RIKEN Center for Life Science Technologies<br>Yutaka Suzuki (Graduate School of Frontier Science, University of Tokyo)<br>Kei Fukami (Division of Nephrology, Department of Medicine, Kurume University School of Medicine)<br>Nobuhiko Okamoto (Department of Medical Genetics, Osaka Medical Center and Research Institute for Maternal and Child Health)<br>Yukihide Iwamoto (Department of Orthopaedic Surgery, Faculty of Medical Sciences, Kyushu University)<br>Katuya Urakami (Department of Biological Regulation, Tottori University Faculty of Medicine, Tottori University)<br>Kenji Kurosawa (Division of Medical Genetics, Kanagawa Children’s Medical Center)<br>Atsushi Masamune (Department of Gastroenterology, Tohoku University)<br>Masaya Nakamura (Department of Orthopaedic Surgery, Keio University School of Medicine, Keio University)<br>Taiichi Matumoto (Department of Orthopaedic Surgery, Kurashiki Central Hospital)<br>Hiroyuki Shimada （Department of Neurology, Osaka City University Graduate School of Medicine, Osaka City University）<br>Masatoshi Takagi (Department of Pediatrics, University Hospital of Medicine, Tokyo Medical And Dental University)<br>Please note that more collaborating institutions and researchers may be added in the future.
Target diseases	Pediatrics: the following intractable pediatric diseases: hematologic malignancies such as Fanconi anemia; immune diseases such as congenital immunodeficiency; endocrine/metabolic diseases such as type I diabetes mellitus; neuropsychiatric disorders such as West syndrome; muscular diseases such as congenital muscular dystrophy and rhabdomyolysis; and cardiovascular diseases such as long QT syndrome; and hereditary diseases such as Li-Fraumeni syndrome<br>Orthopedic surgery: hereditary intractable diseases such as osteogenesis imperfecta, and intractable cryptogenic diseases of which causes are unknown such as ossification of the posterior longitudinal ligament<br>Endocrinology : intractable endocrine/metabolic diseases such as lipodystrophy<br>Neurology: intractable neurodegenerative diseases such as spinal muscular atrophy and Parkinson's disease<br>Gastroenterology and hepatology: intractable gastrointestinal diseases such as inflammatory bowel disease<br>Hepato-pancreato-biliary Surgery and Transplantation: intractable hepato-biliary and pancreatic diseases such as Byler disease<br>Gastrointestinal surgery: intractable gastrointestinal diseases such as

	inflammatory bowel disease<br>Nephrology: intractable renal diseases such as polycystic kidney disease<br>Respiratory medicine: intractable respiratory diseases such as severe juvenile emphysema and idiopathic interstitial pneumonia<br>Cardiovascular medicine: intractable cardiovascular diseases such as Brugada syndrome and long QT syndrome<br>Oral and maxillofacial surgery: intractable oral and maxillofacial diseases such as multiple jaw cysts and delayed tooth eruption<br>Cardiovascular surgery: severe forms of cardiac failure such as dilated cardiomyopathy, and intractable cardiovascular diseases such as valvular heart disease<br>Neurosurgery: intractable neurosurgical diseases such as Moyamoya disease<br>Otolaryngology: intractable otolaryngological diseases such as hereditary inner ear deafness<br>Plastic and reconstructive surgery: intractable diseases in plastic and reconstructive surgery such as facial hemiatrophy of Romberg and spontaneous keloid formation<br>Dermatology: intractable dermatological diseases such as epidermolysis bullosa<br>Urology: diseases resulting in congenital genitourinary disorders such as autosomal dominant polycystic kidney disease (ADPKD); and diseases resulting in genitourinary tumors such as Von Hippel-Lindau syndrome, tuberous sclerosis, Birt-Hogg- Dubé syndrome, Multiple Endocrine Neoplasia and Hereditary pheochromocytoma, paraganglioma syndrome<br>Hematology and oncology: hematologic malignancies such as myelodysplastic syndrome (MDS), and intractable hematologic diseases resulting in hematopoietic disorders such as aplastic anemia, and platelet disorder<br>Psychiatry: intractable neuropsychiatric disorders such as schizophrenia and pervasive developmental disorder<br>Gynecology and Obstetrics: intractable gynecological diseases including gynecological malignant diseases<br>Rheumatology and clinical immunology: intractable connective tissue disease and rheumatic diseases such as systemic lupus erythematosus and scleroderma<br>Diabetes and clinical nutrition: metabolic diseases such as diabetes mellitus<br>Ophthalmology: intractable ophthalmological diseases such as age-related macular degeneration<br>Transfusion medicine and cell therapy: intractable hematopoietic organ diseases such as myelodysplastic syndrome
Names of the genes to be investigated in the study	Undetermined (The whole genome will be analyzed.)
The volume of blood to be withdrawn as a sample	20 cc (Blood will be collected using a standard blood sampling procedure. The risk of serious complications from the sampling procedure is very low.)
Is the tissue obtained during surgery to be used?	■ Yes □ No
Study period	From the date of approval to March 31, 2018 (tentative plan)
If the donor is to be notified of the test	Unpredictable

results, when will approximately this notification take place (how many days after the test)?	
The period for which the test results are retained	The test results will be permanently stored after completion of the study period so that they can be used in studies conducted in the future.
Is the study to participate in the cell banking project?	□ No<br>■ Yes<br>(Name: RIKEN Bioresource Center [RIKEN BRC], Representative: Yuichi Obata, director)<br>Academic significance: To make iPS cells readily accessible to researchers and institutions (including pharmaceutical companies) in and outside Japan so that they can be utilized in a variety of research aimed at elucidating the mechanisms of diseases and developing treatments.<br><br>(Name: National Bioscience Database Center [NBDC] of JST, Representative: Michio Oishi, director)<br>Academic significance: Data registered in the NBDC will be made accessible to researchers working in a range of fields and will help in the development of new technologies, elucidation of the mechanisms of currently incurable diseases, and discovery of new treatments and prophylactic therapies.
Contact information (address and TEL) regarding this study	:
Date of preparation of this written information	October 1, 2015

Purpose of the study:

With regard to the study in which iPS cells are generated from your body tissue sample and used to identify the causes of disease and develop new treatments, please read the separately prepared written information. This written information gives you specific information about the analyses of iPS cell genes.

In order to use the iPS cells generated in the study for treatment in the future, the safety of the iPS cells must be ensured beforehand. This is the most critical point. iPS cells are currently generated by introducing genes using viral vectors. In the future, however, more effective and safer techniques may become available. We will use the most suitable method available at the time. Thus, in order to assess the safety of the generated iPS cells, we must find out where the genes have been inserted. In addition, by comparing the genes of the iPS cells generated from your sample with the genes of the iPS cells from healthy volunteers, we may be able to obtain data that will provide new findings on the disease or lead to the development of new treatments for the disease. For many diseases, the causative genes are completely unknown. Even when some genes are suspected of causing a disease, we often have no clear picture of the onset mechanism and how the abnormality in the genes is causing the disease. In such cases, we may analyze the whole genome (all genes). Thus, it is for these reasons and purposes that we would like to analyze the genes of the iPS cells generated from your sample.

Please note that it will take a number of years to develop a new treatment based on the data obtained in this study. We do not use the human iPS cells generated in this study for treatment; for example, the modified iPS cells will not be directly put back into the patient's body as treatment.

Participation to cell banking project:

As stated in the separate information leaflet, we think it is especially important that the cells collected as well as information and data obtained in this study will be registered in public resource banks such as the those of RIKEN Bioresource Center and National Bioscience Database Center to make them readily available to research institutions (including laboratories inside pharmaceutical companies) in and outside Japan. This will help researchers working in a range of fields to bring together ideas and experiences in iPS cell research and facilitate elucidation of the mechanisms of currently incurable diseases and the development of new treatments.

The procedures for the deposition of your somatic cells and the iPS cells generated from the somatic cells to RIKEN Bioresource Center are as described in the separate leaflet. Data generated in this study including genetic information will also be useful for other medical research. Data obtained from you will be, after anonymization (removal of the information including your name and address that can be used to identify you), registered in publicly funded academic databases so that researchers can access the data. We plan to register data from this study in the database of the National Bioscience Database Center (NBDC) of the Japan Science and Technology Agency (JST). JST is an agency under MEXT and promotes and funds scientific research projects in Japan. NBDC was founded in 2011. Data registered in the NBDC will be made accessible to researchers from various fields and will help in the development of new technologies, elucidation of the mechanisms of currently incurable diseases, and discovery of new treatments and prophylactic therapies.

Study methods:
DNA and RNA will be extracted from the iPS cells generated from your body tissue, which will then be analyzed in detail. You will not be asked to undergo any additional procedure for this study. The information obtained as a result of analyzing your genes in this study may be provided to outside organization for research use. We may also transfer your somatic cells, DNA, RNA or cells derived from your somatic cells to other organization where genetic analysis then might be carried out. In either case, certain conditions must first be met, such that the organization may conduct the research only after the ethics committee approves (unless the organization's Ethics Committee or equivalent decides such approval is not required according to the applicable rules or guidelines).
How the subject may review the study protocol and other study-related documents:
Upon your request, we will be able to give you access to the study protocol in so far that it does not affect the protection of personal information or diminish the originality of the study. In addition, if necessary, we can give you information on genetic analyses and explain to you how genes are studied.

(3) Benefits and risks of participating in this genetic analysis study
You will not benefit directly from participating in this genetic analysis study. The possible risk is that detailed genetic analysis, particularly of DNA, may reveal genetic information not directly related to the disease under study. If such information is disseminated to third parties, it may constitute a serious violation of privacy.

(4) Benefits and risks of not participating in the genetic analysis study
As a rule, the results of genetic analyses will not be disclosed to you because the significance of such data is usually not clear at the time. Thus, your decision to participate in the study or not will have no influence on your medical care.

(5) Your personal information will be kept confidential
Medical doctors have an obligation to protect personal information of patients, as stated in the criminal law. Genetic information particularly has to be protected under the strictest control. Your records related to genetic diagnosis and genetic counseling will be stored in a locker, kept separated from other medical records, and cannot be taken out.
Because the results of genetic analysis may cause various issues, your genetic information will be handled with care. Prior to the genetic analysis, your personal information (e.g., name and address) will be removed from your sample and your medical information, and will be replaced with a code. This procedure is called anonymization. The link table used to link the code with your personal information (linkable anonymization) will be managed by a personal information custodian who will be a doctor working in the hospital where your sample has been collected. This means that the person who analyzes your genes only receives the code, and this prevents the person from learning whose genes are being analyzed. However, when it is thought that the results of genetic analysis could shed light on the cause of your disease, the code may be broken at the hospital where your sample has been collected and used to access only your medical records.

(6) Will the participant be notified of the results of genetic analysis?
As a rule, participants will not be notified of the results of genetic analysis because the results will include a lot of data that have no clearly determined meaning.

20151001 (Supplement 8)

Informed Consent Form

[Name of the person to whom the consent is given if the informed consent discussion is held in Kyoto University] or [Name of head of the medical institution, etc. if the informed consent discussion is held outside Kyoto University]

Study title: Genetic Analysis Study Using Human Disease-Specific iPS Cells

I have been given information about this study, in the course of which the iPS cells generated from a sample of my body tissue will be used for genetic analyses. The following items regarding the study have been explained to me by the study doctor using the separately prepared written information. I volunteer to take part in this study.

<What is a gene?>
<Genes and disease>
<Participation in the genetic diagnostic study>
<Information to help you decide whether or not to participate in this genetic diagnostic study>

- You are free to choose to participate or not to participate in the genetic diagnostic study. If you change your mind later, you may withdraw your consent at any time.
- The plan of the genetic diagnostic study
  - Participation to cell banking project
  - The purpose of the study
  - The study methods
  - How the subject may review the study protocol and other study-related documents
- Benefits and risks of participating in this genetic analysis study
- Benefits and risks of not participating in the genetic analysis study
- Your personal information will be kept confidential.
- Will the participant be notified of the results of genetic analysis?
- Publication of analysis data
- Intellectual property rights generated from this study
- Handling and disposal of samples after completion of the genetic analysis study
- Who will pay the cost of genetic analysis?
- Genetic counseling for any concerns you may have related to heredity/genes, and any anxiety you may feel before and after genetic analysis
- Contact information for more information and complaints

Patient (Name)

Date of consent: MM/DD/YYYY

Study participant (Signature)

Representative of the participant (Signature)

(Relationship of the legal representative to the participant)

I confirm that I have given the participant detailed information about the study and that the donor has consented to participate voluntarily in the study.

Institution (Name)/Department (Name)

Doctor who has conducted the informed consent discussion (Name)

The hospital will keep the original of the signed form, and a copy will be given to the donor.

（京大医遺伝子解析様式２－２）
Kyoto University Genetic Analysis Form 2-2

Consent Withdrawal Notification

Dean of the Graduate School of Medicine, Kyoto University

Director of the Kyoto University Hospital

Director of the Center for iPS Cell Research and Application, Kyoto University

I, the undersigned, hereby withdraw the consent I granted at an earlier date by signing the Informed Consent Form regarding the participation in the following studies in which the iPS cells generated using my somatic cells or tissues are used.

I ask that the specimen I have donated, the iPS cells or other materials derived from the specimen, and medical information associated with the donated specimen be destroyed and no longer be used.

Study Titles:

"The Generation of Human Disease-Specific iPS Cells and the Use of Such iPS Cells for Disease Analysis"

"Genetic Analysis Study Using Human Disease-Specific iPS Cells"

Print Name of Person Withdrawing Consent<br>____________________________________<br>(Signature)	Date<br>____________________
Print Name of Legal representative<br>____________________________________<br>(Signature)<br>(Relationship of the legal representative to above person)	Date<br>____________________

Receipt of consent withdrawal notification

I have received the notification withdrawing consent to the participation in the studies as above.

Print Name of Person at hospital<br><br>Name of hospital<br>Department	Date of receipt<br><br>____________________________
Memo：	

（京大医遺伝子解析様式２－２）
Kyoto University Genetic Analysis Form 2-2
