# Supplementary material for: In vitro methods to ensure absence of residual undifferentiated human induced pluripotent stem cells intermingled in induced nephron progenitor cells
Source: PLoS One. 2022 Nov 15;17(11):e0275600. doi: 10.1371/journal.pone.0275600 (PMC9665373; doi:10.1371/journal.pone.0275600)
Supplement: S1 File — (ZIP) [file pone.0275600.s021.zip › S1_documents/πÇÉBlood_DonorπÇæC687G540_ICF_v12e.pdf]

iPS Cells for the Future

Research on an HLA-Homozygous Donor-Derived iPS Cell Stock from Apheresis Donors for Regenerative Medicine

<u>Information Sheet</u>

(Ver. 12, Created on 21 January 2020)

Center for iPS Cell Research and Application Foundation

(CiRA Foundation)

Kyoto University Hospital

Center for iPS Cell Research and Application (CiRA), Kyoto University

—Request for Cooperation for Research—

The aim of this research is to generate iPS cells from donors with specific immunological types, distribute them to research and medical institutions in Japan and overseas, and eventually differentiate the generated iPS cells into target cells to treat patients by transplanting the target cells.
If you decide to cooperate with this research after reading this information sheet and listening to the explanation from the research-participating physician or research coordinators, please sign the enclosed consent form.

Table of Contents

What Is The Object of This Research? 3
An Ethics Committee Has Reviewed This Research 5
Who Can Participate in This Research? 5
Research Duration 6
iPS Cell Stock for Regenerative Medicine 7
- What Is An iPS Cell Stock? 7
- Where Will The iPS Cell Stock Be Distributed? 7
- How Will The iPS Cell Stock Be Used in Research? 7
- How Will The iPS Cell Stock Be Used in Treatment? 8

What Will Happen If You Take Part? 8
Gene Analysis 13
What Are The Possible Benefits and Disadvantages of Taking Part? 14
What Are The Possible Risks and Burdens Associated with Donation? 14
- Risk from tissue (blood) collection 14
- Risk of personal information leakage 15
- Other burdens 15

Voluntary Consent and Time Limit for Withdrawal 16
Handling of Test Results 16
Sample Handling Policy 17
Announcement of Progress and Results of This Research 18
Handling of Various Rights including Intellectual Property Rights 19
Who Is Organizing and Funding This Research, and Is There Conflict of Interest? 19
Who Should You Notice If Your Contact Information Has Changed? 21
Contact Information 21

What Is The Object of This Research?

The Center for iPS Cell Research and Application (CiRA), Kyoto University, where Professor Shinya Yamanaka, who developed the first method for generating iPS cells (induced pluripotent stem cells), works as the director, is the world's first core institute dedicated to iPS cell research. The CiRA Foundation, a general incorporated foundation (changed to a public interest incorporated foundation in April 2020), is an organization established to carry out the iPS Cell Stock Project for Regenerative Medicine, a Japanese national project that was the responsibility of CiRA until the end of fiscal year 2019.

We are continuing this research in cooperation with researchers in Japan and overseas to help patients with intractable diseases for which no current treatment exists.

iPS cells have the special ability to differentiate into any type of cell in the body. Therefore, if we can generate iPS cells from a part of our own body and store them, treatment without immune rejection is expected. However, complicated, lengthy procedures are required to ensure the safety and quality of iPS cells. Therefore, in cases of urgent care, it is impossible to generate iPS cells from the patient's own cells, differentiate them into target cells, and use them to treat the patient promptly.

As an alternative strategy, we are generating iPS cells from somatic cells of third-party donors in advance. Donors will be selected so that cellular products made from these iPS cells will have a low risk of immune rejection. Storing these iPS cells and their products in advance makes it possible to reduce the time for treatment. In these cell therapies, compatibility between the donor and recipient is important, because it reduces the chance of immune rejection. Compatibility is based on HLA (human leukocyte antigens). Our research has shown that storing iPS cells of 50 different combinations of HLA types is sufficient to cover 70% of the Japanese population.

The aim of this research is to generate iPS cells from HLA-homozygous donors, that is, individuals who have specific HLA types suitable for cell transplantation (in other words, which are less likely to cause rejection), and evaluate the safety, differentiation potential (ability to produce different cell types), and gene function of the iPS cells, as well as distributed to research and medical institutions in Japan and overseas and use them in cell therapies for a large number of patients in the future.

◆What are HLA?

Just as red blood cells have ABO types, cells other than red blood cells have HLA types. HLA are proteins that exist in all cells of the body and are involved in immunity. They act as markers that distinguish your own cells from foreign cells. Therefore, the transplantation of cells or organs between persons with different HLA types results in an immune response that can damage the transplanted cells or organs and harm the patient. HLA consist of many genes such as HLA-A, HLA-B, HLA-C, HLA-DR, HLA-DQ, and HLA-DP (haplotypes). Each gene has several dozen polymorphisms, and the overall HLA type is determined by their combination. These combinations vary from person to person, resulting in tens of thousands of HLA combinations.

An example HLA type could be written as A24-B52-C12-DR15-DQ06-DP09, where each polymorphism is assigned a number according to its gene.

HLA typing reveals two HLA for each haplotype. One of these two is from the father and the other from the mother. Chromosome-carrying genes inherited from the parents are paired. Likewise, the HLA type inherited from each parent is paired.

The degree of matching HLA types is very important for the transplantation of organs or tissues between non-related individuals. Therefore, the HLA type of iPS cells used for transplantation medicine needs to be similar to that of patients.

◆About genes

Genes are the blueprint for the human body.

The human body is made up of trillions of cells. Each cell contains all the genes needed to form the whole body, but only certain genes are essential for a given cell. Often the gene activity changes. Gene activity changes in everyone and rarely affects our health, but sometimes they can cause disease.

Everyone has such gene changes. Some gene changes do not interfere with our daily activities, including only the difference in the face, body shape, and constitution, whereas others are related to some diseases. There are various disease-related gene changes, ranging from no symptoms to serious symptoms.

A cell has tens of thousands of genes scattered in it. All genetic information is collectively called “genome.” The human body has approximately 60 trillion cells, with each cell containing all genes.

An Ethics Committee Has Reviewed This Research

This research protocol has been reviewed by the Kyoto University Graduate School and Faculty of Medicine, Ethics Committee, and approved by the Dean of the Graduate School of Medicine, Kyoto University, the Director of Kyoto University Hospital, the Director of CiRA, and the Representative Director of the CiRA Foundation. Another committee composed of multiple members including external members reviews whether this research is being conducted scientifically and ethically.

Who Can Participate in This Research?

Apheresis donors who underwent HLA testing with the Japanese Red Cross Society and were determined to have specific HLA combinations. <u>To participate in this research, you must meet all of the inclusion criteria and none of the exclusion criteria.</u> We will ask you to undergo blood tests and a medical interview as described in the section "What Will Happen If You Take Part?" to make sure that you do not meet the exclusion criteria.

Inclusion criteria (requirements which allow you to participate):
1) Those who are at least 20 years old when informed consent is obtained
2) Those who can voluntarily sign an informed consent document

Exclusion criteria (those who meet any of the following criteria cannot participate):
1) Those who test positive for at least one of the following infectious diseases
- Hepatitis B
- Hepatitis C
- Human immunodeficiency virus infection
- Adult human T-cell leukemia
- Parvovirus B19 infection
- Syphilis
- Cytomegalovirus infection

2) Those who have the following diseases or history of diseases
- Syphilis, West Nile fever, Chlamydia infection, gonorrhea, tuberculosis, malaria, babesiosis, Chagas' disease, leishmaniasis, African trypanosomiasis, and other bacterial infections
- Malignant tumors
- Confirmed or suspected transmissible spongiform encephalopathy or other dementia, stroke, or epilepsy
- Conditions which are judged to be serious inherited diseases that would pose a problem for the generation and use of the iPS cell stock

3) Women who are pregnant/breastfeeding or possibly pregnant

* Test items other than the infection tests listed above may be added as needed. In such situations, we may contact you again.
* You may not be able to participate in this research according to the physician’s judgement for a reason other than listed above.

Research Duration

From the date of approval by the Ethics Committee to 31 March 2023.

iPS Cell Stock for Regenerative Medicine

● What Is An iPS Cell Stock?

In this research, we will establish a system in which iPS cells made from donor cells will be supplied promptly when required for the treatment of disease by preparing, expanding and storing iPS cells with minimum personal information. This collection of iPS cells is called an iPS cell stock.

● Where Will The iPS Cell Stock Be Distributed?

Much research is needed to develop new treatments using iPS cells. Especially, it is important to cooperate with enterprises that can provide both stringent quality management and large-scale production in order to widely disseminate treatment methods using iPS cells to the public. For this reason, in this research, we will distribute iPS cells stored for medical use and data such as gene analysis information to researchers, research institutions (including profit-making enterprises such as pharmaceutical companies), medical institutions, and so on in Japan and overseas. Other research protocols will be applied separately by the institutions which receive the iPS cells. Information on the institutions which receive the iPS cells and individual research protocols will be widely released through the CiRA Foundation website, letters, and other means.

● How Will The iPS Cell Stock Be Used in Research?

When iPS cells are distributed for research purposes to research institutions in Japan, we will give permission for use only for research that is conducted in accordance with Japanese regulations and whose scientific and ethical validity is approved by ethics committees established by Kyoto University and collaborative research partners in accordance with related domestic ethical guidelines (e.g., “Ethical guidelines for medical and health research involving human subjects” and “Ethical guidelines for human genome/gene analysis research”). When supplied to research institutions outside Japan, the iPS cells can be used only for research whose scientific and ethical validity is approved according to the country's regulations.

● How Will The iPS Cell Stock Be Used in Treatment?

The ultimate goal is to establish an iPS cell stock that covers many Japanese people. iPS cells can be induced (i.e., differentiated) to any type of cells in the body. Only after iPS cells are differentiated can they be used for clinical treatment.

Using the iPS cell stock, the research and medical institutions will produce differentiated cells of high quality to be used for transplantation therapy. After the differentiated cells are transplanted into a small group of patients to confirm the safety and effectiveness of the therapy, these cells will be used to treat patients at Kyoto University Hospital and other medical institutions. For the transplantation therapy, the research or medical institution will submit a research protocol according to the procedure specified in government guidelines.

If research demonstrates the effectiveness of the transplantation therapy using these differentiated cells, the differentiated cells may be sold as a drug product by the researchers or research institutions (including pharmaceutical companies and other for-profit companies) in and outside Japan. In the case that iPS cells derived from your cells are used to manufacture commercialized products, you will not be informed and you will not financially benefit from these products.

What Will Happen If You Take Part?

The first step is that we will explain to you about the research using this information sheet. If you consent to participate in the research, your blood will be drawn to produce iPS cells. The flowchart of the research participation is as follows.

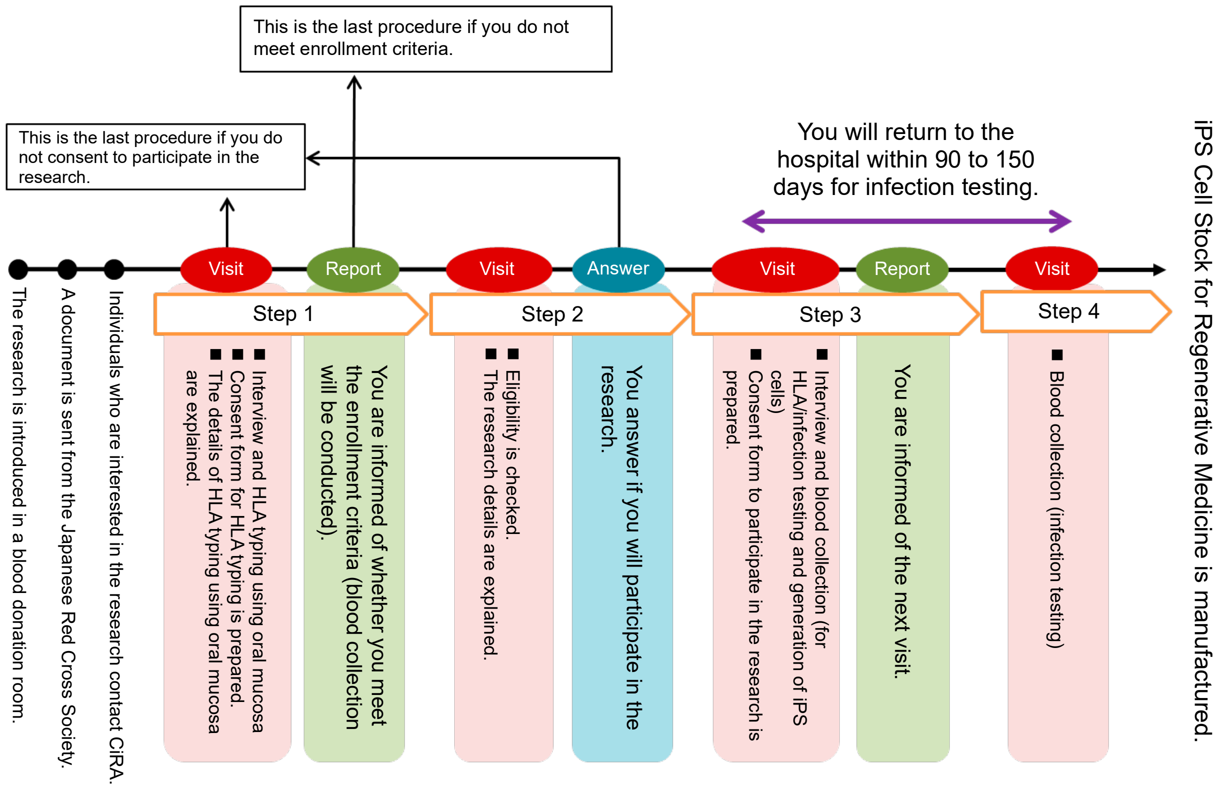

Note 1: A guide document is sent from the Japanese Red Cross Society only to individuals who gave consent to receive the document in a blood donation room.

Note 2: For step 1, the research coordinator may explain the details and obtain consent.

[Step 1] Explanation of HLA typing, informed consent, interview and HLA typing of oral mucosa

You will visit the iPS Cell Outpatients Department, Division for iPS Cell Application Development, Kyoto University Hospital, and receive an explanation about HLA typing your oral mucosa. If you wish, you can visit a Japanese Red Cross Society blood donation room or a facility near where you live to receive the explanation.

After we confirm your willingness to have HLA typing of the oral mucosa, you will sign the "Informed consent form for HLA typing of the oral mucosa." You will then complete the interview sheet to confirm that you do not have any health problems and provide cells from the mouth for the HLA typing.

To take cells from the mouth, you will be asked to rub the inside of the cheek several times using a stick with a sponge. On the basis of the results of the HLA typing, your research coordinator will inform you whether you will go to the next step at a later date. It takes approximately 1 hour to sign the informed consent form, conduct the interview, and conduct

the HLA typing.

If considered unnecessary by the researcher or physician, HLA typing of the oral mucosa will not be done, and you will go to Step 2.

[Step 2] Explanation about the research

You will visit the iPS Cell Outpatients Department, a Japanese Red Cross Society blood donation room, or a facility near where you live and receive an explanation of research using this information sheet.

You will also be asked for your medical history and the presence of any infections and to consider whether you will take part in this research. The explanation will take approximately 1 hour.

To carefully think about whether you will participate in the research, you do not have to reply on the day of the explanation. You will be provided a postcard for the reply, which we ask you return within 1 week to the iPS Cell Outpatients Department to let us know whether you consent to participate in the research or not. If you do not agree to participate, then you will not proceed to later steps. If you consent to participate in the research, the research-participating physician or research coordinator will contact you again to arrange the schedule for the next visit to the iPS Cell Outpatients Department to go to Step 3. Expenses for traveling to the iPS Cell Outpatients Department will be paid according to the policies of the CiRA Foundation.

If you voluntarily consent on the day of the explanation, you can go to Step 3 on the same day. In this case, please tell the research-participating physician or research coordinator.

[Step 3] Signature of the informed consent form, interview, and blood collection

Blood will be drawn only at the iPS Cell Outpatients Department, Kyoto University. After you visit the iPS Cell Outpatients Department and your willingness is confirmed again, you will sign the informed consent form and provide approximately 100 mL of blood.

Your blood will be used for iPS cell generation, HLA typing, infection tests, and blood typing, and some of the blood will be frozen and stored for a long time. Because this research aims

to transplant cells derived from your iPS cells into patients, some of the blood will be used to test whether you have the following infections. (In addition to these infection tests, other test parameters may be added as necessary, which may increase the volume of blood drawn.) It will take approximately 1 hour to sign the informed consent form, respond to the interview, and draw your blood.

[Details of infection tests]
Hepatitis B (HBV), Hepatitis C (HCV), Human immunodeficiency virus (HIV) infection, Human T-lymphotropic Virus (HTLV), parvovirus B19 infection, Cytomegalovirus infection, *Treponema pallidum*, etc. (based on the “Guidelines on Ensuring the Quality and Safety of Pharmaceuticals and Medical Devices Derived from Processing of Allogeneic Human Induced Pluripotent Stem (-Like) Cells” issued by the Ministry of Health, Labour and Welfare)

On the basis of the results of the HLA typing and infection tests, we will send the “Notice on your next visit” by mail several weeks later to inform you whether you will go to Step 4. In most cases, you will not be informed of the results of the HLA typing and infection tests. However, if your health can clearly benefit from the infection tests, you may be informed.

Some of the blood drawn in Step 3 will be stored to generate iPS cells. Using the stored blood can minimize your burden. If iPS cells cannot be produced from the stored blood, however, we may ask you to give your blood again. In this case, approximately 100 mL of blood will be drawn in the same manner.

[Step 4] Blood collection (the second infection test)

After approximately 3 months following the infection tests in Step 3, you will visit the iPS Cell Outpatients Department, a Japanese Red Cross Society blood donation room, or a facility near where you live to receive the same infection tests as in Step 3. The reason is that the future transplantation of cells derived from your iPS cells into patients requires strict infection testing. The infection test requires drawing approximately 10–30 mL of blood and will take approximately 30 minutes. As with Step 3, you will not be informed of the results of the infection tests, but if your health can clearly benefit, you may be informed.

Reason for conducting blood collection to generate iPS cells before obtaining the results of the second infection test

There is a time period during which infection cannot be detected in a test even if you are infected with a virus. Therefore, even if the results of the first infection tests are negative, infection may be hidden with no symptoms. In order to confirm that you do not have any infection at the time of the first test, the second infection test is needed (approximately 3 months after the first test). For this reason, the blood used to generate iPS cells will be collected during the same period as the first infection tests, but only after confirming no infection in the second infection tests will the collected blood be used to generate iPS cells.

Please join this research only after you understand that the following situations may happen depending on the progress of this research:

- Currently, we can produce an iPS cell stock from only 3 persons per year because of technical limitations. After the end of Step 1, you may wait more than several months until the start of Step 2.

- If you join Step 1 but then the HLA types necessary for the research are fully collected, your participation may be stopped without going to Step 2 or Step 3. Similarly, you sometimes cannot go to Step 4 even after you have participated in Step 3. In such cases, a research-participating physician or research coordinator will inform you of discontinuation and will immediately discard all your drawn blood and information such as test results no later than the time when the research period ends.

[Handling of collected cells]

The blood drawn from you will be sent to the CiRA Foundation, which will extract cells from the blood to produce iPS cells. The iPS cells will be tested for genetic information in detail to check whether they are suitable for research and medical care, stored in a freezer, and used for various research and treatments in the future.

If your blood does not meet the criteria for producing iPS cells, all your samples will be discarded by the time the research period ends. Even if we decide to produce iPS cells,

technical reasons may prevent the production. In such cases, we will not inform you that iPS cells have not been produced.

[Occasions in which we will contact you again]
Please be aware that we may contact you again for the following reasons through the address that you registered.

- When we ask for blood collection again because iPS cells cannot be produced or for other reasons
- When important changes are made to the content of this research
- If this research is progressing as planned, the iPS Cell Stock for Regenerative Medicine will also be produced using skin cells in addition to blood cells

  (In this case, we will file a separate research protocol as needed and may ask you to donate skin or other tissues again)
- When your information is needed for management of the iPS Cell Stock for Regenerative Medicine

  (We may ask you about your health status after the blood collection)

We greatly appreciate your cooperation despite the many burdens.

Gene Analysis

In this research, we will analyze the genes in your donated blood cells and the produced iPS cells. To use iPS cells for medical treatment, it is most important to check the safety of the iPS cells. This check includes evaluating the function of selected genes in the cells. For this reason, we compare the genomes and genes of blood cells and iPS cells.

We also have to check whether necessary genes work in the iPS cells and cells differentiated from the iPS cells to confirm that the cells function normally. Finally, we need to check if any genes that may cause cancer or other diseases are active in the cells.

To achieve these objectives, all genes may be analyzed in your blood cells, the produced iPS cells, and cells differentiated from the iPS cells after you consent to participate in this research. The gene analysis information obtained will be supplied together with the iPS cell stock to researchers, research institutions (including pharmaceutical and other for-profit companies), and medical institutions in Japan and other countries. When DNA is analyzed in detail, your genetic information will be obtained. When this information is provided to an outside research institution, we will pay great attention to strictly control personal information to prevent your privacy from being violated, as described in the subsection “Risk of personal information leakage”. Because this analysis is not for medical care and the information from the gene analysis is not directly meaningful for your health in many cases, we will not inform you of the results.

All genes in cells differentiated from the iPS cell stock may be analyzed at the research institutions provided with the stock. In this case, the research institutions will submit a research protocol according to the procedure specified in the government guidelines and then carry out the research after approval.

What Are The Possible Benefits and Disadvantages of Taking Part?

Participation in this research will not directly benefit you as an individual. However, any successful results yielded in this research will lead to the development of new medical treatments. In other words, your participation will provide benefit to future generations. Disadvantages caused by participation are described in the section “What Are The Possible Risks and Burdens Associated with Donation?”

What Are The Possible Risks and Burdens Associated with Donation?

● Risk from tissue (blood) collection

As in the case of routine testing, blood collection is very unlikely to cause serious health damage. However, if you experience any health problem, you can receive treatment at

Kyoto University Hospital or other medical institutions. The expenses for treatment will be paid by the CiRA Foundation, with no financial burden on you. This research is covered by compensation insurance for clinical research. If serious disability caused by the blood collection in this research occurs, you will be compensated by insurance. For details about the insurance coverage, please see the Appendix.

● Risk of personal information leakage

Blood cells provided for iPS cell generation and the information obtained in the interview will be given a new code after removing personal information that identifies you, such as name and address, so that no one can identify you. The correspondence table for linking tissue (blood) donors to this code will be managed by a third party who is not involved in this research (Personal Information Manager). This prevents anyone other than the Personal Information Manager from knowing which donor the iPS cells are derived from.

The correspondence table will be strictly stored in a lockable cabinet at the CiRA Foundation and cannot be accessed by anybody except the Personal Information Manager. The information that can identify individuals will not be seen by researchers other than the Personal Information Manager and not provided to external institutions. These steps will be taken to minimize the risk of leaking personal information.

● Other burdens

Persons who participate in this research will be asked to visit a Japanese Red Cross Society blood donation room, Kyoto University Hospital, or other medical institutions 4 times in total (this number may change). It will take approximately 1 hour per visit to have the explanation and blood collection. When additional blood is needed to produce iPS cells, additional infection testing is needed, or when a change occurs in the research, we may contact you again to ask you to visit Kyoto University Hospital, a Japanese Red Cross Society blood donation room, or a facility near where you live.

Participants will not be paid for participating in this research. When you visit the iPS Cell Outpatients Department or a blood donation room after signing the informed consent form, travel expenses will be paid to you according to the policies of the CiRA Foundation.

Voluntary Consent and Time Limit for Withdrawal

Your participation in this research is entirely voluntary. Even after giving consent, you are free to withdraw it by providing written notice any time before iPS cells are produced from your blood without disadvantage to yourself. Thereafter, all cells including the iPS cells and the information will be discarded.

However, after the iPS cells have been produced, there are circumstances where you cannot withdraw your consent such as when the iPS cells are planned for a cell therapy to treat a patient, a pharmaceutical company has decided to release a drug product based on your iPS cells to the market, or other conditions that would greatly impact patient health.

<How to withdraw consent>
Please call the research coordinator via the contact information described later in this information sheet.

If you cannot call this person, please send the consent withdrawal form by mail to

Division for iPS Cell Application Development, Kyoto University Hospital
54 Kawahara-cho, Shogoin, Sakyo-ku, Kyoto 606-8507, Japan

Handling of Test Results

As explained in the section “What Will Happen If You Take Part?”, your blood will be tested in detail for HLA type and infections and you will be interviewed to confirm that the iPS cells produced from your samples meet the purpose of this research. We will not usually inform you of the results of the infection tests, but if your health can clearly benefit from the results, we may contact you again.

Your blood cells and produced iPS cells will be tested in detail, including HLA typing, karyotyping, and gene analysis. Because these tests are not for medical care and the test results are not directly meaningful for your health in many cases, you will not usually be informed of the results. However, if your health can clearly benefit from the results, we may inform you of the results after consultation with experts (e.g., Clinical Genetic Unit, Kyoto

University Hospital).

As described in the section “Gene Analysis,” although gene analysis may be done on cells differentiated from the iPS cell stock at the research institution provided with the stock, you will not be informed of the results for the same aforementioned reason.

Sample Handling Policy

The CiRA Foundation will carefully manage your provided tissue (blood) in this research, iPS cells derived from your blood, genetic information, information from the medical interview, results from the infection testing and HLA typing, DNA, and data from research using these samples (research data). The ownership of these items belongs to the CiRA Foundation. Some of the samples may be stored at external institutions, but under the responsibility of the CiRA Foundation.

The iPS cell stock and other cells, interview information necessary for handling cells, and information on research data will be stored for a long time after the end of the research period to use them for future transplantation therapy and research. If considered necessary to achieve these purposes, your iPS cells and the information may be used persistently beyond the research period.

The iPS cells produced in this research may be stocked, processed and then transplanted into patients who suffer from incurable disease or injury. External research institutions may request the provision of your iPS cells for research purposes. To carry out such research and transplantation therapy, the research and medical institutions will submit a research protocol according to the procedure specified in government guidelines. A company may also sell cells derived from your iPS cells as a drug product. To do so, if requested by the company, the ownership of the iPS cells necessary for the drug formulation may be transferred from the CiRA Foundation to the company. In any case, we will not obtain informed consent from you for each transplantation therapy. In this research, germ cells will not be produced.

As explained in “[Step 4] Blood collection (the second infection test)” in “What Will Happen If You Take Part?”, research using your samples may be stopped in the middle of the research period. In such cases, all of your blood and test results will be discarded by the time the research period ends if not immediately.

Even if the research must be stopped or interrupted early, if the iPS cells have already been produced from your blood, the iPS cells and the information on the test results will be continuously stored. The reason is that early disposal of the iPS cells produced from your blood may bring about a great loss in the development of new therapies at external institutions to which the iPS cells have been supplied and are carrying out related research. If no iPS cells are produced from your blood, then all of your blood and test results will be disposed of.

<Information to be provided about you and your cells>
Blood, background information (gender, age, and health status), HLA type, karyotype, infection test results, gene analysis information, etc.

Announcement of Progress and Results of This Research

We will regularly announce progress of this research through the CiRA Foundation website, letters, and other methods. When other medical institutions perform cell therapies using iPS cells from this research, the results may be announced through their public communications.

The results of this research may be presented at scientific meetings, published in academic journals, and made public on databases. However, in such situations, your personal information (e.g., name) will never be released.

<Possible events to happen>
Although not planned at the moment, new research may be carried out at research institutions (including company laboratories) in and outside Japan in the future, depending

on the progress of this research. A new biobank or database may also be constructed.

For these reasons, iPS cells produced from your cells, their differentiated cells, and your information may be stored in these biobanks or databases and required continuously.

In such cases, any new research will be reviewed by a qualified review committee to confirm that it is important research, worth your participation and does not pose great risks or burdens to you before your cells and information are continuously used. The review results will be released by the institution receiving your cells and information.

In some cases, details on the use of cells and information provided to the company cannot be released to keep the research development status confidential.

Handling of Various Rights including Intellectual Property Rights

As a result of research using iPS cells derived from your tissue (blood) and gene analysis, intellectual properties such as patents may be created, and intellectual property rights may be produced. These rights will be managed by the CiRA Foundation. Please understand that these intellectual property rights are granted for value created by the researchers' efforts and their results and not for the donated tissue (blood) itself or the information of the genes contained in the tissue. Furthermore, if financial profit is produced by the intellectual property rights and/or by distributing iPS cells as human cell-based medicinal products from enterprises including pharmaceutical companies, you cannot assert ownership of these rights for the same reason.

Who Is Organizing and Funding This Research, and Is There Conflict of Interest?

1) Director of the entire research project
Shinya Yamanaka (Representative Director of CiRA Foundation, Director/Professor of CiRA)

2) Principal Investigator

Shuichi Matsuda (Director/Professor of the Division for iPS Cell Application Development, Kyoto University Hospital)

3) Research Collaboration Organization

Japanese Red Cross Kinki Block Blood Center

4) Personal Information Manager

Until 31 March 2020

Isao Asaka (Professor, CiRA)

From 1 April 2020

Ryuya Konishi (Present, Head of Planning and Coordination Office, CiRA; from April 2020, Head of General Affairs Office, CiRA Foundation)

5) Physician/researcher who obtained the informed consent and collected blood or other tissues

Name: ____________________ (Affiliation: ______________________)

6) Research Coordinator

Name: ____________________ (Affiliation: ______________________)

This research is not funded by companies and has no conflict of interest. A conflict of interest refers to a situation where funding from others could cause a conflict between the responsibilities of the researchers (or research institutions) and their personal benefit, affecting interpretation of the research results.

Whether this research has conflicts of interest is properly reviewed and managed by the “Kyoto University Conflict of Interest Review Committee for Clinical Research” according to the “Kyoto University Conflict of Interest Policy” and “Kyoto University Conflict of Interest Management Regulations.” All expenses necessary for the research, including costs for infection and blood testing, will be paid using public research grants from the Ministry of Education, Culture, Sports, Science and Technology, the Ministry of Health, Labour and Welfare, and the Cabinet Office.

If this research later uses funds other than public grants, then we will receive the funds according to appropriate procedures and disclose full information on the CiRA Foundation website.

Who Should You Notice If Your Contact Information Has Changed?

We may contact you again after your participation is complete should the contents of this research have changed. Therefore, when your contact information has changed (e.g., move to a new address), please contact the undersigned below.

Contact Information

If you have any questions or concerns about your participation in this research, please feel free to consult a research-participating physician or research coordinator at any time. However, we cannot answer any questions about transplantation therapies such as whether iPS cells derived from you have been generated and stocked and whether cells derived from your iPS cells have been used for a transplantation therapy in clinical practice. If you want to know details of the research protocol, we can disclose them except those under secrecy obligations such as patents.

Contact information:

Research coordinator

Name:______________________

Fill in the name of the person who gave supplemental explanation

Tel:________________________ (10:00 to 17:00 on weekdays)

Informed Consent Form

To: The Director of the Center for iPS Cell Research and Application Foundation (CiRA Foundation),
the Director of Kyoto University Hospital, and
the Director of the Center for iPS Cell Research and Application (CiRA), Kyoto University

I have received an oral and written explanation of the research ("Research on an HLA-Homozygous Donor-Derived iPS Cell Stock from Apheresis Donors for Regenerative Medicine") from the research-participating physician and have fully understood the items of explanation described below. I hereby voluntarily agree to participate in this research. As proof of agreement, I have signed and received the information sheet and a copy of the informed consent form.

- □ Objective of this research
- □ Ethics committee review of this research
- □ Eligibility to participate in this research
- □ Research duration
- □ What cells and clinical information will be provided by me
- □ The iPS Cell Stock for Regenerative Medicine
- □ Research procedures
- □ What will happen to me by participating
- □ Gene analysis
- □ Possible benefits and disadvantages of taking part
- □ Possible risks and burdens associated with the donation
- □ Voluntary consent and time limit for withdrawal
- □ Handling of test results
- □ Sample handling policy
- □ Announcement of progress and results of this research
- □ Handling of various rights including intellectual property rights
- □ Organizers and funders of this research
- □ Who to contact if my contact information has changed
- □ Contact information

[Donor]

Date of consent: ______ Year ______ Month ______ Day ___

Name of donor: ____________________ (Signature) ____________________

[Physician who provided the explanation]

I have explained this research to the donor who signed above and confirmed his/her willingness.

Date of explanation: ____________________

Signature of the physician: ____________________

(If the research coordinator has provided supplemental explanation)

Date of explanation: ______ Year ______ Month ______ Day ___

Name of the research coordinator: ____________________ (Signature)

*The original informed consent form will be stored at the research institution, and a copy will be provided to the subject.

Request for Withdrawal of Consent

To: The Director of the Center for iPS Cell Research and Application Foundation (CiRA Foundation), the Director of Kyoto University Hospital, and the Director of the Center for iPS Cell Research and Application (CiRA), Kyoto University

I hereby withdraw my consent to participate in the “Research on an HLA-Homozygous Donor-Derived iPS Cell Stock from Apheresis Donors for Regenerative Medicine.” I request that all of my blood samples, cells derived from the blood samples, and all associated information be discarded and not be used in the future.

[Donor]
Date of consent withdrawal: ____________ Year ____________ Month ____________ Day ____________
Name of donor: ______________________________
(Signature): ______________________________
Address: ______________________________
Phone number: ______________________________

Acknowledgement of Receipt of Request for Withdrawal of Consent

Your request for withdrawal of consent to participate in the “Research on an HLA-Homozygous Donor-Derived iPS Cell Stock from Apheresis Donors for Regenerative Medicine” has been received.

Date of receipt: ____________ Year ____________ Month ____________ Day ____________

Name of person who received the request: ______________________ (Signature)

Compensation for "Research on an HLA-Homozygous Donor-Derived iPS Cell Stock from Apheresis Donors for Regenerative Medicine"

If an adverse health event* happens to the donor in relation to "Research on an HLA-Homozygous Donor-Derived iPS Cell Stock from Apheresis Donors for Regenerative Medicine," compensation measures will be taken as follows:
*An adverse health event refers to a permanent disability or death caused by injury or disease.

1. Period to be covered

On the basis of the "Flowchart of the research participation" in the information sheet, the following periods will be covered by compensation:

- From Step 3: Signature on the informed consent form to Step 4: Blood collection

  and
- For 6 months after the blood collection in Step 4

2. Compensation to be paid

	Compensation (yen)
Death	40,000,000 (breadwinner)<br>18,000,000 (non-breadwinner)
Permanent disability grade 1	22,000,000
Permanent disability grade 2	20,000,000
Permanent disability grade 3	18,000,000
Permanent disability grade 4	15,000,000
Permanent disability grade 5	13,000,000
Permanent disability grade 6	11,000,000
Permanent disability grade 7	9,000,000
Permanent disability grade 8	8,000,000
Permanent disability grade 9	6,000,000
Permanent disability grade 10	5,000,000
Permanent disability grade 11	3,500,000
Permanent disability grade 12	2,500,000
Permanent disability grade 13	1,500,000
Permanent disability grade 14	1,000,000

*The permanent disability grades are based on Appended Table 1: Table of Disability Grades in the Ordinance for Enforcement of the Industrial Accident Compensation Insurance Act.

3. Main reasons why compensation cannot be paid

(1) Causal relationship with the research can be ruled out

(2) Because of deliberate misconduct or gross negligence of the subject or their lawful heir

(3) Because of abnormal pregnancy (including abortion, premature delivery, and stillbirth, but excluding disorders of the mother), ovular or fetal abnormalities, damage, or disorder, or congenital abnormalities or disorder of the neonate

<Table of Disability Grades>

Grade	Physical Disability	
Grade 1	1	Those who are blind in both eyes
	2	Those who have lost the functions of mastication and speech
	3	Those who are left with serious impairment in functions of the nervous system or psyche and require continuous nursing care
	4	Those who are left with serious impairment in functions of the thorax and abdominal organs and require continuous nursing care
	5	Deleted
	6	Those who have lost both upper limbs above the elbow joint
	7	Those who have completely lost function of both upper limbs
	8	Those who have lost both lower limbs above the knee joint
	9	Those who have completely lost function of both lower limbs
Grade 2	1	Those who are blind in one eye and whose vision has deteriorated to 0.02 or less in the other eye
	2	Those whose vision in both eyes has deteriorated to 0.02 or less
	2-2	Those who are left with serious impairment in functions of the nervous system or psyche and require occasional nursing care
	2-3	Those who are left with serious impairment in functions of the thorax and abdominal organs and require occasional nursing care
	3	Those who have lost both upper limbs above the wrist joint
	4	Those who have lost both lower limbs above the ankle joint
Grade 3	1	Those who are blind in one eye and whose vision has deteriorated to 0.06 or less in the other eye
	2	Those who have lost the functions of mastication or speech
	3	Those who are left with serious impairment in functions of the nervous system or psyche and will be unable to engage in labor for the rest of their lives
	4	Those who are left with serious impairment in functions of the thorax and abdominal organs and will be unable to engage in labor for the rest of their lives
	5	Those who have lost all fingers of both hands
Grade 4	1	Those whose vision in both eyes has deteriorated to 0.06 or less
	2	Those who are left with serious impairment in the functions of mastication and speech
	3	Those who have completely lost hearing in both ears
	4	Those who have lost one upper limb above the elbow joint
	5	Those who have lost one lower limb above the knee joint
	6	Those who have lost the use of all fingers of both hands
	7	Those who have lost both feet above Lisfranc's joint
Grade 5	1	Those who are blind in one eye and whose vision has deteriorated to 0.1 or less in the other eye
	1-2	Those who are left with serious impairment in functions of the nervous system or psyche and will be unable to engage in any labor, excluding especially light labor
	1-3	Those who are left with serious impairment in functions of the thorax and abdominal organs and will be unable to engage in any labor, excluding especially light labor
	2	Those who have lost one upper limb above the wrist joint
	3	Those who have lost one lower limb above the ankle joint
	4	Those who have completely lost the use of one upper limb
	5	Those who have completely lost the use of one lower limb
	6	Those who have lost all toes of both feet

Grade		Physical Disability
Grade 6	1	Those whose vision in both eyes has deteriorated to 0.1 or less
	2	Those who are left with serious impairment in the functions of mastication or speech
	3	Those whose hearing in both ears has deteriorated to the extent that they cannot hear a loud voice unless they are right next to the said voice
	3-2	Those who have completely lost hearing in one ear and whose hearing in the other ear has deteriorated to the extent that they cannot hear an ordinary voice at a distance of ≥40 cm with that ear
	4	Those who are left with a serious deformity or mobility impairment in the spinal column
	5	Those who have lost the use of two of the three major joints in one upper limb
	6	Those who have lost the use of two of the three major joints in one lower limb
	7	Those who have lost the five fingers of one hand or four fingers of one hand including the thumb
Grade 7	1	Those who are blind in one eye and whose vision has deteriorated to 0.6 or less in the other eye
	2	Those whose hearing in both ears has deteriorated to the extent that they cannot hear an ordinary voice at a distance of ≥40 cm
	2-2	Those who have completely lost the hearing in one ear and whose hearing in the other ear has deteriorated to the extent that they cannot hear an ordinary voice at a distance of ≥1 m with that ear
	3	Those who are left with impairment in functions of the nervous system or psyche and cannot engage in any labor, excluding light labor
	4	Deleted
	5	Those who are left with impairment in functions of the thorax and abdominal organs and cannot engage in any labor, excluding light labor
	6	Those who have lost the thumb and two fingers of one hand or four fingers except the thumb of one hand
	7	Those who have lost the use of five fingers of one hand or four fingers of one hand including the thumb
	8	Those who have lost one foot above Lisfranc's joint
	9	Those who are left with pseudarthrosis in one upper limb and serious mobility impairment
	10	Those who are left with pseudarthrosis in one lower limb and serious mobility impairment
	11	Those who have lost the use of all toes of both feet
	12	Those who are left with extreme deformities in their external appearance
	13	Those who have lost both testicles
Grade 8	1	Those who are blind in one eye or whose vision has deteriorated to 0.02 or less in one eye
	2	Those who are left with mobility impairment in the spinal column
	3	Those who have lost the thumb and one finger of one hand or three fingers except the thumb of one hand
	4	Those who have lost the use of the thumb and two fingers of one hand or four fingers except the thumb of one hand
	5	Those who have had one lower limb shortened by ≥5 cm
	6	Those who have lost the use of one of the three major joints in one upper limb
	7	Those who have lost the use of one of the three major joints in one lower limb
	8	Those who are left with pseudarthrosis in one upper limb
	9	Those who are left with a pseudarthrosis in one lower limb
	10	Those who have lost all toes of one foot

Grade	Physical Disability	
Grade 9	1	Those whose vision in both eyes has deteriorated to 0.6 or less
	2	Those whose vision in one eye has deteriorated to 0.06 or less
	3	Those who are left with hemianopsia, contraction of the field of vision, or distortion of the field of vision in both eyes
	4	Those who are left with serious damage to both eyelids
	5	Those who have lost their noses and are left with serious impairment in function of the nose
	6	Those who are left with impairment in the functions of mastication and speech
	6-2	Those whose hearing in both ears has deteriorated to the extent that they cannot hear an ordinary voice at a distance of ≥1 m
	6-3	Those whose hearing has deteriorated in one ear to the extent that they cannot hear a loud voice unless they are right next to that ear and whose hearing in the other ear has decreased to the extent that they have difficulty in hearing an ordinary voice at a distance of ≥1 m
	7	Those who have completely lost the hearing in one ear
	7-2	Those who are left with impairment in the functions of the nervous system or psyche and for whom the labor in which they can engage is limited to a considerable extent
	7-3	Those who are left with impairment in functions of the thorax and abdominal organs and for whom the labor in which they can engage is limited to a considerable extent
	8	Those who have lost the thumb of one hand or two fingers except the thumb of one hand
	9	Those who have lost the use of two fingers of one hand including the thumb or three fingers except the thumbs of one hand
	10	Those who have lost two toes or more of one foot including the large toe
	11	Those who have lost the use of all toes of one foot
	11-2	Those who are left with considerable deformities in their external appearance
	12	Those who are left with serious impairment in the genital organs
Grade 10	1	Those whose vision in one eye has deteriorated to ≤0.1
	1-2	Those who are left with diplopia in their frontal vision
	2	Those who are left with impairment in the functions of mastication and speech
	3	Those who have 14 teeth or more replaced with dental prostheses
	3-2	Those whose hearing in both ears has deteriorated to the extent that they cannot hear an ordinary voice at a distance of ≥1 m
	4	Those whose hearing in one ear has deteriorated to the extent that they cannot hear a loud voice unless they are right next to the said voice
	5	Deleted
	6	Those who have lost the use of their thumb on one hand or two fingers except the thumb on one hand
	7	Those who have had one lower limb shortened by ≥3 cm
	8	Those who have lost the big toe or the other four toes on one foot
	9	Those who are left with serious impairment in function of one of the three major joints of one upper limb
	10	Those who are left with serious impairment in function of one of the three major joints of one lower limb

**Grade**	**Physical Disability**	
Grade 11	1	Those who are left with serious impairment in their adjustment function or mobility impairment in both eyeballs
	2	Those who are left with serious mobility impairment in both eyelids
	3	Those who are left with serious damage to one eyelid
	3-2	Those who have 10 teeth or more replaced with dental prostheses
	3-3	Those whose hearing in both ears has deteriorated to the extent that they cannot hear a low voice at a distance of ≥1 m
	4	Those whose hearing in one ear has deteriorated to the extent that they cannot hear an ordinary voice at a distance of ≥40 cm with that ear
	5	Those who are left with a deformity in their spinal column
	6	Those who have lost the index, third, or fourth finger on one hand
	7	Deleted
	8	Those who have lost the use of two toes or more on one foot including the big toe
	9	Those who are left with impairment in functions of the thorax and abdominal organs and such condition interferes with the performance of labor to a considerable extent
Grade 12	1	Those who are left with serious impairment in their adjustment function or mobility impairment in one eyeball
	2	Those who are left with serious mobility impairment in one eyelid
	3	Those who have seven teeth or more replaced with dental prostheses
	4	Those who have lost the major part of the auricle of one ear
	5	Those who are left with a serious deformity in the collarbone, breastbone, rib, shoulder blade, or pelvis
	6	Those who are left with impairment in the functions of one of the three major joints of one upper limb
	7	Those who are left with impairment in the functions of one of the three major joints of one lower limb
	8	Those who are left with a long bone deformity
	8-2	Those who have lost the little finger on one hand
	9	Those who have lost the use of the index, third, or fourth finger on one hand
	10	Those who have lost their second toe on one foot, those who have lost two toes including their second toe, or those who have lost three toes other than their big and second toes
	11	Those who have lost the use of their big toe or the other four toes on one foot
	12	Those who are left with obstinate localized nervous system symptoms
	13	Deleted
	14	Those who are left with deformities in their external appearance

Grade		Physical Disability
Grade 13	1	Those whose vision in one eye has deteriorated to ≤0.6
	2	Those who are left with hemianopsia, contraction of the field of vision, or distortion of the field of vision in one eye
	2-2	Those who are left with diplopia in their vision other than frontal vision
	3	Those who are left with damage to parts of both eyelids or have eyelash baldness
	3-2	Those who have five teeth or more replaced with dental prostheses
	3-3	Those who are left with an impairment in the thorax and abdominal organs
	4	Those who have lost the use of their little finger on one hand
	5	Those who have lost some of the bones of the thumb on one hand
	6	Deleted
	7	Deleted
	8	Those who have had one lower limb shortened by ≥1 cm
	9	Those who have lost one or two toes on one foot other than the big and second toes
	10	Those who have lost the use of their second toe on one foot, those who have lost the use of two toes including their second toe, or those who have lost the use of three toes other than the big and second toes
Grade 14	1	Those who are left with damage to a part of one eyelid or have eyelash baldness
	2	Those who have three teeth or more replaced with dental prostheses
	2-2	Those whose hearing in one ear has deteriorated to the extent that they cannot hear a low voice at a distance of ≥1 m with that ear
	3	Those who are left with deformed scars the size of their palm on the exposed surfaces of their upper limbs
	4	Those who are left with deformed scars the size of their palm on the exposed surfaces of their lower limbs
	5	Deleted
	6	Those who have lost some of the bones of their fingers except the thumb on one hand
	7	Those who have become unable to extend and contract the distal interphalangeal joint of any finger except the thumb on one hand
	8	Those who have lost the use of one or two toes on one foot other than the big and second toes
	9	Those who are left with localized nervous system symptoms
	10	Deleted

*1 Vision shall be measured in accordance with international visual acuity measurement standards. The vision of those with some abnormality in refraction shall be measured in relation to corrected vision.
*2 Those who have lost fingers means those who have lost, for the thumb, the part upward of the interphalangeal joint, and, for the other fingers, the parts upward of the proximal interphalangeal joint.
*3 Those who have lost the use of fingers means those who have lost half or more of the distal phalanges or those who are left with serious mobility impairment to the metacarpophalangeal joints or the proximal interphalangeal joints (for the thumb, the interphalangeal joint).
*4 Those who have lost toes means those who have lost all toes.
*5 Those who have lost use of the toes means those who have lost, for the large toe, half or more of the distal phalanges, and, for the other toes, the part above the distal interphalangeal joint or those who are left with serious mobility impairment in the metatarsophalangeal joints or the proximal interphalangeal joints (for the large toe, the interphalangeal joint).

End of Document
